# Adverse Event Costs and Cost-Effectiveness Analyses of Anticancer Drugs: A Systematic Review

**DOI:** 10.1001/jamanetworkopen.2025.12455

**Published:** 2025-05-27

**Authors:** Mingye Zhao, Taihang Shao, Yue Yin, Hongshu Fang, Hanqiao Shao, Wenxi Tang

**Affiliations:** 1 Department of Pharmacoeconomics, School of International Pharmaceutical Business, China Pharmaceutical University, Nanjing, Jiangsu, China; 2 Center for Pharmacoeconomics and Outcomes Research, China Pharmaceutical University, Nanjing, Jiangsu, China; 3 JC School of Public Health and Primary Care, Faculty of Medicine, The Chinese University of Hong Kong, Hong Kong, China; 4 Department of Public Affairs Management, School of International Pharmaceutical Business, China Pharmaceutical University, Nanjing, Jiangsu, China

## Abstract

**Importance:**

Accurately quantifying adverse event (AE) costs is essential for cost-effectiveness analyses (CEAs) of anticancer drugs. Misestimates in AE costs may significantly affect cost-effectiveness conclusions.

**Objective:**

To assess whether AE cost quantification in anticancer drug CEAs accurately reflects the true cost of AEs and to evaluate whether replacing AE costs with actual values affects cost-effectiveness conclusions.

**Evidence Review:**

A systematic search of PubMed, Web of Science, and Tufts CEA databases was conducted from October 24 to December 1, 2023, with an additional search from November 4 to 10, 2024, for English-language CEAs and claims-based studies examining AE costs for anticancer drugs published between January 2003 and December 2023. Claims-based AE costs were considered to represent actual values. AE costs were compared in absolute terms and as a proportion of total medical costs. Impact of replacing CEA AE cost estimates with actual values for incremental cost-effectiveness ratios (ICERs) was examined at thresholds of $100 000 and $150 000 per quality-adjusted life year (QALY). AE cost differences between CEA estimates and actual values and their impact on ICERs were the main outcomes.

**Findings:**

The sample included 11 claims-based US studies with 34 022 patients and 102 US payer-perspective CEAs. AE cost estimates in CEAs were consistently lower than actual values, with a median difference of 9.73% (IQR, 5.15%-27.22%; *P* = .002) in proportion of total medical costs and of $17 201 (IQR, $13 365-$48 970; *P* = .03) in absolute costs. Adjusting AE costs led to an ICER change of $42 656 per QALY, altering cost-effectiveness conclusions in 8 of 17 cases (47.1%). Among the 102 CEAs, 41 (40.2%) did not report AE types; of the remaining 61 (59.8%), 48 (78.7%) focused on treatment-related AEs instead of all-cause AEs. Of all CEAs, 79 (77.5%) considered grade 3 or higher AEs, ignoring grades 1 and 2. Only 13 studies (12.7%) accounted for AE-related dose reductions or interruptions, 87 (85.3%) did not consider postprogression AE costs, and 77 (82.8%) assumed AEs occurred only in the first treatment cycle. Substantial variability was observed in both drug AE and unit AE costs across studies.

**Conclusions and Relevance:**

In this systematic review of AE costs in oncology CEAs, AE costs were frequently underestimated, potentially altering cost-effectiveness conclusions. Key problems included incomplete AE inclusion, inaccurate AE cost estimates, overlooked long-term AEs, and unaccounted dose modifications. Best practices and standardized guidelines should be established to improve AE cost quantification in oncology CEAs.

## Key Points

**Question:**

Are adverse event (AE) costs in cost-effectiveness analyses (CEAs) of anticancer drugs consistent with actual values from claims-based studies?

**Findings:**

In this systematic review of 11 claims-based studies of AE costs and 102 CEAs, the proportion of AE costs among total medical costs was 10% higher and absolute costs $17 201 higher in claims-based studies than in CEAs, both statistically significant. Replacing AE costs in CEAs with actual values changed cost-effectiveness conclusions in 47% of cases.

**Meaning:**

The findings suggest that underestimating AE costs in oncology CEAs affects cost-effectiveness conclusions, highlighting the need for standardized guidelines.

## Teaser

This systematic review examines whether adverse event costs in cost-effectiveness analyses of anticancer drugs accurately reflect costs reported in claims-based studies.

## Introduction

According to the World Health Organization, cancer is the second leading cause of death worldwide, accounting for nearly 10 million deaths in 2020.^1^ Around 20% of patients with cancer experience severe adverse events (AEs), affecting quality of life and treatment adherence.^1,2^ Common AEs (eg, anemia, nausea, and fatigue^2^) may lead to treatment interruptions or medication changes, reducing efficacy.^3^ Managing AEs also requires additional medical resources, including medications, hospital stays, and tests, significantly increasing treatment costs.^4,5^

Cost-effectiveness analysis (CEA) is vital for assessing the cost-effectiveness of interventions, especially in resource-limited health care systems.^6,7^ Accurate quantification of AE costs is critical for comprehensive anticancer drug CEAs.^8,9^ Incorporating AE costs significantly impacts CEA outcomes, influencing both clinical and policy decisions.^10^

CEAs of anticancer drugs face specific challenges in quantifying AE costs (eMethods 1 in Supplement 1 gives details). One challenge is AE inclusion; determining which AEs (treatment related or all-cause) to include significantly affects cost estimates. For example, the cost per patient receiving immunotherapy for grade 3 and 4 all-cause AEs was found to be $4508 compared with $2722 for treatment-related AEs.^11^ Severity also matters; studies typically focus on grade 3 and 4 AEs due to higher hospitalization costs, excluding grades 1 and 2 as clinically minimal. Incidence thresholds vary, with some studies excluding low-incidence AEs to focus on those with greater cost impact. Additionally, postprogression AEs, differing by treatment regimen, are often overlooked but can substantially affect costs. A second challenge is dose reductions and interruptions; AEs causing drug pauses or dose reductions impact cost estimates and patient outcomes. Up to 56% of patients may receive less than 85% of the planned dose, impacting overall survival and progression-free survival.^12^ A third challenge is AE cost calculation; AE costs are typically derived by multiplying unit costs by occurrence probabilities. AE cost calculation methods vary, with unit costs sourced from literature, microcosting, or claims data.^13^ Simplified models often assume that AEs occur only once, in the first treatment cycle, potentially underestimating long-term costs, while complex models use cycle-based probabilities to dynamically estimate AE costs.

In CEAs, simplifying assumptions for AE cost quantification due to data limitations potentially causes discrepancies between estimated and actual AE costs. However, no studies to our knowledge have yet evaluated the accuracy of AE cost quantification in antineoplastic drug CEAs or its impact.^13^ We systematically reviewed key AE cost quantification techniques, compared CEA estimates with actual values, and assessed the impact of AE cost differences on incremental cost-effectiveness ratios (ICERs). We also provide recommendations and insights for future guidelines.

## Methods

For this systematic review, we conducted a systematic search from October 24 to December 1, 2023, with an additional search from November 4 to 10, 2024. The study followed the Preferred Reporting Items for Systematic Reviews and Meta-Analyses (PRISMA) reporting guideline.^14^

### Literature Search

Claims-based data on costs were considered reliable for drug AE costs, as they originated from actual insurance claims covering hospitalization, outpatient services, medications, and other health care costs. These data accurately reflect patient expenses and resource utilization.^15,16^ A systematic literature search was conducted in PubMed and Web of Science using the keywords *real-world*, *retrospective*, *observational*, *cancer*, *oncology*, *cost*, *economic burden*, and *adverse events* in titles or abstracts. The search was limited to English-language articles published between January 2003 and December 2023. The search strategy is given in eMethods 2 in Supplement 1.

Inclusion criteria for claims-based studies were publication from 2003 to 2023, a focus on AE-related costs in cancer drug treatment, use of claims-based data, involvement of US patients with cancer with defined treatment lines, and reporting of overall AE-related costs. Studies were excluded if they were modeling based, lacked relevant cost data, had insufficient cost observation periods to comprehensively quantify AE-related costs, or focused on nonmedical direct costs. We cross-checked references of included articles and used the Similar Articles feature in PubMed to identify additional relevant studies.

Based on indications from included claims-based studies, we searched the Tufts CEA database for US studies (from January 2003 onward) and supplemented with PubMed for post-2021 research. A systematic search was conducted using the following keywords: *economic evaluation*, *cost-effectiveness analysis*, *cancer*, and *adverse events*. The search excluded reviews, clinical trials, and randomized clinical trials (RCTs) and was limited to articles published between January 1, 2021, and December 1, 2023. The search strategy is given in eMethods 2 in Supplement 1. All included CEAs were applied in the literature review. Studies with absolute or proportional AE costs were used for quantitative comparison with claims-based studies.

Included CEAs were published from January 2003 to December 2023; were based on US payer perspectives; and had drug interventions, defined treatment lines, and indications matching those of claims-based studies. Non-CEA studies or studies from nonpayer perspectives were excluded.

### Data Extraction

For included claims-based studies, we extracted the PubMed identification number, year, tumor type, treatment regimen, data source, sample size, cost payer, AE inclusion criteria, AE-related costs, and other direct medical costs. For each CEA, we extracted the PubMed identification number, year, disease, treatment regimen, study perspective, AE inclusion criteria, cost calculation method, unit costs and sources, dose adjustment handling, and outcomes (AE costs, lifetime and treatment period costs, and quality-adjusted life years [QALYs]). Titles and abstracts were screened independently by M.Z. and T.S. based on predefined eligibility criteria. Full-text articles were then reviewed independently by both authors to confirm inclusion. Discrepancies were resolved by W.T. or through discussion.

### Study Outcomes

Outcomes in the systematic review of oncology CEAs were scope of AE-related costs, consistency of AE inclusion with reported data, AE cost quantification methods (including unit costs and sources), and handling of AE-related dose adjustments. Unit AE cost was the total treatment cost per AE occurrence. Other outcomes were direct cost differences (AE treatment cost [absolute value] and AE cost proportion [percentage of AE costs among total direct medical costs]), relative cost differences between treatments (as CEAs assess comparative drug effectiveness), and impact of discrepancies in AE cost estimation on ICERs.

### Statistical Analysis

We assessed the consistency of AE inclusion with reported data in CEAs by comparing the AE rates and types in CEA models or parameter tables with the criteria outlined in the articles’ methods sections, typically including data sources (eg, RCTs), AE types (eg, grade ≥3), and incidence thresholds (eg, 5%). Incidence was considered inconsistent if the AE incidence reported in the CEA differed from that in the cited RCT by more than 1%. The type of AEs included was deemed inconsistent if any AEs were added or omitted in the CEA compared with the cited RCT.

CEAs were matched to claims-based studies in cost comparisons. In the base case, costs were compared between treatments with the same mechanism, the same number of drugs in the treatments, and the same indication due to the limited availability of claims-based studies. AE treatment costs were sourced from CEAs and claims-based studies. If AE costs were not reported in CEAs, we recalculated them based on the article’s methods. Relative cost differences were calculated by comparing AE-related costs between treatments within the same study. For CEAs, postprogression costs were excluded to ensure consistency between AE and treatment costs. In studies without total treatment costs, we multiplied unit-time cost by treatment duration. AE cost proportion was calculated by dividing AE treatment costs by total direct medical costs, including drug, hospitalization, diagnostics, outpatient, nursing, and other related expenses. If total costs were unavailable, we used the ratio of mean cycle AE costs to average total cycle costs. Since some claims-based studies included both payer and patient costs, we assumed similar AE cost proportions for both based on patient costs being small (10%-15%) and studies showing comparable AE proportions (eg, 9.3% vs 9.0% for first-line advanced non–small-cell lung cancer treatment).^17,18^

Wilcoxon signed-rank exact test was used for comparison of nonnormally distributed data.^19,20^ A 2-sided significance level of *P* < .05 was applied. Data dispersion was assessed using the coefficient of variation and dispersion index, with high dispersion defined as coefficient of variation greater than 0.3 or dispersion index greater than 1.0.^21,22^ A detailed description of variables is given in eMethods 3 in Supplement 1.

We substituted AE costs in CEAs with actual costs to assess their impact on ICERs using reported total costs and effectiveness. ICER thresholds were set at $100 000 to $150 000.^23^ One claims-based study reported combined payer and patient costs, while others reported payer-only costs.^24^ For comparability, we assumed patient costs were negligible in the base-case analysis, based on Medicare studies showing that patients covered only about 16.7% of total AE costs.^17,18^ Additionally, the commercial payer study showed higher reimbursement rates than Medicare. Costs were adjusted to 2022 values using the Consumer Price Index.^25^ Analyses were conducted using R, version 4.2.1 (R Project for Statistical Computing).

Scenario analyses were conducted to validate base-case conclusions. In scenario 1, we refined the comparison to exactly the same drugs or combinations. In scenario 2, we assumed patients paid 10% of AE costs,^17,18^ given that 1 study^24^ reported combined payer and patient costs. In scenario 3, we assumed a 50% increase in AE costs, as a Canadian study reported a 14% higher severe AE incidence in the general population compared with clinical trials (27% median incidence).^26^ Jargon and abbreviations are defined in eMethods 4 in Supplement 1.

## Results

Eleven claims-based studies including 34 022 patients were included^17,18,24,27,28,29,30,31,32,33,34^ covering advanced non–small cell lung cancer, advanced melanoma, unresectable hepatocellular carcinoma, metastatic breast cancer, advanced renal cell carcinoma, resectable colorectal cancer, and nonmetastatic prostate cancer. Four studies (36.4%) reported absolute AE costs,^24,29,30,34^ and 9 (81.8%) reported AE costs as proportions of total costs.^17,18,27,28,29,30,31,32,33^ These 11 studies used claims databases like Medicare and commercial databases including all direct medical costs due to AEs, with almost all of the 11 studies (10 [90.9%]) including AEs of any grade.^18,24,27,28,29,30,31,32,33,34^ Based on indications in claims-based studies, we included 102 CEA articles from US payer perspectives,^35,36,37,38,39,40,41,42,43,44,45,46,47,48,49,50,51,52,53,54,55,56,57,58,59,60,61,62,63,64,65,66,67,68,69,70,71,72,73,74,75,76,77,78,79,80,81,82,83,84,85,86,87,88,89,90,91,92,93,94,95,96,97,98,99,100,101,102,103,104,105,106,107,108,109,110,111,112,113,114,115,116,117,118,119,120,121,122,123,124,125,126,127,128,129,130,131,132,133,134,135,136^ all using decision models. Most (96 [94.1%]) used partitioned survival or Markov models,^35,36,37,38,39,40,42,43,44,45,46,47,48,49,50,51,52,53,54,55,58,59,60,61,62,63,64,65,66,67,68,69,70,71,72,73,74,75,76,77,78,79,80,81,82,83,84,85,86,87,88,89,92,93,94,95,96,97,98,99,100,101,102,103,104,105,107,108,109,110,111,112,113,114,115,116,117,118,119,120,121,122,123,124,125,126,127,128,129,130,131,132,133,134,135,136^ with a few (6 [5.9%]) using microsimulation modeling.^41,56,57,90,91,106^ These studies covered all indications (including metastatic urothelial cancer) except nonmetastatic prostate cancer and resectable colorectal cancer. Tumor types and details of the 11 claims-based studies are reported in eTables 1 to 3 in Supplement 1 (eFigure 1 in Supplement 1 shows the screening flowchart). Details and the screening flowchart of the included CEAs are in eTable 4 and eFigure 2 in Supplement 1.

Of the 102 CEAs, 41 (40.2%) did not report AE types.^36,37,38,39,40,43,44,47,49,51,53,56,57,58,61,62,64,65,72,73,74,79,80,81,82,85,91,93,99,103,104,107,108,118,121,123,126,128,129,135,136^ Among the remaining 61 (59.8%),^35,41,42,45,46,48,50,52,54,55,59,60,63,66,67,68,69,70,71,75,76,77,78,83,84,86,87,88,89,90,92,94,95,96,97,98,100,101,102,105,106,109,110,111,112,113,114,115,116,117,119,120,122,124,125,127,130,131,132,133,134^ 48 (78.7%) used treatment-related AEs^41,42,46,48,50,52,54,55,59,60,66,68,69,71,75,76,77,78,83,84,86,87,88,94,95,96,97,98,100,101,102,105,106,109,110,111,112,114,115,119,120,122,125,127,131,132,133,134^ and 13 (21.3%) used all-cause AEs.^35,45,63,67,70,89,90,92,113,116,117,124,130^ A total of 79 CEAs (77.5%) considered grade 3 or higher AEs,^35,37,38,40,41,42,43,44,45,46,47,49,50,51,52,53,54,55,56,57,59,60,61,62,63,64,65,66,67,68,69,70,73,74,75,76,77,78,83,84,85,86,87,88,89,90,91,95,97,98,100,101,102,105,106,108,111,112,113,114,116,117,118,119,120,121,122,123,124,125,126,127,128,130,131,132,133,135,136^ 8 (7.8%) considered all grades of AEs,^36,48,72,94,103,104,109,110^ and 5 (4.9%) considered serious AEs.^82,96,107,115,134^ Four (3.9%) considered only specific AEs,^58,71,92,129^ and 6 (5.9%) did not consider AEs.^39,79,80,81,93,99^ For AE incidence rates, 35 CEAs (34.3%) considered any rate,^37,41,43,44,48,52,55,56,60,61,62,64,69,71,75,76,77,83,88,90,91,92,100,104,105,108,111,114,116,117,119,122,124,126,130^ 29 (28.4%) included AEs with rates above 5%,^35,45,46,49,53,59,63,67,68,70,74,86,89,94,95,96,98,102,103,113,120,125,127,128,131,132,134,135,136^ 12 (11.8%) set other thresholds,^47,50,51,54,66,78,84,87,107,112,115,133^ and 26 (25.5%) did not report incidence rates.^36,38,39,40,42,57,58,65,72,73,79,80,81,82,85,93,97,99,101,106,109,110,118,121,123,129^ For intergroup differences, 60 studies (58.8%) imposed no restrictions,^35,37,41,43,44,45,46,48,49,50,51,52,53,54,55,56,59,62,63,64,67,68,69,70,71,74,75,76,77,78,83,84,87,88,89,90,91,92,94,102,103,104,105,112,113,114,115,116,117,120,122,126,127,128,130,131,132,133,135,136^ 12 (11.8%) considered only notable differences,^47,60,61,95,96,100,108,111,119,124,125,134^ and 30 (29.4%) did not report handling of intergroup differences.^36,38,39,40,42,57,58,65,66,72,73,79,80,81,82,85,86,93,97,98,99,101,106,107,109,110,118,121,123,129^ According to the AE inclusion criteria of each of the 68 CEAs (66.7%) reviewed,^35,36,38,40,41,42,46,47,48,51,52,53,54,55,60,61,62,63,64,66,67,68,69,70,71,72,74,75,76,77,78,82,83,84,87,88,89,90,92,94,96,98,100,103,104,105,107,109,110,111,113,114,115,116,117,119,122,124,125,126,127,129,130,131,132,133,134,135^ 39 (57.4%) either missed or wrongly included AEs,^35,40,47,52,60,61,62,63,72,74,75,76,77,78,83,84,87,88,89,96,98,100,103,104,105,110,114,115,117,119,122,124,125,126,130,131,132,133,135^ 18 (26.5%) had issues with AE rates,^46,61,66,68,70,71,72,77,78,87,89,94,104,109,113,117,132,134^ and only 20 (29.4%) fully met the criteria.^36,38,41,42,48,51,53,54,55,64,67,69,82,90,92,107,111,116,127,129^ In addition, 87 (85.3%) did not consider postprogression AE costs.^35,36,37,38,39,40,42,43,44,45,46,47,48,49,50,51,52,53,54,55,58,61,62,63,64,65,66,67,68,69,70,71,74,75,76,77,78,79,80,82,83,84,85,86,88,89,91,92,93,94,95,96,97,98,99,100,101,102,103,105,107,108,109,110,111,112,113,114,115,117,118,119,121,122,123,124,125,126,128,129,130,131,132,133,134,135,136^

Of the 102 CEAs, only 13 (12.7%) considered impact of AEs on treatment interruptions or dose reductions,^42,54,67,70,72,79,92,101,102,120,124,125,126^ while 89 (87.3%) assumed 100% planned dosing.^35,36,37,38,39,40,41,43,44,45,46,47,48,49,50,51,52,53,55,56,57,58,59,60,61,62,63,64,65,66,68,69,71,73,74,75,76,77,78,80,81,82,83,84,85,86,87,88,89,90,91,93,94,95,96,97,98,99,100,103,104,105,106,107,108,109,110,111,112,113,114,115,116,117,118,119,121,122,123,127,128,129,130,131,132,133,134,135,136^ Almost all (96 [94.1%]) ignored the impact of dose adjustments on efficacy.^35,36,37,38,39,40,42,43,44,45,46,47,48,49,50,51,52,53,54,55,58,59,60,61,62,63,64,65,66,67,68,69,70,71,72,73,74,75,76,77,78,79,80,81,82,83,84,85,86,87,88,89,92,93,94,95,96,97,98,99,100,101,102,103,104,105,107,108,109,110,111,112,113,114,115,116,117,118,119,120,121,122,123,124,125,126,127,128,129,130,131,132,133,134,135,136^

Of the 93 CEAs (91.2%) considering AE-related costs with reported information,^35,36,37,38,40,41,42,43,44,45,46,47,48,49,50,51,52,53,54,55,56,59,60,61,62,63,64,65,66,67,68,69,70,71,72,73,74,75,76,77,78,79,80,81,82,83,84,85,86,87,88,89,90,91,92,94,95,96,98,100,101,102,103,104,105,106,107,108,111,112,113,114,115,116,117,118,119,120,121,122,123,124,125,126,127,128,130,131,132,133,134,135,136^ 77 (82.8%) assumed AEs occurred once in the first treatment cycle.^35,36,37,41,43,44,45,46,47,48,49,51,52,53,54,55,56,59,60,61,62,63,64,66,67,68,69,70,71,74,75,76,77,78,83,84,85,86,87,88,89,90,91,92,94,95,96,98,100,102,103,104,105,106,108,111,112,113,114,115,116,117,119,120,122,124,125,126,127,128,130,131,132,133,134,135,136^ In contrast, 10 studies (10.8%) used specific statistical distributions (eg, exponential) to convert incidence rates into cycle-specific probabilities.^40,42,50,72,81,82,101,107,118,123^ The remaining 6 studies (6.5%)^38,65,73,79,80,121^ directly used drug AE cost data from claims-based analyses or from other research, including modeling studies and CEAs (eTable 4 in Supplement 1).

We summarized unit AE costs in CEAs, differentiating between grade 1 and 2 and grade 3 or higher (eTables 5 and 6 in Supplement 1). Unit costs of common AEs (eg, diarrhea, fatigue, and neutropenia) except palmar-plantar erythrodysesthesia had a coefficient of variation exceeding 0.3, indicating high cost variability even within the same disease and treatment (eFigure 3 in Supplement 1). For example, the coefficient of variation for grade 3 or higher anemia unit costs ranged from 0.55 to 1.22 across all indications.

**Figure 1.**
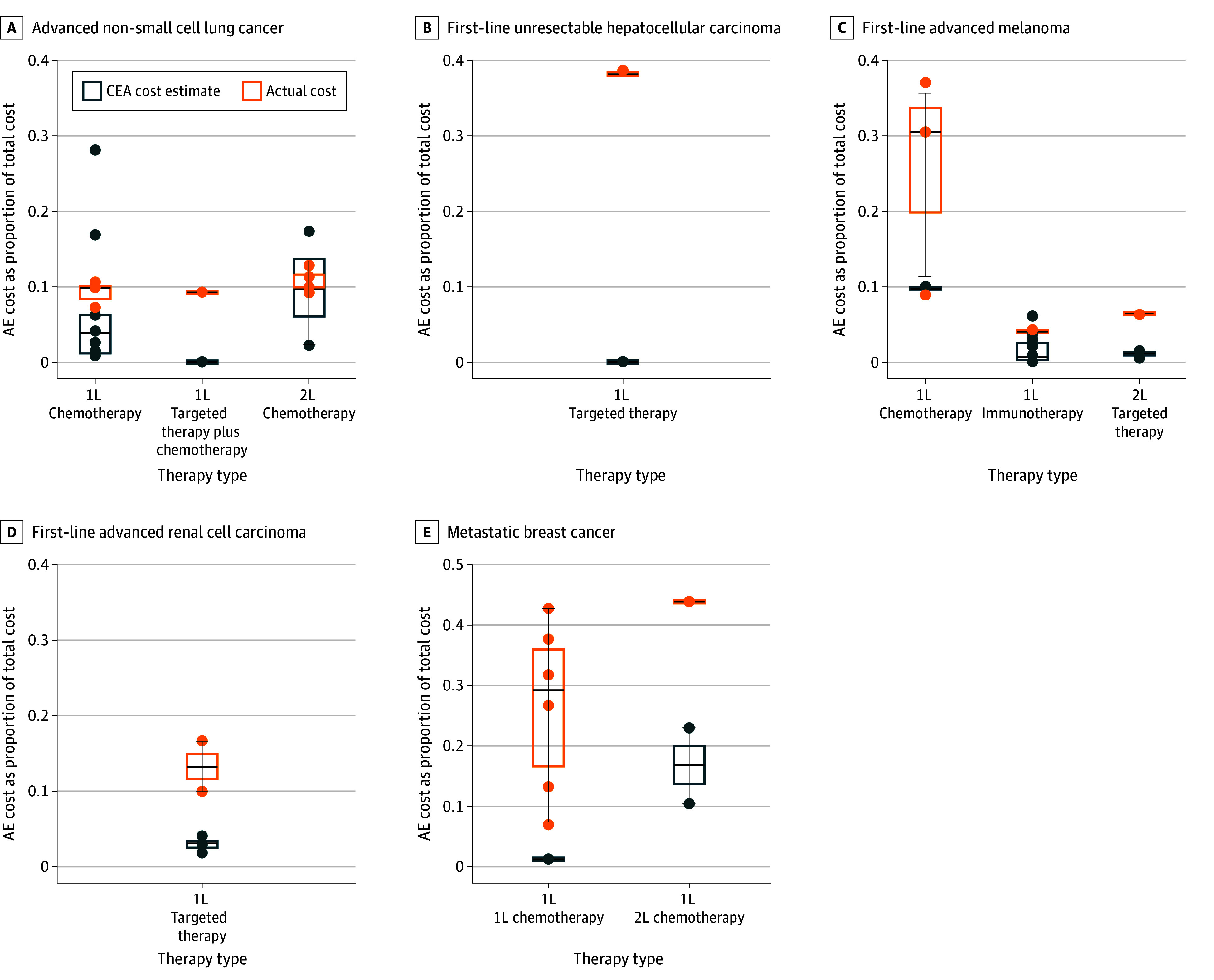
Difference in Adverse Event (AE) Cost Proportions Between Claims-Based Studies and Cost-Effectiveness Analyses Each cost-effectiveness analysis (CEA) data marker represents a comparison between the actual value and the CEA value estimate, conducted under the same treatment and indication. The horizontal bar inside the boxes indicates the median and the lower and upper ends of the boxes, the first and third quartiles. The whiskers extend to 1.5 times the IQR from quartiles, and data more extreme than the whiskers are plotted as outliers. The AE cost proportion was calculated by dividing AE treatment costs by total direct medical costs. 1L indicates first-line; 2L, second-line.

The 9 claims-based studies (81.8%)^17,18,24,27,29,30,31,32,33^ and 52 CEA studies (51.0%)^35,36,40,42,43,46,49,52,53,54,55,56,57,60,62,65,66,67,68,69,70,72,73,74,75,76,77,78,84,86,87,91,95,96,98,100,101,102,103,105,109,110,112,114,115,116,117,118,121,128,131,133^ included in AE cost or proportion comparisons are summarized in eTable 7 in Supplement 1, while those not included in quantitative comparisons (2 claims-based studies [18.2%]^28,34^ and 50 CEA studies [49.0%]^37,38,39,41,44,45,47,48,50,51,58,59,61,63,64,71,79,80,81,82,83,85,88,89,90,92,93,94,97,99,104,106,107,108,111,113,119,120,122,123,124,125,126,127,129,130,132,134,135,136^) and their exclusion reasons (eg, some CEA studies did not have claims-based studies for comparison) are listed in eTable 8 in Supplement 1. AE costs for each comparison in claims-based and CEA studies were comparable regarding study year, tumor types, treatment regimen, and cost follow-up duration (eTables 9 and 10 in Supplement 1 give details). Overall, proportions in claims-based studies were higher than CEA proportions by mechanism (median difference, 9.73% [IQR, 5.15%-27.22%]; *P* = .002) and by drug (median difference, 8.01% [IQR, 5.38%-27.13%]; *P* = .003). Stratified analyses by treatment and indication consistently showed higher proportions in claims-based studies than in CEAs (Figure 1 and the Table).

**Table.** Comparison of AE Costs Between CEA and Data From CBS^a^

Treatment	Value, median (IQR)		Difference	Studies used for analysis, No.^b^	
	CEA	CBS		CEA	CBS
**AE cost proportion by therapy mechanism, %^c^**					
First-line chemotherapy for ANSCLC	4.05 (1.63-6.32)	9.84 (8.41-10.01)	5.79	7	1
First-line targeted therapy plus chemotherapy for ANSCLC	0.07 (0.07-0.07)	9.32 (9.32-9.32)	9.25	1	1
Second-line chemotherapy for ANSCLC	9.94 (6.35-13.54)	10.61 (9.84-11.63)	0.67	1	1
First-line chemotherapy for MBC	1.17 (1.17-1.17)	29.34 (16.68-36.15)	28.17	1	3
Second-line chemotherapy for MBC	16.83 (13.71-19.96)	44.05 (44.05-44.05)	27.22	2	1
First-line ICI for AM	0.64 (0.36-2.81)	4.00 (4.00-4.00)	3.36	5	1
First-line targeted therapy for AM	1.25 (0.93-1.34)	6.40 (6.40-6.40)	5.15	1	1
First-line chemotherapy for AM	9.87 (9.87-9.87)	9.20 (3.40-36.90)	20.53	1	1
First-line targeted therapy for ARCC	3.12 (2.50-3.39)	13.30 (11.69-14.90)	10.18	2	1
First-line targeted therapy for UHCC	0.01 (0.01-0.01)	38.13 (38.13-38.13)	38.12	1	1
**AE cost proportion by drug, %^c^**					
First-line bevacizumab plus carboplatin and paclitaxel for ANSCLC	0.07 (0.07-0.07)	9.32 (9.32-9.32)	9.25	1	1
First-line platinum-based chemotherapy for ANSCLC	4.05 (1.63-6.32)	9.84 (8.41-10.01)	5.79	7	1
First-line taxane for MBC	1.17 (1.17-1.17)	29.34 (28.07-30.61)	28.17	1	2
Second-line docetaxel for ANSCLC	17.13 (17.13-17.13)	11.27 (11.27-11.27)	−5.86	1	1
Second-line pemetrexed for ANSCLC	2.75 (2.75-2.75)	9.52 (9.52-9.52)	6.77	1	1
Second-line taxane for MBC	16.83 (13.71-19.96)	44.05 (44.05-44.05)	27.22	2	1
First-line sorafenib for UHCC	0.01 (0.01-0.01)	38.13 (38.13-38.13)	38.12	1	1
First-line dacarbazine for AM	9.87 (9.87-9.87)	36.90 (36.90-36.90)	27.03	1	1
First-line ipilimumab for AM	0.40 (0.21-0.64)	4.00 (4.00-4.00)	3.60	5	1
First-line pazopanib for ARCC	3.66 (3.66-3.66)	10.09 (10.09-10.09)	6.43	1	1
First-line sunitinib for ARCC	2.50 (2.18-2.81)	16.51 (16.51-16.51)	14.01	2	1
First-line vemurafenib for AM	1.43 (1.43-1.43)	6.40 (6.40-6.40)	4.97	1	1
**AE cost by therapy mechanism, $**					
First-line chemotherapy for MBC	2441 (2203-2529)	14 118 (9812-18 425)	11 678	3	1
Second-line chemotherapy for MBC	13 619 (10 522-16 423)	28 672 (28 168-29 176)	15 053	6	1
First-line chemotherapy for ANSCLC	5025 (3132-9201)	26 690 (26 690-26 690)	21 665	26	1
First-line chemotherapy plus ICI for ANSCLC	5352 (2050-7610)	21 815 (21 815-21 815)	16 463	10	1
First-line ICI for ANSCLC	992 (270-1493)	18 930 (18 930-18 930)	17 938	14	1
First-line targeted therapy for UHCC	819 (15-3954)	77 094 (77 094-77 094)	76 275	5	1

Abbreviations: AE, adverse event; AM, advanced melanoma; ANSCLC, advanced non–small cell lung cancer; ARCC, advanced renal cell carcinoma; CBS, claims-based studies; CEA, cost-effectiveness analysis; ICI, immune checkpoint inhibitor; MBC, metastatic breast cancer; UHCC, unresectable hepatocellular carcinoma.

^a^ Comparisons between values from CBS and CEA were conducted for the same treatment and indication.

^b^ In the same study, results for multiple treatment approaches under the same therapy mechanism may have been reported. Details are given in eTables 2 to 4 in Supplement 1.

^c^ AE cost proportion was calculated by dividing AE treatment costs by total direct medical costs. Differences are shown as percentage points.

**Figure 2.**
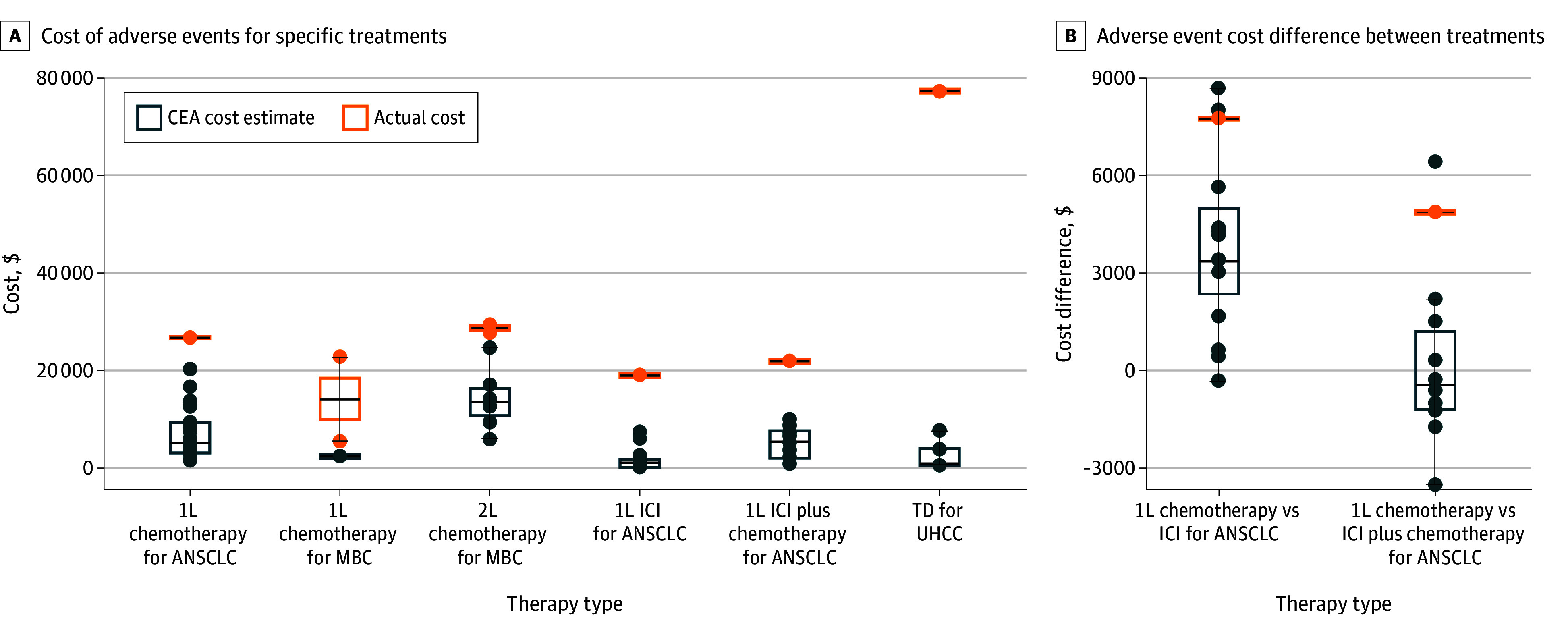
Differences in Absolute Adverse Event (AE) Costs and Between-Treatment Costs Between Claims-Bases Studies and Cost-Effectiveness Analyses Each CEA data marker represents a comparison between the actual value and the CEA value estimate, conducted under the same treatment and indication. The horizontal bar inside the boxes indicates the median and the lower and upper ends of the boxes, the first and third quartiles. The whiskers extend to 1.5 times IQR from quartiles, and data more extreme than the whiskers are plotted as outliers. 1L indicates first-line; 2L, second-line; ANSCLC, advanced non–small cell lung cancer; ICI, immune checkpoint inhibitor; MBC, metastatic breast cancer; TD, targeted drug; UHCC, unresectable hepatocellular carcinoma.

Differences in direct AE costs between claims-based studies and CEAs were as follows (Figure 2A and the Table). Overall, actual costs significantly exceeded CEA estimates, with a median difference of $17 201 (IQR, $13 365-$48 970; *P* = .03). Actual costs were also consistently higher than CEA estimates in every comparison. Due to limited data, we analyzed relative AE cost discrepancies only for first-line advanced non–small-cell lung cancer treatments. For chemotherapy vs immune checkpoint inhibitor, the difference between actual costs was $7760 and the mean (SD) difference between CEA cost estimates was $3719 ($2580); for chemotherapy vs immune checkpoint inhibitor plus chemotherapy, the difference between actual costs was $4875 and the mean (SD) difference between CEA cost estimates was $210 ($2714) (Figure 2B).

**Figure 3.**
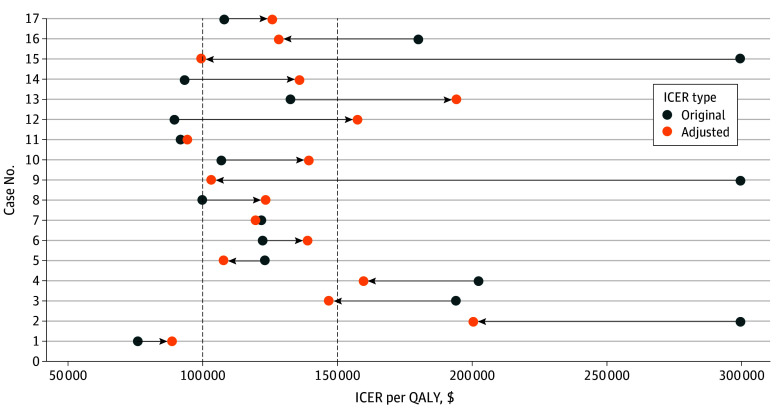
Adverse Event (AE) Cost Adjustment and Fluctuations in Incremental Cost-Effectiveness Ratios (ICERs) Per Quality-Adjusted Life Year The 17 cases were from 15 studies.^46,53,55,60,65,66,70,74,78,84,96,98,116,128,131^ Original ICER refers to the ICER reported in the cost-effectiveness analysis (CEA). Adjusted ICER reflects the ICER after replacing the AE cost estimate with data from claims-based studies. Vertical dashed lines represent thresholds for assessing ICER outcomes when replacing CEA cost estimates with costs from claims-based studies.

Median absolute ICER difference before and after AE cost adjustment was $42 656 per QALY (IQR, $16 604-$61 683 per QALY), with a 33.17% (95% CI, 20.20%-46.15%) mean absolute fluctuation. In 8 of 17 cases (47.1%) from 15 studies (14.7%),^46,53,55,60,65,66,70,74,78,84,96,98,116,128,131^ cost-effectiveness conclusions reversed at thresholds of $100 000 per QALY or $150 000 per QALY. Specifically, 6 studies (35.3%) reversed at $100 000 per QALY^60,74,78,84,98,128^ and 4 (23.5%) at $150 000 per QALY^60,70,74,98^ (Figure 3 and eTable 11 in Supplement 1).

Scenarios 1 to 3 confirmed robustness of base-case conclusions, with actual AE costs consistently exceeding CEA estimates both in proportion and in absolute value, affecting cost-effectiveness conclusions in half of cases (8 of 17 [47.1%]). Detailed results are in the eResults and eFigure 4 in Supplement 1.

**Figure 4.**
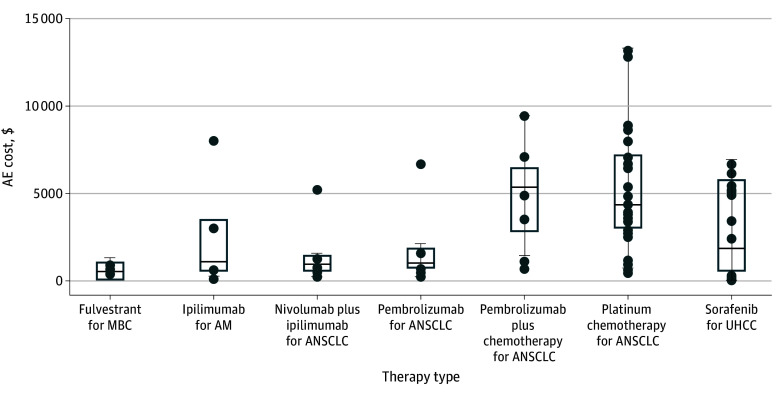
Variability in Cost of Adverse Events (AEs) by Treatment Type in Cost-Effectiveness Analyses (CEAs) Data markers represent AE treatment cost estimates of specific treatment options, which are all first-line therapies. The horizontal bar inside the boxes indicates the median and the lower and upper ends of the boxes, the first and third quartiles. The whiskers extend to 1.5 times IQR from quartiles, and data more extreme than the whiskers are plotted as outliers. AM indicates advanced melanoma; ANSCLC, advanced non–small cell lung cancer; MBC, metastatic breast cancer; UHCC, unresectable hepatocellular carcinoma.

AE cost estimates varied significantly across CEAs even for the same treatment under the same indication (Figure 4). In the 7 most data-rich cases, the coefficient of variation ranged from 0.62 (pembrolizumab plus chemotherapy) to 1.25 (pembrolizumab), with a median of 0.96 (fulvestrant). The median dispersion index ranged from 563.82 to 4615.27.

## Discussion

In this systematic review, AE costs reported in claims-based studies were higher than those reported in CEAs, with a median difference of 9.73% in total cost proportion and $17 201 in absolute costs. Adjusting ICERs with actual AE costs changed cost-effectiveness conclusions in 47.1% of CEAs. Our review of CEAs identified several issues: many studies did not specify AE types, focused on severe AEs, and ignored postprogression costs. Few considered dose modifications, treatment interruptions, or survival impacts. Most assumed AEs occurred only in the first treatment cycle, used inconsistent methods for estimating incidence and costs, and had substantial variability in unit AE costs.

Several studies have analyzed the quantification of AE-related costs in CEAs. Ghabri et al^13^ highlighted deficiencies in incorporating AEs into decision models, AE terminology, estimating AE impacts on quality of life and costs, uncertainty in the economic impact of AEs, and proposed improvements. Gyllensten et al^137^ comprehensively compared 5 methods for quantifying AE-related costs using individual data. Pearce et al^138^ reviewed economic evaluations of oncology drugs, proposing better AE quantification methods. Like our findings, their suggestions focused on AE inclusion, dose adjustments, multiple AEs, and effects on quality of life.^138^

Key reasons for lower AE costs in CEAs compared with claims-based studies may include AE inclusion issues, such as misclassification, omission of low-incidence or grade 1 and 2 AEs, and discrepancies between included AEs and stated criteria. Additionally, RCT-based CEAs may have short observation periods, missing long-term AEs. Other key reasons for the lower costs may be failure to consider AE-related dose modifications, leading to overestimated drug costs and underestimated AE cost impact; use of AE costs without verifying their accuracy; assuming AEs occur only once; and ignoring recurring AE costs. In addition, RCTs often enroll healthier patients with higher socioeconomic status, potentially contributing to lower AE costs in CEAs than in observational settings. These findings highlight the need to improve AE cost quantification in oncology CEAs and standardize methods for more accurate evaluations.

The International Society for Pharmacoeconomics and Outcomes Research recommends using treatment-related AE costs in CEAs to better capture treatment impact and avoid comorbidities.^139^ If all-cause AEs were inappropriately used, it could bias cost estimates. In the KEYNOTE-407 study, incidence of grade 3 or higher all-cause AEs was 15% to 17% higher than incidence of treatment-related AEs.^140^

Claims data showed significant costs for grade 1 and 2 AEs, such as $3940 for gastrointestinal perforation and $1934 for sepsis.^141^ Including all grades when data were available was recommended.^138^ Simplification is possible, but excluding all grade 1 and 2 AE costs is inadvisable due to their high incidence and cost. Grade 1 and 2 and grade 3 or higher AEs should be analyzed separately.^35,36^ For example, with a 50% incidence of sepsis (45% grade 1 and 2, 5% grade ≥3), costs are $23 510 for any grade, $1934 for grade 1 and 2, and $28 904 for grade 3 or higher. An any-grade approach estimated treatment costs at $12 750, but separating grades reduced this to $2316.^141^ Even slight evidence-quality improvement justifies the extra work.^142^ Simplifying models by excluding low-incidence AEs is inadvisable, as some low-incidence AEs still incur high costs.^143,144,145^ If exclusions are made, their rationale and impact should be clearly justified with uncertainty analyses.

In KEYNOTE-407, incidence of grade 3 or higher AEs for pembrolizumab plus chemotherapy was 74.8% worldwide but 86% in Asian patients.^140,146^ Therefore, in CEAs, safety data should align with the study population, ensuring consistency between AE incidence sources and research perspectives. Drug AE treatment costs and unit AE costs in this study varied significantly across CEAs, even under the same indication. Thus, the Consolidated Health Economic Evaluation Reporting Standards checklist should add detailed items to standardize practices.^6^ Researchers must justify data sources and conduct uncertainty analyses to avoid cherry picking.

In the CheckMate 227 trial for postprogression active treatment, nearly all immunotherapy patients received chemotherapy, while 80% in the chemotherapy group received subsequent immune checkpoint inhibitor treatment,^147^ with chemotherapy-related AE costs of $23 009 and immune checkpoint inhibitor costs of $16 319,^24^ showing a $4807 difference in subsequent AE costs. This underscores the importance of quantifying postprogression AE costs.

Our study found that only 13% of CEAs considered dose reductions and interruptions despite their prevalence and importance. The study by Diéras et al^148^ showed that 27.9% of patients receiving palbociclib had dose reductions and 66% had interruptions in the first 6 months. One meta-analysis reported average dose reductions in metastatic pancreatic cancer treatment of 75% for oxaliplatin, 74.5% for irinotecan, and 80.5% for continuous intravenous fluorouracil.^149^ Another review noted dose modifications in 20% to 62% of patients with colorectal cancer, 77% with non–small cell lung cancer, 60% with pancreatic cancer, and 33.6% with breast cancer.^150^ Lack of dose-adjustment quantification in CEAs potentially inflates total costs and skews economic conclusions.^138^ RCTs usually report overall rates, making treatment cycle–specific assessments difficult. We recommend using relative dose intensity to capture reductions and interruptions.^151^ Treatment outcomes may differ with dose adjustments.^152^ Subgroup data are often unavailable,^153^ so modeling survival with overall data may be unavoidable.

Most grade 3 or higher AEs occur within 1 to 2 weeks and last 6 to 12 days.^154,155^ Long-term studies showed that over 95% of severe AEs occurred in the first year, suggesting their costs may not require discounting.^154,156^ However, this becomes problematic for recurring grade 3 or higher AEs. Additionally, grade 1 and 2 AEs often recur after the first year.^154,156^ Future studies should use more clinical data to incorporate concurrent and recurring AEs into cost-effectiveness models.^138^ Exponential models are commonly used to simulate long-term AEs. However, making exponential assumptions will overestimate the AE costs for both grade 3 or higher AEs for which incidence peaks within 100 days and grade 1 and 2 AEs, like diarrhea, that typically occur within the first 6 months.^154^ While modeling AE incidence per treatment cycle has potential, it requires flexible models that account for AE severity and time-varying rates.

To address limitations of model-based AE cost quantification, CEAs should leverage high-quality clinical data. For drugs with such evidence, clinical data and standardized long-term electronic medical records should be prioritized, ensuring patient consistency. For innovative drugs lacking robust clinical data, health technology assessment agencies should reevaluate when evidence emerges to quantify AE impacts. Key points of the proposed good practice recommendations are summarized in eTable 12 in Supplement 1.

Discrepancies between efficacy in RCTs and actual clinical effectiveness are significant, as RCTs often have stricter inclusion criteria and higher adherence, potentially overestimating treatment benefits. Kumar et al^157^ found shorter survival among patients with cancer in the general population than in RCTs, emphasizing the importance of incorporating clinical data and sensitivity analyses in future CEAs to enhance accuracy and inform policy decisions.^157^

### Limitations

This study has limitations. First, claims-based AE cost data have limitations, such as coding errors, incomplete data, and lack of clinical context. Differences between general population and RCT settings could contribute to cost discrepancies. However, we cross-validated conclusions using multiple metrics and uncertainty analyses. Second, AE incidence in the general population may be higher than in trials, but scenario analyses showed that this did not affect our base-case conclusion. Third, since we used US data, future research should confirm whether these findings apply internationally. Fourth, due to limited data, we focused on common advanced cancers. Future studies should evaluate other cancers. Fifth, in our comparison, years in CEAs and claims-based cost analyses may not have fully aligned, with potential bias despite Consumer Price Index adjustments.

## Conclusion

In this systematic review of AE costs in CEAs of anticancer drugs, we found that AE costs were often underestimated, potentially reversing cost-effectiveness conclusions. Key problems included inconsistent AE inclusion, inadequate citation of unit costs, and challenges in quantifying multiple or long-term AE costs. Additionally, many CEAs neglected impacts of AE-related dose adjustments on costs and efficacy. Further research and guidelines are needed to enhance methods and provide evidence-based recommendations.

## Notes

Supplement 1. **eMethods 1.** Detailed Description of Challenges in Quantifying AE-Related Costs **eMethods 2.** PubMed Search Strategy for Real-World and CEA Studies Related to Antineoplastic Drugs **eMethods 3.** Distribution of All Variables in Our Study **eMethods 4.** Definitions of Jargon and Abbreviations **eResults.** Scenario Analysis Results **eFigure 1.** PRISMA Flowchart of Included Studies From Actual AE Cost Literature **eFigure 2.** PRISMA Flowchart of Included Studies From CEA Literature **eTable 1.** Key Information of 11 Included Real-World Studies **eTable 2.** Real-World Studies Reporting Absolute Drug AE Costs **eTable 3.** Real-World Studies Reporting Proportion of Drug AE Costs Out of Total Medical Costs **eTable 4.** Key Information From 102 Included CEA Studies **eTable 5.** Summary of Unit Costs for Grade 1 to 2 AEs in CEAs **eTable 6.** Summary of Unit Costs for Grade 3 or Higher AEs in CEAs **eTable 7.** Included Total-Cost and CEA Studies for Comparison of AE Cost or Cost Proportion Between CEAs and Real-World Data **eTable 8.** Real-World Studies and CEAs Not Included in the Comparison Between CEAs and Real-World Studies **eTable 9.** Comparability Analysis of Real-World and CEA Evidence for AE Cost Proportions **eTable 10.** Comparability Analysis of Real-World and CEA Evidence for Absolute AE Costs **eTable 11.** Detailed Information for Association of AE Cost With ICER **eFigure 3.** Variability in the Cost of Unit Adverse Events Among Cost-Effectiveness Analyses **eFigure 4.** Proportion of Adverse Event Costs for Specific Drugs in Cost-Effectiveness Analysis vs Real-World Values **eTable 12.** Good Practice Recommendations
eReferences

Supplement 2.
Data Sharing Statement
