# Supplementary material for: Adverse Event Costs and Cost-Effectiveness Analyses of Anticancer Drugs: A Systematic Review
Source: JAMA Netw Open. 2025 May 27;8(5):e2512455. doi: 10.1001/jamanetworkopen.2025.12455 (PMC12117467; doi:10.1001/jamanetworkopen.2025.12455)
Supplement: Supplement 1. — eMethods 1. Detailed Description of Challenges in Quantifying AE-Related Costs eMethods 2. PubMed Search Strategy for Real-World and CEA Studies Related to Antineoplastic Drugs eMethods 3. Distribution of All Variables in Our Study eMethods 4. Definitions of Jargon and Abbreviations eResults. Scenario Analysis Results eFigure 1. PRISMA Flowchart of Included Studies From Actual AE Cost Literature eFigure 2. PRISMA Flowchart of Included Studies From CEA Literature eTable 1. Key Information of 11 Included Real-World Studies eTable 2. Real-World Studies Reporting Absolute Drug AE Costs eTable 3. Real-World Studies Reporting Proportion of Drug AE Costs Out of Total Medical Costs eTable 4. Key Information From 102 Included CEA Studies eTable 5. Summary of Unit Costs for Grade 1 to 2 AEs in CEAs eTable 6. Summary of Unit Costs for Grade 3 or Higher AEs in CEAs eTable 7. Included Total-Cost and CEA Studies for Comparison of AE Cost or Cost Proportion Between CEAs and Real-World Data eTable 8. Real-World Studies and CEAs Not Included in the Comparison Between CEAs and Real-World Studies eTable 9. Comparability Analysis of Real-World and CEA Evidence for AE Cost Proportions eTable 10. Comparability Analysis of Real-World and CEA Evidence for Absolute AE Costs eTable 11. Detailed Information for Association of AE Cost With ICER eFigure 3. Variability in the Cost of Unit Adverse Events Among Cost-Effectiveness Analyses eFigure 4. Proportion of Adverse Event Costs for Specific Drugs in Cost-Effectiveness Analysis vs Real-World Values eTable 12. Good Practice Recommendations eReferences [file jamanetwopen-e2512455-s001.pdf]

Supplementary Online Content

Zhao M, Shao T, Yin Y, Fang H, Shao H, Tang W. Adverse event costs and impact in cost-effectiveness analyses of anticancer drugs: a systematic review. *JAMA Netw Open*. 2025;8(5):e2512455. doi:10.1001/jamanetworkopen.2025.12455

eMethods 1. Detailed Description of Challenges in Quantifying AE-Related Costs
eMethods 2. PubMed Search Strategy for Real-World and CEA Studies Related to Antineoplastic Drugs
eMethods 3. Distribution of All Variables in Our Study
eMethods 4. Definitions of Jargon and Abbreviations
eResults. Scenario Analysis Results
eFigure 1. PRISMA Flowchart of Included Studies From Actual AE Cost Literature
eFigure 2. PRISMA Flowchart of Included Studies From CEA Literature
eTable 1. Key Information of 11 Included Real-World Studies
eTable 2. Real-World Studies Reporting Absolute Drug AE Costs
eTable 3. Real-World Studies Reporting Proportion of Drug AE Costs Out of Total Medical Costs
eTable 4. Key Information From 102 Included CEA Studies
eTable 5. Summary of Unit Costs for Grade 1 to 2 AEs in CEAs
eTable 6. Summary of Unit Costs for Grade 3 or Higher AEs in CEAs
eTable 7. Included Total-Cost and CEA Studies for Comparison of AE Cost or Cost Proportion Between CEAs and Real-World Data
eTable 8. Real-World Studies and CEAs Not Included in the Comparison Between CEAs and Real-World Studies
eTable 9. Comparability Analysis of Real-World and CEA Evidence for AE Cost Proportions
eTable 10. Comparability Analysis of Real-World and CEA Evidence for Absolute AE Costs
eTable 11. Detailed Information for Assocation of AE Cost With ICER
eFigure 3. Variability in the Cost of Unit Adverse Events Among Cost-Effectiveness Analyses
eFigure 4. Proportion of Adverse Event Costs for Specific Drugs in Cost-Effectiveness Analysis vs Real-World Values
eTable 12. Good Practice Recommendations
eReferences

This supplementary material has been provided by the authors to give readers additional information about their work.

© 2025 Zhao M et al. *JAMA Network Open*.

eMethods 1. Detailed Description of Challenges in Quantifying AE-Related Costs

Note: This section provides an overview of key challenges in quantifying AE costs in CEAs. While these challenges are not the primary focus of our study, their potential impact on cost-effectiveness conclusions is thoroughly discussed in the Discussion section, where we also offer recommendations for improving AE cost estimation. A summary of potential solutions to these challenges, along with suggested best practices, is provided in **eTable 12**.

A. Inclusion of Adverse Events

Inclusion of AEs involves two aspects: the types of AEs included and the incidence rates of AEs. In economic evaluations, inclusion criteria for AEs vary.

Firstly, although it is recommended to use treatment-related AEs (TRAEs), some studies consider all-cause AEs in CEAs. The costs for treating TRAEs and all-cause AEs may differ significantly for the same drug. A study on Advanced Renal Cell Carcinoma showed that for immunotherapy, the per-patient costs for grade 3/4 all-cause AEs were $4508, while treatment-related AEs cost $2722[1].

Secondly, severity levels are considered; according to Common Terminology Criteria for Adverse Events (CTCAE) guidelines[2]. AEs are categorized from Grade 1 to 4, with Grade 5 being fatal. Researchers often focus on Grades 3-4 due to the need for hospitalization and treatment, ignoring Grades 1-2 as patients typically recover without significant intervention.

Thirdly, incidence thresholds vary; some studies only include AEs above a certain incidence rate, as lower incidence AEs have limited impact. Additionally, some studies focus on AEs with significant differences between groups to emphasize impactful results in CEAs. Additionally, AE costs for alternative treatments post-progression should be considered. Although most studies do not account for this, post-progression treatments differ between regimens (e.g., chemotherapy after immunotherapy, or vice versa). These AE costs can impact cost estimations between regimens.

B. Adverse Events-related Dose Reductions and Interruptions

When AEs occur, especially those of Grade 3 or higher, they incur treatment costs and affect the patient's medication regimen, including drug pauses or dose reductions until the AEs are controlled[3]. These factors impact cost estimations. A study found that 52% of chronic myeloid leukemia (CML) patients on dasatinib and 36% on imatinib experienced dose reductions or interruptions due to AEs[4]. Dose intensity served as a metric for evaluating dose modifications and treatment discontinuation, calculated as the ratio of the actual dose administered over a period to the planned dose. Studies indicated that up to 56% of patients received less than 85% of the planned dose due to adjustments made in response to adverse events. Dose adjustments can also affect patient survival, impacting cost estimates[5]. A breast cancer study showed that patients receiving high-dose intensity alpelisib had a 30% longer median PFS compared to those with low-dose intensity[6].

C. Methods for Calculating AE Costs[7] and Sources of Unit AE Costs

The method for calculating AE costs typically involves multiplying the unit cost by the occurrence probability. For a specific drug, the total AE cost is the sum of the costs for each AE. Differences in methods arise from these two components.

First is the source of AE unit costs. Sources of unit costs include[8]: (1) Literature, which is convenient but may lack generalizability and recency; (2) Micro-costing, providing accurate estimates but is time-intensive; (3) Guidelines/Clinical Consensus, ensuring validity but missing some costs and variations; and (4) Claims-Based Approach, offering comprehensive estimates but lacking AE severity details and

being time-consuming. Balancing these methods involves weighing detail and precision against effort and resources.

The second aspect concerns handling the probability of AEs, typically derived from clinical trials. Economic evaluations often assume all AEs occur once in the first cycle, simplifying models and calculations, especially since trials usually report only AE frequency, not occurrence numbers[9, 10]. However, this approach has significant drawbacks: it overlooks the repeated and varying durations of AEs, which can bias model results and underestimate the long-term cost impact on patients[11]. An alternative is the cycle-based AE probability method, where AEs can occur in any cycle, reflecting clinical reality more accurately. This approach uses incidence rates from RCTs across cycles, offering dynamic adjustments and comprehensive evaluations of long-term outcomes, costs, and quality of life. However, it increases model complexity and data requirements, making it suitable for long-term treatments, frequent AEs, and dynamic adjustments.

eMethods 2. PubMed Search Strategy for Real-World and CEA Studies Related to Antineoplastic Drugs

(1) Real-World Adverse Event Costs

Pubmed

(Real-world[Title/Abstract] OR Real world[Title/Abstract] OR Real world[Title/Abstract]OR Medicare[Title/Abstract] OR retrospective[Title/Abstract] OR database[Title/Abstract]) AND (cancer[Title/Abstract] OR Malignant[Title/Abstract] OR carcinoma[Title/Abstract] OR oncology[Title/Abstract] OR lymphoma[Title/Abstract] OR Melanoma[Title/Abstract]) AND (cost[Title/Abstract] OR economic[Title/Abstract] OR costs[Title/Abstract] OR burden[Title/Abstract]) AND (adverse event[Title/Abstract] OR toxicity[Title/Abstract] OR toxicities[Title/Abstract] OR adverse events[Title/Abstract]) AND ("2003/01/01"[Date - Publication] : "2023/12/1"[Date - Publication])

Web of Science

TS=("Real-world" OR "Real world" OR Medicare OR retrospective OR database)
AND TS=(cancer OR Malignant OR carcinoma OR oncology OR lymphoma OR Melanoma)
AND TS=(cost OR economic OR costs OR burden)
AND TS=("adverse event" OR toxicity OR toxicities OR "adverse events")
AND DT=(article)
AND CU=(USA)
AND LA=(English)
AND PY=(2003-2023)

(2) Adverse Event Costs in CEAs

Pubmed

(economic evaluation[Title/Abstract] OR cost-effectiveness analysis[Title/Abstract] OR CEA[Title/Abstract] OR cost-utility analysis[Title/Abstract] OR CUA[Title/Abstract] OR cost-benefit analysis[Title/Abstract] OR CBA[Title/Abstract]) AND (cancer[Title/Abstract] OR malignant[Title/Abstract] OR carcinoma[Title/Abstract] OR oncology[Title/Abstract] OR lymphoma[Title/Abstract] OR melanoma[Title/Abstract]) NOT (review[Publication Type] OR "clinical trial"[Publication Type] OR "randomized controlled trial"[Publication Type]) AND ("2021/01/01"[Date - Publication] : "2023/12/1"[Date - Publication])

Tufts

(icd_category:("2") OR gbd_category:("409" OR "410")) AND country:"US" AND publication_year:[2003 TO 2022]

Note: the search strategy did not include filtering by disease types from real-world studies. Instead, we ensured that the CEAs included were relevant to the disease types in real-world studies through manual screening. This approach was taken because different tumors are described in various ways, and we were concerned that the search terms might not capture all relevant articles.

© 2025 Zhao M et al. *JAMA Network Open*.

eMethods 3. Distribution of all variables in our study

(1) Proportion of AE Costs for the specific treatment regimen (Scenario Analysis): not normally distributed.
Shapiro-Wilk normality test for real-world absolute AE costs: W = 0.85881, p-value = 0.09307 (normally distributed).
Shapiro-Wilk normality test for CEA absolute AE costs: W = 0.75342, p-value = 0.00594 (not normally distributed)

(2) Absolute AE Costs for the specific treatment regimen: not normally distributed.
Shapiro-Wilk normality test for real-world absolute AE costs: W = 0.71558, p-value = 0.008984 (not normally distributed).
Shapiro-Wilk normality test for CEA absolute AE costs: W = 0.82231, p-value = 0.09241 (normally distributed)

(3) Proportion of AE Costs for the specific treatment regimen (Scenario Analysis): not normally distributed.
Shapiro-Wilk normality test for real-world absolute AE costs: W = 0.82431, p-value = 0.01794 (not normally distributed).
Shapiro-Wilk normality test for CEA absolute AE costs: W = 0.75293, p-value = 0.002868 (not normally distributed)

(4) Absolute change in ICER: not normally distributed.
Shapiro-Wilk normality test: W = 0.5293, p-value = 0.000

(5) Change proportion in ICER: normally distributed.
Shapiro-Wilk normality test for real-world absolute AE costs: W = 0.87841, p-value = 0.111

© 2025 Zhao M et al. *JAMA Network Open*.

eMethods 4. Definitions of jargon and abbreviations

Jargon/Abbreviation	Full Name	Explanation
AE	Adverse Event	An undesirable or harmful effect experienced by a patient during treatment, particularly related to drug therapy.
CEA	Cost-Effectiveness Analysis	A method to evaluate the cost relative to the effectiveness of different interventions, particularly in healthcare, typically expressed as cost per QALY gained.
ICER	Incremental Cost-Effectiveness Ratio	A ratio used to assess the additional cost required to gain one more unit of effectiveness (e.g., one QALY) when comparing two treatments.
ISPOR	International Society for Pharmacoeconomics and Outcomes Research	A global organization aimed at improving decision-making in healthcare through research and education in pharmacoeconomics and health outcomes.
IQR	Interquartile Range	A measure of statistical dispersion, calculated as the range between the 25th and 75th percentiles, representing the spread of the middle 50% of the data.
QALY	Quality-Adjusted Life Year	A measure that combines both the quality and quantity of life lived, often used to assess the value of medical interventions.
RCT	Randomized Controlled Trial	A type of clinical trial where participants are randomly assigned to different treatment groups to study the effects of those treatments.
Adverse Event Cost Proportion		The proportion of the total treatment cost that is attributed to managing adverse events.
Adverse Event Absolute Costs		The total or actual cost incurred due to adverse events, without adjustments or normalization. This represents the raw monetary value associated with managing adverse events.
Claim-Based Data		Data derived from insurance claims, which includes detailed records of healthcare services provided to patients, often used to estimate treatment costs in real-world studies.

© 2025 Zhao M et al. *JAMA Network Open*.

Coefficient of Variation		A measure of the relative variability of a dataset, calculated as the ratio of the standard deviation to the mean, often used to compare cost variability across studies.
Consolidated Health Economic Evaluation Reporting Standards		A set of guidelines focused on standardizing the reporting of health economic evaluations, enhancing clarity and consistency in research publications.
Consumer Price Index		A measure that examines the average change over time in the prices paid by consumers for a basket of goods and services, often used to assess inflation.
Discrete Event Simulation		A method used in health economics to simulate individual patient pathways and the timing of events, such as adverse events or treatment effects, over time.
Dispersion Index		A statistical measure used to assess the spread or concentration of data points within a dataset, often used to describe variability in cost data across different studies.
Dose Reductions and Interruptions		The practice of reducing the dosage or temporarily halting treatment due to adverse events, which can affect both the efficacy and cost of the treatment.
Incremental Cost-Effectiveness Ratio Thresholds		Predefined thresholds (e.g., $100,000/QALY or $150,000/QALY) used to evaluate whether the cost of a treatment is justified by its health benefits.
Markov Model		A mathematical model used in health economics to simulate disease progression over time, where patients move between discrete health states based on defined probabilities.
Medicare		A U.S. federal health insurance program primarily serving people aged 65 and older, and younger individuals with disabilities or specific conditions.
Partitioned Survival Model		A type of health economic model used in oncology, where patients are divided into distinct health states (e.g., progression-free, progressed) based on survival data.
Post-Progression Costs		Costs incurred after the progression of the disease, often associated with managing additional adverse events or administering further treatments.
PRISMA		A set of guidelines designed to improve the reporting of systematic reviews and meta-analyses by ensuring transparency and completeness.
Real-World Data		Data collected from real-life settings (e.g., patient registries, insurance claims) rather than controlled

© 2025 Zhao M et al. *JAMA Network Open*.

		clinical trials, used to assess actual treatment effectiveness and costs.
Relative Cost Differences		A comparison of the costs between different treatments, focusing on the differences in costs related to managing adverse events.
Scenario Analysis		A method used to explore how different assumptions or conditions (such as varying adverse event costs) impact the results of a cost-effectiveness analysis.
Sensitivity Analysis		A method in economic evaluations to test the robustness of the results by varying key parameters (e.g., adverse event costs, treatment effectiveness).
Systematic Review		A comprehensive review of all relevant studies on a particular topic, using a structured and reproducible methodology to identify, assess, and synthesize the evidence.
Treatment-Related Adverse Event		An adverse event that is directly linked to the treatment being administered, rather than other factors.
Wilcoxon Signed-Rank Test		A non-parametric statistical test used to compare two related samples or repeated measurements to assess whether their population mean ranks differ.

© 2025 Zhao M et al. *JAMA Network Open*.

eResults 1. Scenario Analysis Results

In Scenario 1, the analysis by specific drugs was consistent: AE proportion for Real-value was higher than CEA-value (median difference, 8.01%; Interquartile Ranges [IQR], 5.35%~27.13%; p=0.003). For details, see **eFigure 4 and Table 1**.

For Scenario 2, direct Real-cost was significantly greater than CEA-cost (p=0.03) with a median difference of $15,549 (IQR, $12,980~$47,636). For relative AE cost discrepancies, Real-cost was $3,265 higher than CEA-cost for chemotherapy vs. ICI, and $4,178 higher for chemotherapy vs. ICI + chemotherapy. AE cost bias in CEA reversed cost-effectiveness conclusions in 8 of 17 studies at the $100,000/QALY or $150,000/QALY thresholds.

For Scenario 3, direct Real-cost was also significantly higher than CEA-cost with a difference of $15,615 (IQR, $9,340~$47,509; p=0.03). For relative cost discrepancies, Real-cost exceeded CEA-cost by $2,182 for chemotherapy vs. ICI and $4,560 for chemotherapy vs. ICI + chemotherapy. AE cost bias in CEA led to a 33.5% ICER variation, reversing economic conclusions in 50% of studies at the chosen willingness-to-pay thresholds.

© 2025 Zhao M et al. *JAMA Network Open*.

eFigure 1. PRISMA Flowchart of Included Studies: Real-World AE Cost Literature

The initial search yielded 2,010 articles from PubMed (n = 1,278) and Web of Science (n = 732). After removing 382 duplicate studies, 1,628 articles remained for title and abstract screening. From this screening, 1,590 articles were excluded due to the following reasons: they were non-US studies (n = 408), the population was not relevant (n = 315), or they did not report on adverse event (AE) treatment costs or proportion of total treatment costs (n = 887). Consequently, 38 articles were selected for full-text review. During the full-text review, 24 articles were excluded for the following reasons: no AE inclusion criteria (n = 8), missing payer information (n = 2), or unclear patient disease information (n = 14). Additionally, 2 articles were excluded for having follow-up periods shorter than one month (n = 1) or for not specifying cost sources (i.e., payer, patient, or both) (n = 1).

We also employed a snowballing method to ensure no relevant studies were missed. This involved reviewing each included study and using PubMed's "similar articles" feature (which typically generates several dozen to 100 related articles per study) to identify any potential articles that may have been overlooked. Ultimately, 11 studies were included in the final review, with 4 articles reporting absolute AE costs and 9 articles reporting AE costs as a proportion of total treatment costs.

© 2025 Zhao M et al. *JAMA Network Open*.

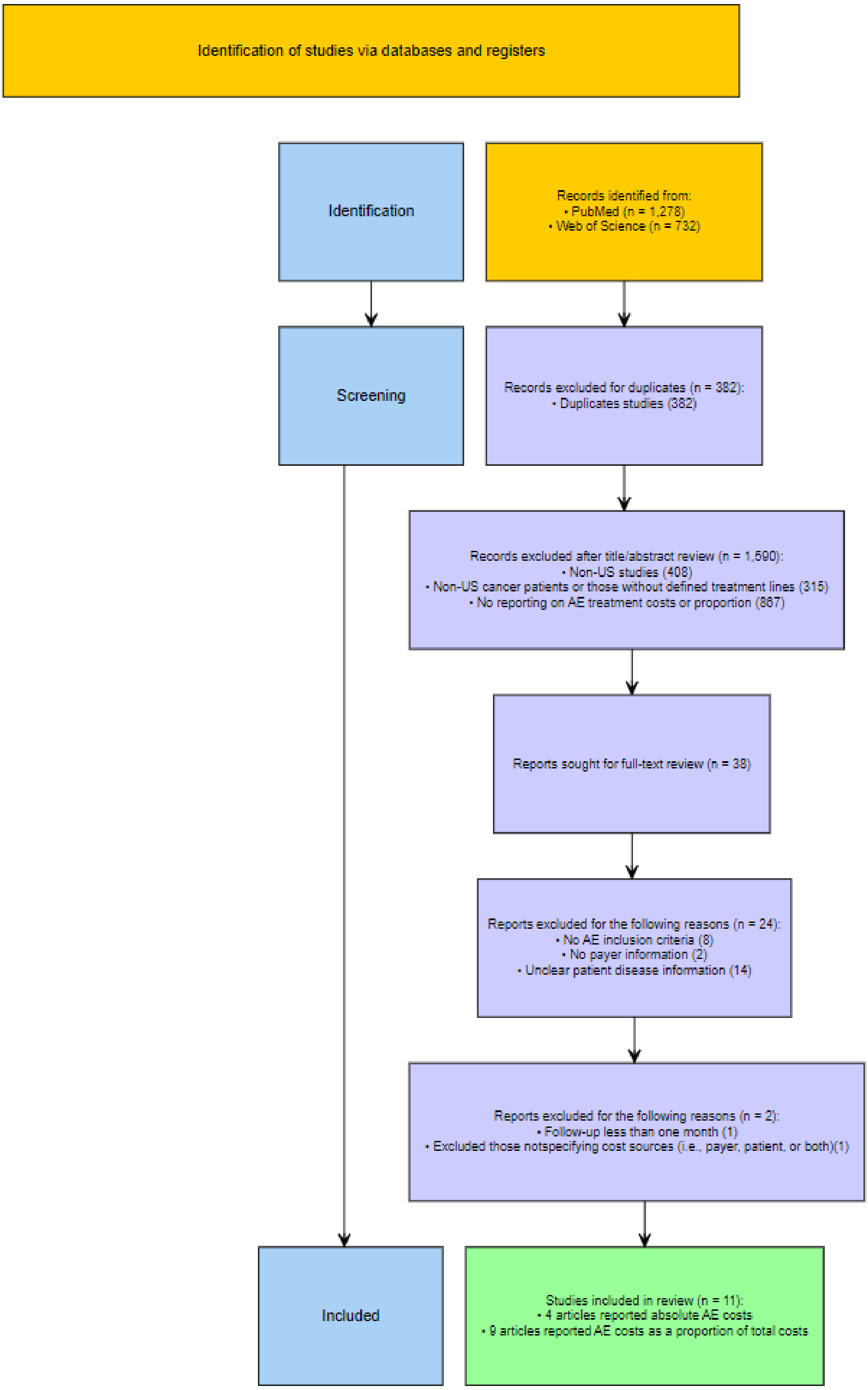

© 2025 Zhao M et al. *JAMA Network Open*.

eFigure 2. PRISMA Flowchart of Included Studies: CEA Literature

Based on tumor types in included real-world AE cost articles, we searched the Tufts database for CEAs from 2003 to 2021 (n=1,437), and PubMed for articles published from 2021 to 2023 (n=3,324), yielding a total of 4,761 articles. After removing 122 duplicates, 4,639 articles remained for screening. Articles were excluded based on title and abstract if they did not meet the following criteria: relevant indications (n=2,854), U.S.-focused studies (n=123), or CEAs (n=1,154). This left 208 articles for full-text review. In the full-text review, 106 CEAs were excluded for the following reasons: non-payer perspectives (n=25), non-pharmacological interventions (n=78), or full text not available (n=5). Ultimately, 102 CEA articles were included in the study evaluating the quantification of AE costs.

© 2025 Zhao M et al. *JAMA Network Open*.

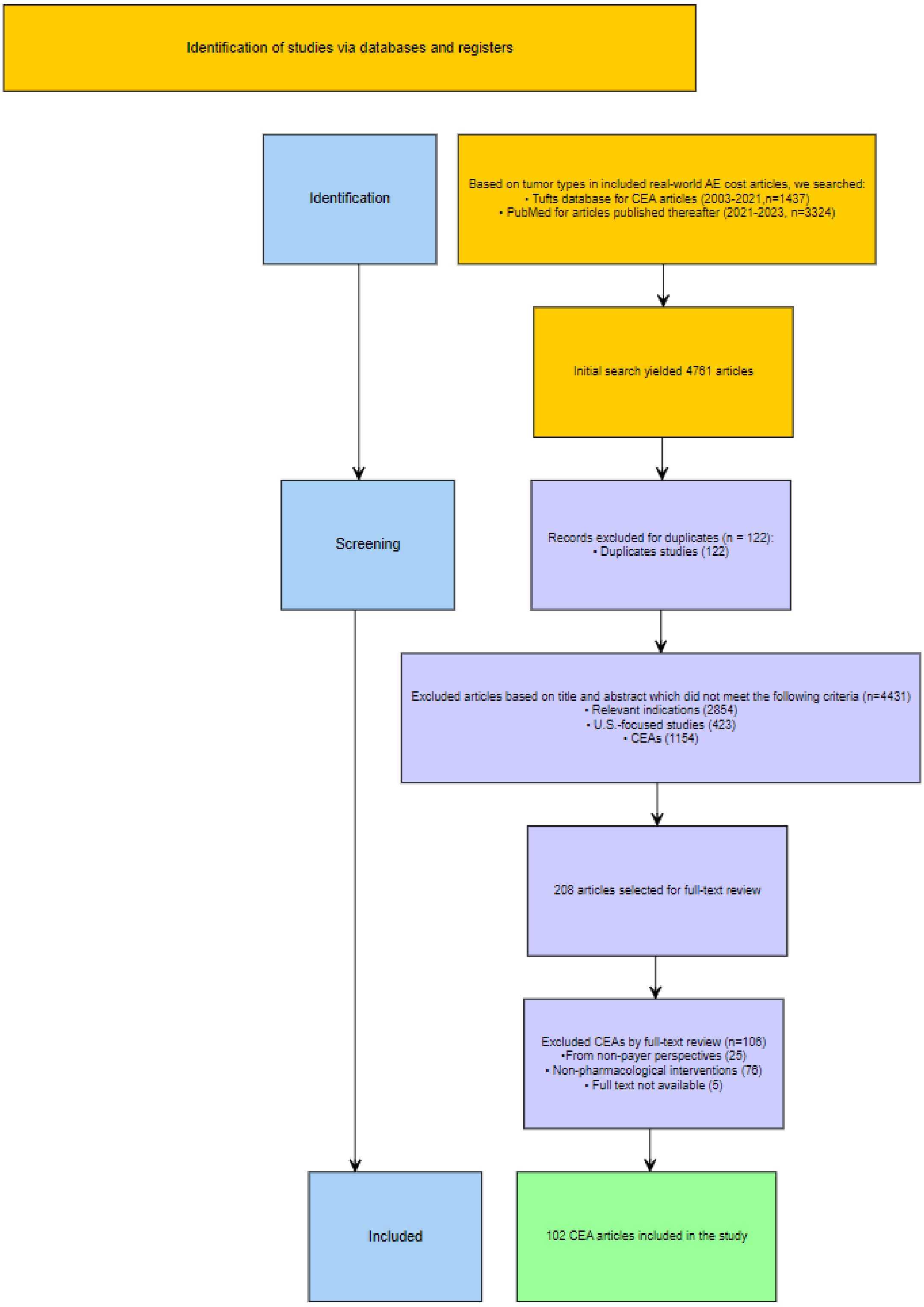

© 2025 Zhao M et al. *JAMA Network Open*.

eTable 1. Key Information of Included 11 Real-World Studies

Pubmed ID	Disease	Treatment	Cost Source	Sample	AE Inclusion Criteria
29885945	1L aNSCLC	Carboplatin,Paclitaxel/Carboplatin, Pemetrexed/Bevacizumab,Carboplatin,Paclitaxel/Carboplatin,Gemcitabine	SEER-Medicare database	5931	AEs with ≥5% incidence as reported in US prescribing information for first-line advanced/metastatic NSCLC drugs. AEs were restricted to grade 3-4, those requiring HCRU (excluding lab investigations), based on CTCAE descriptions and clinical input
32463768	1L aNSCLC	Chemotherapy + Immunotherapy + Combination Approaches	commercial and Medicare Advantage health plan	9712	Those with ≥10% prevalence per drug labels for NCCN-recommended therapies and additional AEs selected by a clinical expert for their severity and/or relevance to immunotherapy
31835965	Non-metastatic prostate cancer	Hormone therapies	IBM Watson MarketScan database,including the MarketScan Commercial Claims and Encounters, Medicare Supplemental and Coordination of Benefits, and Lab databases	532	Patients were classified as having any AE if they had a healthcare claim for a CNS AE, skin rash, or fracture between the index date and end of follow-up. Patients could have CNS AEs, any AEs, both, or neither. Incidence of CNS and any AEs were estimated separately from initiation of index therapy to the first AE diagnosis or end of follow-up
29983370	2L aNSCLC	Pemetrexed/Docetaxel/Carboplatin, Paclitaxel/Gemcit	SEER-Medicare database	4033	Grade 3/4 AEs: Incidence >5%, based on US prescribing information for advanced NSCLC therapies. AEs were restricted to those requiring HCRU (excluding lab investigations), according to CTCAE descriptions and clinical

© 2025 Zhao M et al. *JAMA Network Open*.

		abine			input.
Pubmed ID	Disease	Treatment	Cost Source	Sample	AE Inclusion Criteria
34494389	1L uHCC	TD,ICI,FOLFOX	commercial and Medicare Advantage	414	All immune-mediated AEs were reported, as well all other AEs with a prevalence of ≥5% in the in the total sample of patients.
25085897	1-2L MBC	taxane/capecitabine	PharMetrics Integrated Database	3157	A total 22 AEs associated with chemotherapy were selected
27032967	1-2L MBC	Everolimus/Chemotherapy	Truven Health Analytics MarketScan Commercial and Medicare Supplemental (MarketScan) and IMS Health PharMetrics PlusTM (PharMetrics) databases	3298	See Supplemental Table 1 of the article
25882026	1L aMel	vemurafenib,ipilimumab,dacarbazine,paclitaxel, or temozolomide	IMS PharMetrics Plus claims database	809	Selection of treatment-related AEs: Grade 3/4 events in ≥5% of patients (per package inserts), excluding non-claimable events. Identified by claims with relevant codes
26308231	1L aRCC	pazopanib and sunitinib	MarketScan database	1110	Clinical trial: Reports grade 3/4 AEs with ≥2% incidence in either treatment arm. HCRU components and AEs are detailed in Table 1 and Appendices A and B (online article)
20051811	1L MBC	capecitabine regimens compared with taxane plus anthracycline or other taxane	Thomson Reuters MarketScan	3630	Chemotherapy complications: Anemia, alopecia, asthenia, constipation, cough, dehydration, dermatitis, diarrhea, esophagitis, fever, gastritis, headache, infection, insomnia, mucositis, nausea/vomiting, neutropenia, night sweats, weight loss, and vascular access device complications. Identified by diagnosis codes or treatments

© 2025 Zhao M et al. *JAMA Network Open*.

		regimens			
19738390	Resectable colorectal cancer	Capecitabine or 5-Fluorouracil	Thomson Reuters Market,Scan databases	1,396	Chemotherapy complications: (1) Bone marrow (anemia, neutropenia, thrombocytopenia); (2) Constitutional (asthenia, cough, fever, headache, insomnia, night sweats); (3) GI tract (diarrhea, nausea/vomiting, weight loss, dehydration, constipation, mucositis, esophagitis, gastritis); (4) Infection (including central-line infections); (5) Skin (alopecia, dermatitis); (6) Other (central-line thrombosis, pneumothorax). Identified by diagnosis or treatment. Hand-foot syndrome evaluated using dermatitis codes

Abbreviation: 1L, first-line; 2L, second-line; AE, adverse event; aNSCLC, advanced non-small cell lung cancer; aMel, advanced melanoma; aRCC, advanced renal cell carcinoma; Chemo, chemotherapy; ICI, immune checkpoint inhibitor; MBC, metastatic breast cancer; TD, targeted drug; uHCC, unresectable hepatocellular carcinoma.

© 2025 Zhao M et al. *JAMA Network Open*.

eTable 2. Real-World Studies Reporting Absolute Drug AE Costs (For detailed information on included studies, see eTable 1)

Pubmed ID	Disease	AE	Treatment	Cost/$	Year of Cost	Cost type
32463768	1L aNSCLC	Any grade	Chemotherapy	23,009	2017	Payer+patient
32463768	1L aNSCLC	Any grade	Immune checkpoint inhibitors+Chemotherapy	18,806	2017	Payer+patient
32463768	1L aNSCLC	Any grade	Immune checkpoint inhibitors	16,319	2017	Payer+patient
34494389	1L uHCC	Any grade	Target drugs	66,460	2017	Payer
34494389	1L uHCC	Any grade	Immune checkpoint inhibitors	30,975	2017	Payer
34494389	1L uHCC	Any grade	FOLFOX	61,113	2017	Payer
25085897	1L MBC	Any grade	Capecitabine	4,235	2010	Payer
25085897	1L MBC	Any grade	taxane	17,486	2010	Payer
25085897	2L MBC	Any grade	taxane	21,280	2010	Payer
25085897	2L MBC	Any grade	Capecitabine	22,830	2010	Payer
31835965	Non-metastatic prostate cancer	Any grade	Hormone therapies	11,732/year	2017	Payer

Abbreviation: 1L, first-line; 2L, second-line; AE, adverse event; aNSCLC, advanced non-small cell lung cancer; ICI, immune checkpoint inhibitor; MBC, metastatic breast cancer; uHCC, unresectable hepatocellular carcinoma.

Note: No matching cost-effectiveness analysis was identified for non-metastatic prostate cancer

© 2025 Zhao M et al. *JAMA Network Open*.

eTable 3. Real-World Studies Reporting Proportion of Drug AE Costs Relative to Total Medical Costs (For detailed information on included studies, see eTable 1)

Pubmed ID	Disease	AE	Treatment	AE cost proportion
25882026	1L aMel	Any grade	Vemurafenib	6.40%
25882026	1L aMel	Any grade	Ipilimumab	4.00%
25882026	1L aMel	Any grade	Dacarbazine	36.90%
25882026	1L aMel	Any grade	Temozolomide	9.20%
25882026	1L aMel	Any grade	Paclitaxel	30.40%
34494389	1L uHCC	Any grade	Target drugs	38.13%
34494389	1L uHCC	Any grade	Immune checkpoint inhibitors	27.88%
34494389	1L uHCC	Any grade	FOLFOX	34.36%
26308231	1L uRCC	Any grade	Sunitinib	16.51%
26308231	1L uRCC	Any grade	Pazopanib	10.09%
29885945	1L aNSCLC	Grade 3-4	Carboplatin, Paclitaxel	10.18%
29885945	1L aNSCLC	Grade 3-4	Carboplatin, Pemetrexed	6.98%
29885945	1L aNSCLC	Grade 3-4	Bevacizumab, Carboplatin, Paclitaxel	9.32%
29885945	1L aNSCLC	Grade 3-4	Carboplatin, Gemcitabine	9.84%
29983370	2L aNSCLC	Any grade	Pemetrexed	9.52%
29983370	2L aNSCLC	Any grade	Docetaxel	11.27%
29983370	2L aNSCLC	Any grade	Carboplatin, Paclitaxel	9.95%
29983370	2L aNSCLC	Any grade	Gemcitabine	12.68%

© 2025 Zhao M et al. *JAMA Network Open*.

20051811	1L MBC	Any grade	Capecitabine	13.31%
20051811	1L MBC	Any grade	Taxane+Anthracycline	43.06%
Pubmed ID	Disease	AE	Treatment	AE cost proportion
20051811	1L MBC	Any grade	Taxane	31.88%
27032967	1L MBC	Any grade	Everolimus	31.54%
27032967	1L MBC	Any grade	Chemotherapy	37.57%
25085897	1L MBC	Any grade	Taxane	26.80%
25085897	1L MBC	Any grade	Capecitabine	7.01%
27032967	2L MBC	Any grade	Everolimus	24.50%
27032967	2L MBC	Any grade	Taxane	44.05%
19738390	Resectable CRC	Any grade	Chemotherapy	8.57%
19738390	Resectable CRC	Any grade	Chemotherapy	18.53%

Abbreviation: 1L, first-line; 2L, second-line; AE, adverse event; aNSCLC, advanced non-small cell lung cancer; aMel, advanced melanoma; aRCC, advanced renal cell carcinoma; CRC, colorectal cancer; ICI, immune checkpoint inhibitor; MBC, metastatic breast cancer; MUC, metastatic urothelial cancer; TD, targeted drug; uHCC, unresectable hepatocellular carcinoma.

Note: No matching cost-effectiveness analysis was identified for resectable colorectal cancer

© 2025 Zhao M et al. *JAMA Network Open*.

eTable 4. Key Information from Included 102 CEA Studies

Pubmed ID	Disease	Post-Progression AE Costs	Dose Modification	AE incidence sources and whether AEs were missed under the inclusion criteria			AEs inclusion criteria					Sources of Unit AE Costs
				AE incidence sources	Whether AEs correctly included	Related AEs	Grade	Calculation rules	Within-group rate	Between-group difference in rate	Type	
33625508	1L uHCC	No	No	IMbrave150	No, missed AEs	rash, decreased appetite	Grade 3+	Occur in the first cycle	Any rate	Any rate	Treatment-related	1.Claim study;2.RED BOOK;3.Modeling study
36160459	1L MBC	No	Yes	DESTINY-Breast03	No, missed AEs	Aspartate aminotransferase increased	Grade 3-4	Occur in the first cycle	Over 5% of patients and had significantly different rates between treatment	Notably rate	Treatment-related	1.Claim study;2.Modeling study
33668100	1L uHCC	No	Yes	IMbrave150	Yes	NA	Grade 3-4	Occur in the first cycle	Over 3%	Any rate	Treatment-related	1.Claim study;2.Modeling study
33556230	1L uHCC	No	No	IMbrave150	No, missed AEs	Fatigue and others	Grade 3+	Occur in the first cycle	Any rate	Any rate	Treatment-related	NA

© 2025 Zhao M et al. *JAMA Network Open*.

Pubmed ID	Disease	Post-Progression AE Costs	Dose Modification	AE incidence sources and whether AEs were missed under the inclusion criteria			AEs inclusion criteria					Sources of Unit AE Costs
				AE incidence sources	Whether AEs correctly included	Related AEs	Grade	Calculation rules	Within-group rate	Between-group difference in rate	Type	
34019245	1L MUC	No	No	Ignore AEs	NA	NA	NA	Ignore AEs	NA	NA	NA	NA
21039835	1L uHCC	No	No	SHARP	NA	NA	Grade 3-4	Cyclic probability	Over 10%	Any rate	Treatment-related	US expert opinion
30458884	1L aRCC	No	No	CheckMate 214	No, missed AEs	Stomatitis and others	Grade 3-4	Occur in the first cycle	Any rate	Notably rate	Treatment-related	1.Modeling study;2.Claim study;3.prospective or retrospective studies
32522057	1L MBC	No	No	IMpassion130	No, wrong incidence, missed AEs	Neutrophil count decrease and others	Grade 3+	Occur in the first cycle	Any rate	Any rate	Treatment-related	data not found in the referenced article
32697113	1L aRCC	No	No	KEYNOTE-426/COMPARZ	NA	NA	Grade 3+	Occur in the first cycle	Over 5%	Any rate	All-cause	Claim study
28472324	1L MBC	Yes	No	PALOMA-1/TRIO-18	Yes	NA	Grade 3+	Occur in the first cycle	Any rate	Any rate	All-cause	1.Longitudinal discharge database;2.Claim study;3.Modeling study

© 2025 Zhao M et al. *JAMA Network Open*.

28221865	1L aMel	Yes	Yes	Multiple RCTs	No, wrong incidence, missed AEs	Diarrhea and others	Any grade	Cyclic probability	NA	NA	NA	Claim study
Pubmed ID	Disease	Post-Progression AE Costs	Dose Modification	AE incidence sources and whether AEs were missed under the inclusion criteria			AEs inclusion criteria					Sources of Unit AE Costs
				AE incidence sources	Whether AEs correctly included	Related AEs	Grade	Calculation rules	Within-group rate	Between-group difference in rate	Type	
33417490	1L uHCC	No	No	NA	NA	NA	Grade 3+	Occur in the first cycle	Any rate	Any rate	NA	1.Claim study;2.Modeling study
37085377	3L MBC	No	No	DESTINY-Breast04	Yes	NA	Grade 3+	Occur in the first cycle	Any rate	Any rate	Treatment-related	Modeling study
32457618	1L aRCC	No	No	JAVELIN Renal 101	No, wrong incidence, missed AEs	Neutrophil count decrease and others	Grade 3+	Occur in the first cycle	Over 5%	Any rate	All-cause	Modeling study
37271697	1L aRCC	No	No	CheckMate 9ER and CABOSUN	NA	NA	Grade 3+	Occur in the first cycle	NA	NA	NA	Modeling study
33825837	1L uHCC	No	No	IMbrave150	No, missed AEs	Aspartate aminotransferase increase,blood bilirubin increase	Grade 3+	Occur in the first cycle	Over 2%	Any rate	Treatment-related	Modeling study
34762112	1L aMel	No	No	IMspire150	Yes	NA	Any	Occur in the	Any rate	Any rate	Treatment	1.Claim

© 2025 Zhao M et al. *JAMA Network Open*.

							grade	first cycle			-related	study;2.Modeling study
Pubmed ID	Disease	Post-Progression AE Costs	Dose Modification	AE incidence sources and whether AEs were missed under the inclusion criteria			AEs inclusion criteria					Sources of Unit AE Costs
				AE incidence sources	Whether AEs correctly included	Related AEs	Grade	Calculation rules	Within-group rate	Between-group difference in rate	Type	
35924662	1L MUC	No	No	IMvigor130	No, wrong incidence, missed AEs	Thrombocytopenia	Grade 3+	Occur in the first cycle	Over 5%	Any rate	Treatment -related	Modeling study
30563395	1L aMel	Yes	No	Multiple RCTs	NA	NA	Grade 3-4	Occur in the first cycle	NA	NA	Treatment -related	Claim study
34991104	1L aRCC	No	No	CheckMate 214 and KEYNOTE-426	Yes	NA	Grade 3+	Occur in the first cycle	Over 1%	Any rate	NA	1.Clinical Laboratory Fee Schedule;2.Claim study
19117341	1L MBC	No	No	NA	NA	NA	Grade 3+	Occur in the first cycle,only consider diarrhea and cardiotoxicity event	Any rate	Any rate	NA	Retrospective cohort database analysis
36599117	1L aRCC	Yes	No	Javelin Renal	Yes	NA	Grade	Occur in the	Over 5%	Any rate	Treatment	1.Modelling

© 2025 Zhao M et al. *JAMA Network Open*.

				101			3+	first cycle			-related	study;2.Claim study
Pubmed ID	Disease	Post-Progression AE Costs	Dose Modification	AE incidence sources and whether AEs were missed under the inclusion criteria			AEs inclusion criteria					Sources of Unit AE Costs
				AE incidence sources	Whether AEs correctly included	Related AEs	Grade	Calculation rules	Within-group rate	Between-group difference in rate	Type	
34664194	1L aRCC	No	No	CheckMate 9ER	No, missed AEs	Multiple AEs	Grade 3+	Occur in the first cycle	Any rate	Any rate	Treatment -related	Modelling study
36653848	1L aMel	No	Yes	NA	NA	NA	NA	Directly citing total drug-related AE costs from CEAs	NA	NA	NA	Modeling study
36006606	1L aMel	No	Yes	Multiple RCTs	Yes	NA	Grade 3+	Cyclic probability	NA	NA	Treatment -related	Claim study
18711190	1L aRCC	No	Yes	Multiple RCTs	NA	NA	Grade 3+	Cyclic probability	NA	NA	Treatment -related	1.Claim study;3.Red book
30477000	1L aMel	No	No	NCT01740297	No, missed AEs	Rash,Pyrexia,Influenza-like illness	Grade 3+	Cyclic probability	NA	NA	NA	Modelling study
30789633	1L aRCC	No	No	CheckMate 214	Yes	NA	Grade 3-4	Occur in the first cycle	Any rate	different rates	Treatment -related	1.Modeling study;2.Claim study
36529626	1L aRCC	No	No	CLEAR	No, additional AEs were	amylase increased,asthenia	Grade 3+	Occur in the first cycle	Over 5%	Any rate	NA	Modeling study

© 2025 Zhao M et al. *JAMA Network Open*.

Pubmed ID	Disease	Post-Progression AE Costs	Dose Modification	AE incidence sources and whether AEs were missed under the inclusion criteria			AEs inclusion criteria					Sources of Unit AE Costs
					considered							
				AE incidence sources	Whether AEs correctly included	Related AEs	Grade	Calculation rules	Within-group rate	Between-group difference in rate	Type	
26171248	1L aMel	No	No	Multiple RCTs	No, missed AEs	Fatigue and others	Any grade	Occur in the first cycle	Over 5%	Any rate	NA	1.Claim study;2.Red book;3.Retrospective cohort database analysis
36282933	1L MBC	No	No	MONALEESA-3	No, missed AEs	Renal toxicity and others	Grade 3+	Occur in the first cycle	Over 2%	Over 2%	NA	1.Claim study;2.Modeling study;3.Retrospective cohort database analysis
34966275	1-2L aRCC	No	No	NA	NA	NA	NA	Directly citing total drug-related AE costs from disease burden research	NA	NA	NA	Modelling Study
36639851	1L aMel	No	No	Multiple RCTs	Yes	NA	Grade	Occur in the	Any rate	Any rate	NA	1.Cost Systematic

© 2025 Zhao M et al. *JAMA Network Open*.

							3+	first cycle				Review;2.Modeling study
Pubmed ID	Disease	Post-Progression AE Costs	Dose Modification	AE incidence sources and whether AEs were missed under the inclusion criteria			AEs inclusion criteria					Sources of Unit AE Costs
				AE incidence sources	Whether AEs correctly included	Related AEs	Grade	Calculation rules	Within-group rate	Between-group difference in rate	Type	
28301684	2L aRCC	No	No	CheckMate 025	NA	NA	Grade 3-4	Occur in the first cycle	Any rate	Over 4%	NA	Modeling study
14693319	1L MBC	Yes	No	Arimidex	No, wrong incidence, missed AEs	Asthenia and others	Any grade	Occur in the first cycle	Any rate	Any rate	NA	NA
32697281	1L aMel	No	No	NA	NA	NA	Grade 3+	Cyclic probability	NA	NA	NA	1.Red Book;2.Modeling study
21265994	1L aRCC	No	No	Multiple RCTs and label information	NA	NA	Grade 3-4	Occur in the first cycle	Any rate	Any rate	NA	1.Claim study;2.Red book
22200867	1L MBC	No	No	NA	NA	NA	NA	Ignore AEs	NA	NA	NA	NA
21914503	1L aNSCLC	No	No	ECOG 4599	No, missed AEs	Hyponatremia	Grade 3-4	Occur in the first cycle	Any rate	Any rate	NA	Modelling study
32826180	1L MUC	No	No	Multiple RCTs	No, missed AEs	Fatigue	Grade 3-4, grade 2+	Occur in the first cycle	Over 5%	Any rate	All-cause	Claim study

© 2025 Zhao M et al. *JAMA Network Open*.

							diarrhea and all-grade febrile neutropenia					
Pubmed ID	Disease	Post-Progression AE Costs	Dose Modification	AE incidence sources and whether AEs were missed under the inclusion criteria			AEs inclusion criteria					Sources of Unit AE Costs
				AE incidence sources	Whether AEs correctly included	Related AEs	Grade	Calculation rules	Within-group rate	Between-group difference in rate	Type	
36780838	2L MBC	No	No	ASCENT	No, missed AEs	Infections,Vomiting and others	Grade 3-4	Occur in the first cycle	Any rate	Any rate	Treatment-related	Claim study
34512315	1L aNSCLC	No	No	CheckMate 227	No, missed AEs	Decreased appetite	Any grade	NA	NA	NA	Treatment-related	Modeling study
36071854	1L MUC	No	No	IMvigor130	No, missed AEs	Platelet count decreased,Urinary tract infection and others	Grade 3-4	Occur in the first cycle	Any rate	Any rate	Treatment-related	Claim study
37537015	3L MBC	No	No	DESTINY-Breast04	NA	NA	Grade 3+	Occur in the first cycle	Over 5%	Over 2%	Treatment-related	Modeling study
36653947	1L aNSCLC	No	No	DESTINY-Breast04	Yes	NA	Any grade	Occur in the first cycle	NA	NA	NA	Claim study
30268469	1L	No	No	KEYNOTE-	No, missed	Pyrexia,pruritus,ras	Grade	Occur in the	Over 5%	Any rate	All-cause	Claim study

© 2025 Zhao M et al. *JAMA Network Open*.

	aNSCLC			024	AEs	h and others	3+	first cycle				
Pubmed ID	Disease	Post-Progression AE Costs	Dose Modification	AE incidence sources and whether AEs were missed under the inclusion criteria			AEs inclusion criteria					Sources of Unit AE Costs
				AE incidence sources	Whether AEs correctly included	Related AEs	Grade	Calculation rules	Within-group rate	Between-group difference in rate	Type	
32426048	1L MBC	No	No	IMpassion130	No, wrong incidence, missed AEs	Multiple AEs	Grade 3+	Occur in the first cycle	Any rate	Any rate	All-cause	Claim study
35957949	2L MBC	No	No	MONARCH 2	No, missed AEs	Multiple AEs	Grade 3-4	Occur in the first cycle	Any rate	Any rate	Treatment-related	NA
31610828	1L NSCLC	No	No	KEYNOTE-042	No, missed AEs	Leucopenia and others	serious AEs	Occur in the first cycle	Over 1%	Any rate	Treatment-related	Modeling study
37386633	3L MBC	No	No	NA	NA	NA	Grade 3+	Directly citing total drug-related AE costs from disease burden research	NA	NA	NA	Claim study
34290602	1L aNSCLC	No	No	NA	NA	NA	Grade 3+	Directly citing total drug-related AE costs	NA	NA	NA	Modeling study

© 2025 Zhao M et al. *JAMA Network Open*.

								from disease burden research				
Pubmed ID	Disease	Post-Progression AE Costs	Dose Modification	AE incidence sources and whether AEs were missed under the inclusion criteria			AEs inclusion criteria					Sources of Unit AE Costs
				AE incidence sources	Whether AEs correctly included	Related AEs	Grade	Calculation rules	Within-group rate	Between-group difference in rate	Type	
34778047	3L MBC	No	No	ASCENT	No, missed AEs	fatigue and decreased appetite	Grade 3+	Occur in the first cycle	Any rate	Any rate	Treatment-related	1.Claim study;2.Red book;3.Retrospective cohort database analysis
18295368	2L NSCLC	No	No	Multiple RCTs	NA	NA	Grade 3+	Occur in the first cycle	Over 5%	Any rate	NA	Claim study
36532255	3L MBC	No	No	DESTINY-Breast04	No, missed AEs	increased amino transaminase	Grade 3-4	Occur in the first cycle	Any rate	Any rate	Treatment-related	1.Claim study;2.Modeling study;3.Retrospective database analysis
35796042	1L MBC	No	Yes	KEYNOTE-355	Yes	NA	Grade 3-4	Occur in the first cycle	Over 5%	Any rate	All-cause	1.Claim study;2.Modeling study;3.Retrospective database

© 2025 Zhao M et al. *JAMA Network Open*.

												analysis
33645243	1L MBC	No	No	MONALEES A-7	Yes	NA	Grade 3-4	Directly citing total drug-related AE costs from literature	NA	NA	NA	Claim study
26048087	2L MBC	Yes	Yes	BOLERO-2	NA	NA	Grade 3+	Occur in the first cycle	Over 5%	Any rate	Treatment -related	Claim study
Pubmed ID	Disease	Post-Progression AE Costs	Dose Modification	AE incidence sources and whether AEs were missed under the inclusion criteria			AEs inclusion criteria					Sources of Unit AE Costs
				AE incidence sources	Whether AEs correctly included	Related AEs	Grade	Calculation rules	Within-group rate	Between-group difference in rate	Type	
31853795	1L MBC	No	No	S0226	Yes	NA	Serious AE	Cyclic probability	NA	NA	NA	1.Cost Systematic Review;2.Retrospective cohort database analysis
27654970	2L MBC	Yes	No	Multiple RCTs	NA	NA	Grade 3+	Occur in the first cycle	Over 5%	Any rate	Treatment -related	Modeling study
33938936	1L aNSCLC	No	No	CheckMate 227	Yes	NA	Grade 3+	Occur in the first cycle	Any rate	Any rate	Treatment -related	1.Cost Systematic Review;2.Modeling study
34026637	1L MBC	No	No	MONALEES	No, wrong	Multiple AEs	Two	Occur in the	Any rate	Any rate	Treatment	1.Cost Systematic

© 2025 Zhao M et al. *JAMA Network Open*.

				A-7	incidence		major grade 3-4 AEs	first cycle			-related	Review;2.Modeling study
36920662	1L MBC	No	Yes	PALOMA-2	Yes	NA	Severe neutropenia	Occur in the first cycle	Any rate	Any rate	All-cause	Retrospective cohort database analysis
Pubmed ID	Disease	Post-Progression AE Costs	Dose Modification	AE incidence sources and whether AEs were missed under the inclusion criteria			AEs inclusion criteria					Sources of Unit AE Costs
				AE incidence sources	Whether AEs correctly included	Related AEs	Grade	Calculation rules	Within-group rate	Between-group difference in rate	Type	
34150798	2L MBC	No	No	PALOMA-3	No, wrong incidence	Multiple AEs	Grade 3+	Occur in the first cycle	Over 5%	Any rate	All-cause	Retrospective database analysis
31738117	1L aNSCLC	No	No	KEYNOTE-042	No, wrong incidence	Multiple AEs	Grade 3+	Occur in the first cycle	Over 5%	Any rate	Treatment-related	1.Claim study;2.Cost Systematic Review
31104762	2L MBC	No	No	PALOMA-3	No, wrong incidence	Multiple AEs	Serious AEs	Occur in the first cycle	Over 5%	Over 4%	Treatment-related	Claim study
31831534	1L aNSCLC	No	No	Keynote-189	NA	NA	Grade 3+	Occur in the first cycle	Over 5%	Any rate	NA	Retrospective cohort database analysis
34100243	1L aNSCLC	No	No	CheckMate 9LA	No, missed AEs	Multiple AEs	Serious AEs	Occur in the first cycle	Over 5%	Over 2%	Treatment-related	Modeling study
36327624	2L MBC	No	No	DESTINYBre	NA	NA	Grade	Occur in the	Over 5%	Any rate	NA	Modeling study

© 2025 Zhao M et al. *JAMA Network Open*.

				ast03			3+	first cycle				
29852038	1L aNSCLC	No	No	NA	NA	NA	NA	NA	NA	NA	NA	Modeling study
34093040	1L aNSCLC	No	No	ARCHER 1050	No, missed AEs	Multiple AEs	Grade 3+	Occur in the first cycle	Any rate	Any rate	All-cause	NA
Pubmed ID	Disease	Post-Progression AE Costs	Dose Modification	AE incidence sources and whether AEs were missed under the inclusion criteria			AEs inclusion criteria					Sources of Unit AE Costs
				AE incidence sources	Whether AEs correctly included	Related AEs	Grade	Calculation rules	Within-group rate	Between-group difference in rate	Type	
34734033	1L aNSCLC	No	No	IMpower110	Yes	NA	Grade 3+	Occur in the first cycle	Over 5%	Any rate	NA	Modeling study
33209600	1L aNSCLC	Yes	No	KEYNOTE-189 and KEYNOTE-407	Yes	NA	Grade 3+	Occur in the first cycle	Any rate	Any rate	All-cause	Modeling study
30649973	1L aNSCLC	No	Yes	KEYNOTE-407	No, wrong incidence	NA	Grade 3+	Occur in the first cycle	Over 5%	Any rate	All-cause	Modeling study
31287562	1L aNSCLC	No	No	IMpower150	No, wrong incidence	NA	Any grade	NA	NA	NA	Treatment-related	Medicare
31553470	1L aNSCLC	Yes	No	Multiple RCTs	NA	NA	Grade 3+	Occur in the first cycle	Any rate	Any rate	NA	1.Modeling study;2.Claim study
26122345	1L aNSCLC	Yes	No	Multiple RCTs	NA	NA	Grade 3-4	Directly citing total	NA	NA	NA	Claim study

© 2025 Zhao M et al. *JAMA Network Open*.

								drug-related AE costs from literature				
32193809	1L aNSCLC	Yes	No	IMpower130	No, missed AEs	Leukopenia	Grade 3-4	Occur in the first cycle	Any rate	Notably difference	Treatment-related	Claim study
Pubmed ID	Disease	Post-Progression AE Costs	Dose Modification	AE incidence sources and whether AEs were missed under the inclusion criteria			AEs inclusion criteria					Sources of Unit AE Costs
				AE incidence sources	Whether AEs correctly included	Related AEs	Grade	Calculation rules	Within-group rate	Between-group difference in rate	Type	
36503033	1-2L NSCLC	No	Yes	Medication guide	No, missed AEs	Constipation and others	Grade 3-4	Occur in the first cycle	Any rate	Any rate	NA	Modeling study
37854153	1L aNSCLC	Yes	No	Multiple RCTs	No, wrong incidence, missed AEs	Leukopenia and others	Grade 3+	Occur in the first cycle	Over 4%	Any rate	Treatment-related	Claim study
35659172	1L aNSCLC	No	No	CheckMate 227	No, wrong incidence	Neutropenia	Grade 3+	Occur in the first cycle	Over 5%	Any rate	Treatment-related	Modeling study
34736841	1L aNSCLC	No	No	EMPOWER-Lung 1	No, missed AEs	Nausea and others	Grade 3+	Occur in the first cycle	Over 5%	Any rate	Treatment-related	NA
31655368	1L aNSCLC	No	Yes	KEYNOTE-042	NA	NA	Grade 3+	Occur in the first cycle	Over 5%	Any rate	Treatment-related	RED BOOK
35094793	1L	No	No	Multiple RCTs	No, missed	Multiple AEs	Grade	Occur in the	Over 5%	Any rate	NA	Modeling study

© 2025 Zhao M et al. *JAMA Network Open*.

	aNSCLC				AEs		3+	first cycle				
34268373	1L aNSCLC	No	No	Multiple RCTs	NA	NA	Grade 3+	Cyclic probability	NA	NA	NA	Claim study
35658806	1L aNSCLC	No	No	CheckMate 9LA	No, missed AEs	Decreased neutrophil count	Grade 3+	Occur in the first cycle	Over 5%	NA	Treatment -related	Cost Systematic Review
Pubmed ID	Disease	Post-Progression AE Costs	Dose Modification	AE incidence sources and whether AEs were missed under the inclusion criteria			AEs inclusion criteria					Sources of Unit AE Costs
				AE incidence sources	Whether AEs correctly included	Related AEs	Grade	Calculation rules	Within-group rate	Between-group difference in rate	Type	
35658806	1L aNSCLC	No	No	CheckMate 227	NA	NA	Grade 3+	Occur in the first cycle	Over 5%	NA	Treatment -related	Claim study
31945265	1L aNSCLC	No	No	KEYNOTE-189	Yes	NA	Serious AEs	Cyclic probability	Top three	NA	NA	Claim study
31995619	1L aNSCLC	Yes	No	Multiple RCTs	NA	NA	Grade 3+	NA	NA	NA	NA	Claim study
32524512	1L aNSCLC	No	No	IMpower130	No, missed AEs	Multiple AEs	Grade 3+	Occur in the first cycle	Over 1%	Any rate	Treatment -related	Claim study
32811247	1L aNSCLC	No	No	CheckMate 227	No, wrong incidence, missed AEs	Decreased appetite and others	Grade 3-4	Occur in the first cycle	Most common	Any rate	Treatment -related	1.Modeling study;2.Claim study
33014826	1L aNSCLC	No	No	CheckMate 227	No, wrong incidence	Multiple AEs	Grade 3-4	Occur in the first cycle	Over 1%		Treatment -related	Modeling study
33732316	1-2L	Yes	No	NA	NA	NA	NA	Cyclic	NA	NA	NA	NA

© 2025 Zhao M et al. *JAMA Network Open*.

	NSCLC							probability				
30710066	1L aRCC	No	No	CheckMate 214	No, missed AEs	Thrombocytopenia and others	Grade 3-4	Occur in the first cycle	Any rate	Over 4%	Treatment -related	NA
31992108	1L aRCC	Yes	No	Multiple RCTs	Yes	NA	Grade 3-4	Occur in the first cycle	Any rate	Any rate	Treatment -related	Claim study
Pubmed ID	Disease	Post-Progression AE Costs	Dose Modification	AE incidence sources and whether AEs were missed under the inclusion criteria			AEs inclusion criteria					Sources of Unit AE Costs
				AE incidence sources	Whether AEs correctly included	Related AEs	Grade	Calculation rules	Within-group rate	Between-group difference in rate	Type	
32918790	1L aRCC	No	No	KEYNOTE-426	No, wrong incidence, missed AEs	Fatigue and others	Grade 3-4	Occur in the first cycle	Any rate	Notably difference	NA	Modelling study
23057750	2L aMel	No	No	MDX010-20 trial	NA		Grade 3-4	Occur in the first cycle	Any rate	Any rate	NA	Claim study
25198196	1L aMel	No	No	NA	NA	NA	Neutropenia	NA	NA	NA	NA	Claim study
28530525	1L aMel	No	No	CheckMate-067	No, wrong incidence	rash	Any grade	Occur in the first cycle	Over 5%	Any rate	Treatment -related	Claim study
28125365	1L aMel	No	No	KEYNOTE-006	NA	NA	Grade 3-4	Occur in the first cycle	Over 2%	Any rate	Treatment -related	Claim study
34858820	1L aMel	No	No	NA	NA	NA	Grade 3+	Ignore AEs	NA	NA	Treatment -related	NA
30847728	1L MBC	No	No	Multiple RCTs	Yes	NA	Neutrop	NA	NA	NA	NA	Claim study

© 2025 Zhao M et al. *JAMA Network Open*.

							enia					
33364838	1L MBC	No	Yes	MONALEESA-3	No, missed AEs	Multiple AEs	Grade 3-4	Occur in the first cycle	Any rate	Notably difference	All-cause	1.Claim study;2.Retrospective cohort database analysis

NA, not available.

Abbreviation: 1L, first-line; 2L, second-line; AE, adverse event; aNSCLC, advanced non-small cell lung cancer; aMel, advanced melanoma; aRCC, advanced renal cell carcinoma; CEA, cost-effectiveness analysis; Chemo, chemotherapy; ICI, immune checkpoint inhibitor; MBC, metastatic breast cancer; MUC, metastatic urothelial cancer; TD, targeted drugs.

© 2025 Zhao M et al. *JAMA Network Open*.

eTable 5. Summary of Unit Costs for Grade 1-2 AEs in CEAs

PMID	Unit Cost/$*	Disease	Source of Cost	Year of Cost
Anemia				
36653947	4353	1L aNSCLC	Claim study	2021
30268469	4353	1L aNSCLC	Claim study	2018
Constipation				
26171248	5.81	1L aMel	1.Claim study;2.Red book;3.Retrospective cohort database analysis	2013
36653947	3265	1L aNSCLC	Claim study	2021
30268469	3265	1L aNSCLC	Claim study	2018
Fatigue				
36653947	733	1L aNSCLC	Claim study	2021
Hyperkeratosis				
26171248	126.66	1L aMel	1.Claim study;2.Red book;3.Retrospective cohort database analysis	2013
Hyperthyroidism				
36653947	2255	1L aNSCLC	Claim study	2021
30268469	2255	1L aNSCLC	Claim study	2018
Keratoacanthoma				
26171248	113.67	1L aMel	1.Claim study;2.Red book;3.Retrospective cohort database analysis	2013
Nausea				
26171248	84.66	1L aMel	1.Claim study;2.Red book;3.Retrospective cohort database analysis	2013
36653947	1965	1L aNSCLC	Claim study	2021
30268469	1965	1L aNSCLC	Claim study	2018
Neutropenia				
36653947	5321	1L aNSCLC	Claim study	2021
30268469	5321	1L aNSCLC	Claim study	2018
Leukopenia				
26171248	0	1L aMel	1.Claim study;2.Red book;3.Retrospective cohort database analysis	2013
Palmar-plantar erythrodysesthesia/Hand-foot syndrome				
26171248	113.67	1L aMel	1.Claim study;2.Red book;3.Retrospective cohort database analysis	2013
Platelet count decrease				
30268469	0	1L aNSCLC	Claim study	2018
Pneumonitis				
36653947	9941	1L aNSCLC	Claim study	2021
30268469	9941	1L aNSCLC	Claim study	2018
Pruritus				
36653947	1184	1L aNSCLC	Claim study	2021
Rash				
36653947	940	1L aNSCLC	Claim study	2021

© 2025 Zhao M et al. *JAMA Network Open*.

PMID	Unit Cost/$	Disease	Source of Cost	Year of Cost
Squamous-cell carcinoma				
26171248	1595	1L aMel	1.Claim study;2.Red book;3.Retrospective cohort database analysis	2013
Vomiting				
36653947	895	1L aNSCLC	Claim study	2021
30268469	895	1L aNSCLC	Claim study	2018
Weight decreased/Anorexia				
36653947	3700	1L aNSCLC	Claim study	2021
White-cell count decreased				
30268469	0	1L aNSCLC	Claim study	2018

Abbreviation: 1L, first-line; 2L, second-line; AE, adverse event; aNSCLC, advanced non-small cell lung cancer; aMel, advanced melanoma; aRCC, advanced renal cell carcinoma; CEA, cost-effectiveness analysis; Chemo, chemotherapy; ICI, immune checkpoint inhibitor; MBC, metastatic breast cancer; MUC, metastatic urothelial cancer; TD, targeted drug; uHCC, unresectable hepatocellular carcinoma.

* Without CPI adjusted.

© 2025 Zhao M et al. *JAMA Network Open*.

eTable 6. Summary of Unit Costs for Grade 3+ AEs in CEAs

PMID	Unit Cost/$*	Disease	Source of Cost	Year of Cost
Abdominal pain				
33825837	2534	1L uHCC	Modeling study	2020
34991104	7457	1L aRCC	1.Clinical Laboratory Fee Schedule;2.Claim study	2019
36006606	2511	1L aMel	Claim study	2020
31992108	6538	1L aRCC	Claim study	2018
Abnormal lipase level				
34762112	6094	1L aMel	1.Claim study;2.Modeling study	2020
34991104	8181	1L aRCC	1.Clinical Laboratory Fee Schedule;2.Claim study	2019
36006606	7200	1L aMel	Claim study	2020
36529626	0	1L aRCC	Modeling study	2021
35658806	0	1L aNSCLC	Claim study	2020
30710066	0	1L aRCC	NA	2017
31992108	6650	1L aRCC	Claim study	2018
Abnormal liver function				
33417490	16290	1L uHCC	1.Claim study;2.Modeling study	2016
36006606	121	1L aMel	Claim study	2020
Acute kidney injury				
36006606	9719	1L aMel	Claim study	2020
Adrenal insufficiency				
36006606	10878	1L aMel	Claim study	2020
Alanine aminotransferase increased				
33556230	0	1L uHCC	NA	2020
34762112	6094	1L aMel	1.Claim study;2.Modeling study	2020
34991104	8719	1L aRCC	1.Clinical Laboratory Fee Schedule;2.Claim study	2019
36518883	0	1L uHCC	1.Claim study;2.RED BOOK;3.Modeling study	2021
36006606	121	1L aMel	Claim study	2020
35957949	298	2L MBC	NA	2020
31992108	7456	1L aRCC	Claim study	2018
32918790	216	1L aRCC	Modelling study	2019
Alopecia				
36006606	0	1L aMel	Claim study	2020
31287562	0	1L aNSCLC	1.Modeling study;2.Claim study	2018
Amylase increased				
34762112	6094	1L aMel	1.Claim study;2.Modeling study	2020
36006606	897	1L aMel	Claim study	2020
Anemia				

© 2025 Zhao M et al. *JAMA Network Open*.

PMID	Unit Cost/$	Disease	Source of Cost	Year of Cost
36160459	20260	1L MBC	1.Claim study;2.Modeling study	2022
30458884	4638	1L aRCC	1.Modeling study;2.Claim study;3.prospective or retrospective studies	2017
32457618	1947	1L aRCC	Modeling study	2019
35924662	4368	1L MUC	Modeling study	2018
30563395	3238	1L aMel	Claim study	2016
34991104	8464	1L aRCC	1.Clinical Laboratory Fee Schedule;2.Claim study	2019
36006606	8113	1L aMel	Claim study	2020
32874210	4638	1L aRCC	1.Claim study;2.Cost Systematic Review	2019
36529626	71	1L aRCC	Modeling study	2021
26332527	2787	1L aMel	Modeling study	2013
29456880	3238	1L aRCC	Claim study	2017
36639851	21921	1L aMel	1.Cost Systematic Review;2.Modeling study	2022
28301684	5949	2L aRCC	Modeling study	2014
32826180	7534	1L MUC	Claim study	2018
36780838	33585	2L MBC	Claim study	2021
34512315	20260	1L aNSCLC	Modeling study	2020
36071854	51337	1L MUC	Claim study	2021
37537015	25325	3L MBC	Modeling study	2022
36653947	4353	1L aNSCLC	Claim study	2021
30268469	20260	1L aNSCLC	Claim study	2018
35957949	1401	2L MBC	NA	2020
31610828	6461	1L NSCLC	Modeling study	2019
34778047	13110	3L MBC	Claim study	2020
18295368	2969	2L NSCLC	1.Claim study;2.Modeling study;3.Retrospective database analysis	2007
36532255	14093	3L MBC	1.Claim study;2.Modeling study;3.Retrospective database analysis	2021
33938936	5243	1L aNSCLC	1.Cost Systematic Review;2.Modeling study	2020
34150798	14532	2L MBC	1.Claim study;2.Cost Systematic Review	2019
31738117	13887	1L aNSCLC	Claim study	2019
31104762	13679	2L MBC	Retrospective cohort database analysis	2018
34734033	8779	1L aNSCLC	Modeling study	2020
31287562	20260	1L aNSCLC	1.Modeling study;2.Claim study	2018
26122345	6811	1L aNSCLC	Claim study	2013
32193809	7970	1L aNSCLC	Modeling study	2019
37854153	3698	1L aNSCLC	Modeling study	2023
35659172	8425	1L aNSCLC	NA	2021
35658806	8237	1L aNSCLC	Claim study	2020

© 2025 Zhao M et al. *JAMA Network Open*.

PMID	Unit Cost/$	Disease	Source of Cost	Year of Cost
32524512	7437	1L aNSCLC	Claim study	2018
32811247	20961	1L aNSCLC	1.Modeling study;2.Claim study	2019
33014826	7969	1L aNSCLC	Modeling study	2019
31992108	7428	1L aRCC	Claim study	2018
32918790	69	1L aRCC	Modelling study	2019
23057750	851	2L aMel	Claim study	2010
Arthralgia				
34762112	2524	1L aMel	1.Claim study;2.Modeling study	2020
36006606	7270	1L aMel	Claim study	2020
17592673	507	early-stage BC	1.Claim study;2.Red book;3.Retrospective cohort database analysis	2006
31992108	8797	1L aRCC	Claim study	2018
Ascites				
36006606	10192	1L aMel	Claim study	2020
Aspartate aminotransferase increased				
33556230	0	1L uHCC	NA	2020
36006606	121	1L aMel	Claim study	2020
34991104	8719	1L aRCC	1.Clinical Laboratory Fee Schedule;2.Claim study	2019
31992108	7456	1L aRCC	Claim study	2018
32918790	216	1L aRCC	Modelling study	2019
Asthenia				
33825837	2693	1L uHCC	Modeling study	2020
34762112	8999	1L aMel	1.Claim study;2.Modeling study	2020
34991104	9921	1L aRCC	1.Clinical Laboratory Fee Schedule;2.Claim study	2019
36006606	7843	1L aMel	Claim study	2020
18295368	122	2L NSCLC	1.Claim study;2.Modeling study;3.Retrospective database analysis	2007
33938936	1065	1L aNSCLC	1.Cost Systematic Review;2.Modeling study	2020
26122345	6304	1L aNSCLC	Claim study	2013
31992108	7243	1L aRCC	Claim study	2018
Back pain				
34991104	10912	1L aRCC	1.Clinical Laboratory Fee Schedule;2.Claim study	2019
36006606	2512	1L aMel	Claim study	2020
31992108	7223	1L aRCC	Claim study	2018
Blood creatine phosphokinase level increase				
34762112	6094	1L aMel	1.Claim study;2.Modeling study	2020
36006606	10821	1L aMel	Claim study	2020
Blood bilirubin increased				
33668100	0	1L uHCC	1.Claim study;2.Modeling study	2020

© 2025 Zhao M et al. *JAMA Network Open*.

PMID	Unit Cost/$	Disease	Source of Cost	Year of Cost
33556230	0	1L uHCC	NA	2020
34762112	6094	1L aMel	1.Claim study;2.Modeling study	2020
36518883	1088	1L uHCC	1.Claim study;2.RED BOOK;3.Modeling study	2021
30268469	0	1L aNSCLC	Claim study	2018
Chills				
36006606	6095	1L aMel	Claim study	2020
Colitis				
36006606	11045	1L aMel	Claim study	2020
36639851	21865	1L aMel	1.Cost Systematic Review;2.Modeling study	2022
36653947	6079	1L aNSCLC	Claim study	2021
30268469	6079	1L aNSCLC	Claim study	2018
23057750	8563	2L aMel	Claim study	2010
Constipation				
36006606	6749	1L aMel	Claim study	2020
36639851	22667	1L aMel	1.Cost Systematic Review;2.Modeling study	2022
36653947	2591	1L aNSCLC	Claim study	2021
30268469	2591	1L aNSCLC	Claim study	2018
31992108	6234	1L aRCC	Claim study	2018
Cough				
36006606	121	1L aMel	Claim study	2020
36639851	17186	1L aMel	1.Cost Systematic Review;2.Modeling study	2022
31992108	5640	1L aRCC	Claim study	2018
Decreased appetite				
33825837	12875	1L uHCC	Modeling study	2020
34762112	21089	1L aMel	1.Claim study;2.Modeling study	2020
34991104	12486	1L aRCC	1.Clinical Laboratory Fee Schedule;2.Claim study	2019
36006606	10460	1L aMel	Claim study	2020
33938936	160	1L aNSCLC	1.Cost Systematic Review;2.Modeling study	2020
33014826	9711	1L aNSCLC	Modeling study	2019
31992108	9661	1L aRCC	Claim study	2018
Dehydration				
36006606	6586	1L aMel	Claim study	2020
Dermatitis				
34762112	59	1L aMel	1.Claim study;2.Modeling study	2020
36639851	16997	1L aMel	1.Cost Systematic Review;2.Modeling study	2022
34093040	4482	1L aNSCLC	Modeling study	2018
Diabetic ketoacidosis				
36006606	8418	1L aMel	Claim study	2020
Diarrhea				

© 2025 Zhao M et al. *JAMA Network Open*.

PMID	Unit Cost/$	Disease	Source of Cost	Year of Cost
33625508	3802	1L uHCC	1.Claim study;2.RED BOOK;3.Modeling study	2019
33668100	88	1L uHCC	1.Claim study;2.Modeling study	2020
33556230	17	1L uHCC	NA	2020
32878521	1010	1L aRCC	Modeling study	2018
33417490	84	1L uHCC	1.Claim study;2.Modeling study	2016
32457618	5991	1L aRCC	Modeling study	2019
37271697	10487	1L aRCC	Modeling study	2022
33825837	1009	1L uHCC	Modeling study	2020
34991104	8620	1L aRCC	1.Clinical Laboratory Fee Schedule;2.Claim study	2019
19117341	5374	1L MBC	Retrospective cohort database analysis	2007
36599117	6737	1L aRCC	1.Modelling study;2.Claim study	2021
36653848	6504	1L aMel	Modeling study	2021
36518883	18065	1L uHCC	1.Claim study;2.RED BOOK;3.Modeling study	2021
36006606	8359	1L aMel	Claim study	2020
30477000	10760	1L aMel	Modelling study	2017
36529626	86	1L aRCC	Modeling study	2021
26171248	1083	1L aMel	1.Claim study;2.Red book;3.Retrospective cohort database analysis	2013
36639851	17864	1L aMel	1.Cost Systematic Review;2.Modeling study	2022
32826180	7568	1L MUC	Claim study	2018
36780838	31805	2L MBC	Claim study	2021
34512315	16510	1L aNSCLC	Modeling study	2020
36653947	3265	1L aNSCLC	Claim study	2021
30268469	16510	1L aNSCLC	Claim study	2018
35957949	6	2L MBC	NA	2020
34778047	3866	3L MBC	Claim study	2020
18295368	125	2L NSCLC	1.Claim study;2.Modeling study;3.Retrospective database analysis	2007
33938936	170	1L aNSCLC	1.Cost Systematic Review;2.Modeling study	2020
34150798	11545	2L MBC	1.Claim study;2.Cost Systematic Review	2019
34093040	16510	1L aNSCLC	Modeling study	2018
31287562	16510	1L aNSCLC	1.Modeling study;2.Claim study	2018
32193809	10301	1L aNSCLC	Modeling study	2019
36503033	10037	1-2L NSCLC	Claim study	2021
32811247	17081	1L aNSCLC	1.Modeling study;2.Claim study	2019
33014826	10301	1L aNSCLC	Modeling study	2019
30710066	6734	1L aRCC	NA	2017
31992108	7622	1L aRCC	Claim study	2018
32918790	85	1L aRCC	Modelling study	2019

© 2025 Zhao M et al. *JAMA Network Open*.

PMID	Unit Cost/$	Disease	Source of Cost	Year of Cost
23057750	775	2L aMel	Claim study	2010
Dizziness				
36006606	6636	1L aMel	Claim study	2020
31992108	6128	1L aRCC	Claim study	2018
Dry skin				
34762112	10323	1L aMel	1.Claim study;2.Modeling study	2020
31992108	6387	1L aRCC	Claim study	2018
Dyspnea				
34991104	7770	1L aRCC	1.Clinical Laboratory Fee Schedule;2.Claim study	2019
36006606	3598	1L aMel	Claim study	2020
36639851	22731	1L aMel	1.Cost Systematic Review;2.Modeling study	2022
18295368	122	2L NSCLC	1.Claim study;2.Modeling study;3.Retrospective database analysis	2007
36503033	7182	1-2L NSCLC	Claim study	2021
31992108	6413	1L aRCC	Claim study	2018
23057750	3345	2L aMel	Claim study	2010
Dyspepsia				
36639851	25088	1L aMel	1.Cost Systematic Review;2.Modeling study	2022
31992108	10027	1L aRCC	Claim study	2018
Fatigue				
33625508	249	1L uHCC	1.Claim study;2.RED BOOK;3.Modeling study	2019
36160459	6908	1L MBC	1.Claim study;2.Modeling study	2022
30458884	139	1L aRCC	1.Modeling study;2.Claim study;3.prospective or retrospective studies	2017
32878521	144	1L aRCC	Modeling study	2018
33417490	0	1L uHCC	1.Claim study;2.Modeling study	2016
33825837	2693	1L uHCC	Modeling study	2020
34762112	2682	1L aMel	1.Claim study;2.Modeling study	2020
34991104	9921	1L aRCC	1.Clinical Laboratory Fee Schedule;2.Claim study	2019
36006606	2669	1L aMel	Claim study	2020
30477000	6946	1L aMel	Modelling study	2017
30789633	0	1L aRCC	1.Modeling study;2.Claim study	2018
32874210	139	1L aRCC	1.Claim study;2.Cost Systematic Review	2019
32826180	7955	1L MUC	Claim study	2018
36780838	28725	2L MBC	Claim study	2021
34512315	0	1L aNSCLC	Modeling study	2020
37537015	1172	3L MBC	Modeling study	2022
36653947	733	1L aNSCLC	Claim study	2021
30268469	16185	1L aNSCLC	Claim study	2018

© 2025 Zhao M et al. *JAMA Network Open*.

PMID	Unit Cost/$	Disease	Source of Cost	Year of Cost
18295368	122	2L NSCLC	1.Claim study;2.Modeling study;3.Retrospective database analysis	2007
36532255	13088	3L MBC	1.Claim study;2.Modeling study;3.Retrospective database analysis	2021
33938936	1065	1L aNSCLC	1.Cost Systematic Review;2.Modeling study	2020
26122345	6304	1L aNSCLC	Claim study	2013
32193809	0	1L aNSCLC	Modeling study	2019
36503033	9649	1-2L NSCLC	Claim study	2021
35658806	7976	1L aNSCLC	Claim study	2020
32524512	10386	1L aNSCLC	Claim study	2018
32811247	16745	1L aNSCLC	1.Modeling study;2.Claim study	2019
33014826	0	1L aNSCLC	Modeling study	2019
30710066	0	1L aRCC	NA	2017
31992108	7243	1L aRCC	Claim study	2018
23057750	2069	2L aMel	Claim study	2010
Febrile neutropenia				
28472324	17052	1L MBC	1.Longitudinal discharge database;2.Claim study;3.Modeling study	2015
36006606	14658	1L aMel	Claim study	2020
32826180	13315	1L MUC	Claim study	2018
36780838	51384	2L MBC	Claim study	2021
34778047	22814	3L MBC	Claim study	2020
18295368	6260	2L NSCLC	1.Claim study;2.Modeling study;3.Retrospective database analysis	2007
31287562	17181	1L aNSCLC	1.Modeling study;2.Claim study	2018
26122345	12006	1L aNSCLC	Claim study	2013
37854153	19661	1L aNSCLC	Modeling study	2023
35659172	14075	1L aNSCLC	NA	2021
32524512	7786	1L aNSCLC	Claim study	2018
Fever/Pyrexia				
34762112	4283	1L aMel	1.Claim study;2.Modeling study	2020
36006606	122	1L aMel	Claim study	2020
31992108	7173	1L aRCC	Claim study	2018
23057750	3304	2L aMel	Claim study	2010
Gastrointestinal Bleeding				
33417490	9453	1L uHCC	1.Claim study;2.Modeling study	2016
33825837	23234	1L uHCC	Modeling study	2020
36639851	23655	1L aMel	1.Cost Systematic Review;2.Modeling study	2022
Guillain-Barré syndrome				

© 2025 Zhao M et al. *JAMA Network Open*.

PMID	Unit Cost/$	Disease	Source of Cost	Year of Cost
36006606	40054	1L aMel	Claim study	2020
Headache				
36006606	678	1L aMel	Claim study	2020
31992108	6893	1L aRCC	Claim study	2018
Hepatotoxicity				
33625508	2773	1L uHCC	1.Claim study;2.RED BOOK;3.Modeling study	2019
26332527	5915		Modeling study	2013
32826180	9409	1L MUC	Claim study	2018
34150798	7516	2L MBC	1.Claim study;2.Cost Systematic Review	2019
33364838	5915	1L MBC	1.Claim study;2.Retrospective cohort database analysis	2020
Hepatitis				
33825837	13287	1L uHCC	Modeling study	2020
36006606	17041	1L aMel	Claim study	2020
Hyperkeratosis				
36006606	6915	1L aMel	Claim study	2020
26171248	1083	1L aMel	1.Claim study;2.Red book;3.Retrospective cohort database analysis	2013
Hyperglycemia				
36006606	17621	1L aMel	Claim study	2020
28807351	222	1L aRCC	Claim study	2016
31992108	0	1L aRCC	Claim study	2018
Hyperthyroidism				
34762112	803	1L aMel	1.Claim study;2.Modeling study	2020
34991104	12379	1L aRCC	1.Clinical Laboratory Fee Schedule;2.Claim study	2019
36653947	2255	1L aNSCLC	Claim study	2021
30268469	20428	1L aNSCLC	Claim study	2018
Hypertriglyceridemia				
30563395	73	1L aMel	Claim study	2016
36529626	75	1L aRCC	Modeling study	2021
28807351	142	1L aRCC	Claim study	2016
29456880	73	1L aRCC	Claim study	2017
28301684	81	2L aRCC	Modeling study	2014
31992108	12231	1L aRCC	Claim study	2018
Hypertension				
33625508	1701	1L uHCC	1.Claim study;2.RED BOOK;3.Modeling study	2019
33668100	64	1L uHCC	1.Claim study;2.Modeling study	2020
33556230	35	1L uHCC	NA	2020
30458884	202	1L aRCC	1.Modeling study;2.Claim study;3.prospective or	2017

© 2025 Zhao M et al. *JAMA Network Open*.

			retrospective studies	
PMID	Unit Cost/$	Disease	Source of Cost	Year of Cost
32878521	209	1L aRCC	Modeling study	2018
33417490	61	1L uHCC	1.Claim study;2.Modeling study	2016
32457618	209	1L aRCC	Modeling study	2019
37271697	65	1L aRCC	Modeling study	2022
33825837	14704	1L uHCC	Modeling study	2020
34991104	9936	1L aRCC	1.Clinical Laboratory Fee Schedule;2.Claim study	2019
36599117	1927	1L aRCC	1.Modelling study;2.Claim study	2021
36653848	216	1L aMel	Modeling study	2021
36518883	1701	1L uHCC	1.Claim study;2.RED BOOK;3.Modeling study	2021
36006606	122	1L aMel	Claim study	2020
30789633	62	1L aRCC	1.Modeling study;2.Claim study	2018
32874210	202	1L aRCC	1.Claim study;2.Cost Systematic Review	2019
36529626	64	1L aRCC	Modeling study	2021
36639851	28571	1L aMel	1.Cost Systematic Review;2.Modeling study	2022
31287562	3232	1L aNSCLC	1.Modeling study;2.Claim study	2018
37854153	19661	1L aNSCLC	Modeling study	2023
32524512	9410	1L aNSCLC	Claim study	2018
30710066	480	1L aRCC	NA	2017
31992108	7287	1L aRCC	Claim study	2018
32918790	63	1L aRCC	Modelling study	2019
Hypothermia				
36006606	12124.68	1L aMel	Claim study	2020
Hypothyroidism				
34762112	803	1L aMel	1.Claim study;2.Modeling study	2020
36653947	2255	1L aNSCLC	Claim study	2021
30268469	20428	1L aNSCLC	Claim study	2018
31992108	9612.71	1L aRCC	Claim study	2018
Infections				
26332527	3642	1L aMel	Modeling study	2013
32826180	19670	1L MUC	Claim study	2018
18295368	122	2L NSCLC	1.Claim study;2.Modeling study;3.Retrospective database analysis	2007
34150798	14595	2L MBC	1.Claim study;2.Cost Systematic Review	2019
26122345	12713	1L aNSCLC	Claim study	2013
23057750	4331	2L aMel	Claim study	2010
33364838	12657	1L MBC	1.Claim study;2.Retrospective cohort database analysis	2020
Keratoacanthoma				

© 2025 Zhao M et al. *JAMA Network Open*.

PMID	Unit Cost/$	Disease	Source of Cost	Year of Cost
36006606	24755.94	1L aMel	Claim study	2020
26171248	113.67	1L aMel	1.Claim study;2.Red book;3.Retrospective cohort database analysis	2013
Malabsorption				
36006606	14152.14	1L aMel	Claim study	2020
Muscle spasm				
36006606	121.36	1L aMel	Claim study	2020
Myalgia				
34762112	2524	1L aMel	1.Claim study;2.Modeling study	2020
36006606	2511.78	1L aMel	Claim study	2020
23057750	1947	2L aMel	Claim study	2010
Myocardial infarction/angina				
36006606	52405.55	1L aMel	Claim study	2020
17592673	21783	early-stage BC	1.Claim study;2.Red book;3.Retrospective cohort database analysis	2006
36639851	21728	1L aMel	1.Cost Systematic Review;2.Modeling study	2022
Mucosal inflammation				
34991104	11040	1L aRCC	1.Clinical Laboratory Fee Schedule;2.Claim study	2019
31992108	10797.87	1L aRCC	Claim study	2018
Nausea				
33625508	2638	1L uHCC	1.Claim study;2.RED BOOK;3.Modeling study	2019
36160459	19134	1L MBC	1.Claim study;2.Modeling study	2022
32522057	7545	1L MBC	data not found in the referenced article	2019
34762112	1869	1L aMel	1.Claim study;2.Modeling study	2020
34991104	7583	1L aRCC	1.Clinical Laboratory Fee Schedule;2.Claim study	2019
36006606	3867.87	1L aMel	Claim study	2020
30477000	12117	1L aMel	Modelling study	2017
26171248	6666	1L aMel	1.Claim study;2.Red book;3.Retrospective cohort database analysis	2013
36639851	20927	1L aMel	1.Cost Systematic Review;2.Modeling study	2022
34512315	2586	1L aNSCLC	Modeling study	2020
36653947	1965	1L aNSCLC	Claim study	2021
30268469	19341	1L aNSCLC	Claim study	2018
35957949	36	2L MBC	NA	2020
34778047	3866	3L MBC	Claim study	2020
18295368	122	2L NSCLC	1.Claim study;2.Modeling study;3.Retrospective database analysis	2007
36532255	8342	3L MBC	1.Claim study;2.Modeling study;3.Retrospective database analysis	2021

© 2025 Zhao M et al. *JAMA Network Open*.

PMID	Unit Cost/$	Disease	Source of Cost	Year of Cost
33938936	160	1L aNSCLC	1.Cost Systematic Review;2.Modeling study	2020
26122345	6161	1L aNSCLC	Claim study	2013
36503033	10037	1-2L NSCLC	Claim study	2021
32811247	20010	1L aNSCLC	1.Modeling study;2.Claim study	2019
33014826	10301	1L aNSCLC	Modeling study	2019
31992108	6030.95	1L aRCC	Claim study	2018
23057750	1442	2L aMel	Claim study	2010
Neutropenia				
36160459	17181	1L MBC	1.Claim study;2.Modeling study	2022
32522057	24376	1L MBC	data not found in the referenced article	2019
32878521	1061	1L aRCC	Modeling study	2018
32457618	1061	1L aRCC	Modeling study	2019
35924662	5937	1L MUC	Modeling study	2018
34991104	17378	1L aRCC	1.Clinical Laboratory Fee Schedule;2.Claim study	2019
36518883	0	1L uHCC	1.Claim study;2.RED BOOK;3.Modeling study	2021
36006606	2324.73	1L aMel	Claim study	2020
36529626	36106	1L aRCC	Modeling study	2021
26332527	23238	1L aMel	Modeling study	2013
36639851	18590	1L aMel	1.Cost Systematic Review;2.Modeling study	2022
32826180	13286	1L MUC	Claim study	2018
36780838	51418	2L MBC	Claim study	2021
34512315	17181	1L aNSCLC	Modeling study	2020
36071854	36364	1L MUC	Claim study	2021
37537015	21477	3L MBC	Modeling study	2022
36653947	5321	1L aNSCLC	Claim study	2021
30268469	17181	1L aNSCLC	Claim study	2018
35957949	1074.867925	2L MBC	NA	2020
31610828	104	1L NSCLC	Modeling study	2019
34778047	9497	3L MBC	Claim study	2020
18295368	12178	2L NSCLC	1.Claim study;2.Modeling study;3.Retrospective database analysis	2007
36532255	4804	3L MBC	1.Claim study;2.Modeling study;3.Retrospective database analysis	2021
33938936	16857	1L aNSCLC	1.Cost Systematic Review;2.Modeling study	2020
34026637	9649	1L MBC	Retrospective cohort database analysis	2019
36920662	7818	1L MBC	Retrospective database analysis	2022
34150798	16256	2L MBC	1.Claim study;2.Cost Systematic Review	2019
31738117	13887	1L aNSCLC	Claim study	2019

© 2025 Zhao M et al. *JAMA Network Open*.

PMID	Unit Cost/$	Disease	Source of Cost	Year of Cost
31104762	9910	2L MBC	Retrospective cohort database analysis	2018
34734033	36346	1L aNSCLC	Modeling study	2020
31287562	17181	1L aNSCLC	1.Modeling study;2.Claim study	2018
26122345	12006	1L aNSCLC	Claim study	2013
32193809	32995	1L aNSCLC	Modeling study	2019
37854153	23184	1L aNSCLC	Modeling study	2023
35659172	14074.55	1L aNSCLC	NA	2021
31655368	32995	1L aNSCLC	Modeling study	2018
35658806	13760.9	1L aNSCLC	Claim study	2020
32524512	12533	1L aNSCLC	Claim study	2018
32811247	17776	1L aNSCLC	1.Modeling study;2.Claim study	2019
33014826	32995	1L aNSCLC	Modeling study	2019
31992108	12636.94	1L aRCC	Claim study	2018
32918790	35514	1L aRCC	Modelling study	2019
23057750	859	2L aMel	Claim study	2010
30847728	4433	1L MBC	Claim study	2016
33364838	17181	1L MBC	1.Claim study;2.Retrospective cohort database analysis	2020
Neutrophil count decreased				
35924662	1424.88	1L MUC	Modeling study	2018
34991104	8.63	1L aRCC	1.Clinical Laboratory Fee Schedule;2.Claim study	2019
36518883	0	1L uHCC	1.Claim study;2.RED BOOK;3.Modeling study	2021
36006606	2324.73	1L aMel	Claim study	2020
36529626	36106	1L aRCC	Modeling study	2021
36071854	51308	1L MUC	Claim study	2021
33938936	907	1L aNSCLC	1.Cost Systematic Review;2.Modeling study	2020
31738117	13887	1L aNSCLC	Claim study	2019
35659172	14074.55	1L aNSCLC	NA	2021
31655368	32995	1L aNSCLC	Modeling study	2018
33014826	32995	1L aNSCLC	Modeling study	2019
31992108	13537.31	1L aRCC	Claim study	2018
32918790	35514	1L aRCC	Modelling study	2019
QT prolongation				
36006606	121.36	1L aMel	Claim study	2020
33364838	25236	1L MBC	1.Claim study;2.Retrospective cohort database analysis	2020
Leukopenia				
36160459	17181	1L MBC	1.Claim study;2.Modeling study	2022
36006606	8723	1L aMel	Claim study	2020

© 2025 Zhao M et al. *JAMA Network Open*.

PMID	Unit Cost/$	Disease	Source of Cost	Year of Cost
26171248	0	1L aMel	1.Claim study;2.Red book;3.Retrospective cohort database analysis	2013
26332527	17181	1L aMel	Modeling study	2013
36639851	18590	1L aMel	1.Cost Systematic Review;2.Modeling study	2022
32826180	7089	1L MUC	Claim study	2018
36780838	30434	2L MBC	Claim study	2021
37537015	21477	3L MBC	Modeling study	2022
35957949	650	2L MBC	NA	2020
34778047	9497	3L MBC	Claim study	2020
36532255	12749	3L MBC	1.Claim study;2.Modeling study;3.Retrospective database analysis	2021
34026637	4934	1L MBC	Retrospective cohort database analysis	2019
26122345	8892	1L aNSCLC	Claim study	2013
31992108	8642.24	1L aRCC	Claim study	2018
33364838	17181	1L MBC	1.Claim study;2.Retrospective cohort database analysis	2020
Palmar-plantar erythrodysesthesia/Hand-foot syndrome				
33625508	987	1L uHCC	1.Claim study;2.RED BOOK;3.Modeling study	2019
33556230	107	1L uHCC	NA	2020
30458884	118.8	1L aRCC	1.Modeling study;2.Claim study;3.prospective or retrospective studies	2017
32878521	123	1L aRCC	Modeling study	2018
32457618	123	1L aRCC	Modeling study	2019
37271697	45.62	1L aRCC	Modeling study	2022
33825837	8382.19	1L uHCC	Modeling study	2020
34991104	7632	1L aRCC	1.Clinical Laboratory Fee Schedule;2.Claim study	2019
36599117	48	1L aRCC	1.Modelling study;2.Claim study	2021
36653848	127	1L aMel	Modeling study	2021
36518883	987	1L uHCC	1.Claim study;2.RED BOOK;3.Modeling study	2021
30789633	43.64	1L aRCC	1.Modeling study;2.Claim study	2018
32874210	119	1L aRCC	1.Claim study;2.Cost Systematic Review	2019
26171248	113.67	1L aMel	1.Claim study;2.Red book;3.Retrospective cohort database analysis	2013
30710066	0	1L aRCC	NA	2017
31992108	7155.86	1L aRCC	Claim study	2018
33668100	145.65	1L uHCC	1.Claim study;2.Modeling study	2020
33417490	138.28	1L uHCC	1.Claim study;2.Modeling study	2016
Pain				
36006606	2511.78	1L aMel	Claim study	2020

© 2025 Zhao M et al. *JAMA Network Open*.

PMID	Unit Cost/$	Disease	Source of Cost	Year of Cost
36639851	31360	1L aMel	1.Cost Systematic Review;2.Modeling study	2022
23057750	1947	2L aMel	Claim study	2010
Pancreatitis				
36006606	11210.59	1L aMel	Claim study	2020
36639851	35617	1L aMel	1.Cost Systematic Review;2.Modeling study	2022
30268469	15943	1L aNSCLC	Claim study	2018
Platelet count decrease				
33556230	0	1L uHCC	NA	2020
33825837	1111.38	1L uHCC	Modeling study	2020
35924662	4014	1L MUC	Modeling study	2018
34991104	8.63	1L aRCC	1.Clinical Laboratory Fee Schedule;2.Claim study	2019
36518883	0	1L uHCC	1.Claim study;2.RED BOOK;3.Modeling study	2021
36529626	9709	1L aRCC	Modeling study	2021
30268469	0	1L aNSCLC	Claim study	2018
31738117	16586	1L aNSCLC	Claim study	2019
32193809	5309	1L aNSCLC	Modeling study	2019
31992108	10538.27	1L aRCC	Claim study	2018
32918790	9550.4	1L aRCC	Modelling study	2019
Pulmonary				
36006606	11785.10	1L aMel	Claim study	2020
26332527	9320	1L aMel	Modeling study	2013
18295368	122	2L NSCLC	1.Claim study;2.Modeling study;3.Retrospective database analysis	2007
Peripheral oedema				
34762112	10323	1L aMel	1.Claim study;2.Modeling study	2020
36006606	121.36	1L aMel	Claim study	2020
36503033	9649	1-2L NSCLC	Claim study	2021
31992108	6931.32	1L aRCC	Claim study	2018
Peripheral Neuropathy				
32522057	7487	1L MBC	data not found in the referenced article	2019
36639851	30734	1L aMel	1.Cost Systematic Review;2.Modeling study	2022
Photosensitivity reaction				
34762112	5928	1L aMel	1.Claim study;2.Modeling study	2020
36006606	6085.66	1L aMel	Claim study	2020
Pleural effusion				
36006606	9571.7	1L aMel	Claim study	2020
36503033	11162	1-2L NSCLC	Claim study	2021
Pneumonia				
36006606	16781.93	1L aMel	Claim study	2020

© 2025 Zhao M et al. *JAMA Network Open*.

PMID	Unit Cost/$	Disease	Source of Cost	Year of Cost
36639851	23727	1L aMel	1.Cost Systematic Review;2.Modeling study	2022
32826180	10151	1L MUC	Claim study	2018
31738117	7924	1L aNSCLC	Claim study	2019
36503033	13503	1-2L NSCLC	Claim study	2021
Pneumonitis				
33417490	12579	1L uHCC	1.Claim study;2.Modeling study	2016
34762112	15426	1L aMel	1.Claim study;2.Modeling study	2020
36006606	31541.97	1L aMel	Claim study	2020
28807351	41	1L aRCC	Claim study	2016
36639851	23727	1L aMel	1.Cost Systematic Review;2.Modeling study	2022
36653947	9941	1L aNSCLC	Claim study	2021
30268469	21929	1L aNSCLC	Claim study	2018
31610828	6491	1L NSCLC	Modeling study	2019
31738117	11896	1L aNSCLC	Claim study	2019
36503033	13503	1-2L NSCLC	Claim study	2021
31992108	18598.88	1L aRCC	Claim study	2018
Proteinuria				
33625508	1728	1L uHCC	1.Claim study;2.RED BOOK;3.Modeling study	2019
33825837	3194	1L uHCC	Modeling study	2020
34991104	5017	1L aRCC	1.Clinical Laboratory Fee Schedule;2.Claim study	2019
31992108	4648.63	1L aRCC	Claim study	2018
Pruritus				
34762112	8030	1L aMel	1.Claim study;2.Modeling study	2020
36006606	7503.05	1L aMel	Claim study	2020
36639851	28318	1L aMel	1.Cost Systematic Review;2.Modeling study	2022
36653947	1184	1L aNSCLC	Claim study	2021
33938936	272	1L aNSCLC	1.Cost Systematic Review;2.Modeling study	2020
31992108	6929.12	1L aRCC	Claim study	2018
Rash				
33556230	1.44	1L uHCC	NA	2020
33825837	284.57	1L uHCC	Modeling study	2020
34762112	59	1L aMel	1.Claim study;2.Modeling study	2020
34991104	8528	1L aRCC	1.Clinical Laboratory Fee Schedule;2.Claim study	2019
36006606	154.76	1L aMel	Claim study	2020
36639851	16997	1L aMel	1.Cost Systematic Review;2.Modeling study	2022
34512315	7872	1L aNSCLC	Modeling study	2020
36653947	940	1L aNSCLC	Claim study	2021
18295368	200	2L NSCLC	1.Claim study;2.Modeling study;3.Retrospective database analysis	2007

© 2025 Zhao M et al. *JAMA Network Open*.

PMID	Unit Cost/$	Disease	Source of Cost	Year of Cost
33938936	272	1L aNSCLC	1.Cost Systematic Review;2.Modeling study	2020
34093040	4482	1L aNSCLC	Modeling study	2018
26122345	4482	1L aNSCLC	Claim study	2013
32811247	1054	1L aNSCLC	1.Modeling study;2.Claim study	2019
31992108	5315.69	1L aRCC	Claim study	2018
Serous retinopathy				
36006606	121.36	1L aMel	Claim study	2020
Squamous-cell carcinoma				
36006606	420.86	1L aMel	Claim study	2020
26171248	1595	1L aMel	1.Claim study;2.Red book;3.Retrospective cohort database analysis	2013
Stomatitis				
36006606	18229.32	1L aMel	Claim study	2020
34991104	17520	1L aRCC	1.Clinical Laboratory Fee Schedule;2.Claim study	2019
26332527	47.4	1L aMel	Modeling study	2013
28301684	10074	2L aRCC	Modeling study	2014
30268469	1695	1L aNSCLC	Claim study	2018
37537015	10797.87	3L MBC	Modeling study	2022
Thrombocyopenia				
33625508	4094	1L uHCC	1.Claim study;2.RED BOOK;3.Modeling study	2019
36160459	22698	1L MBC	1.Claim study;2.Modeling study	2022
33556230	4014	1L uHCC	NA	2020
32878521	4155	1L aRCC	Modeling study	2018
32874210	4014	1L aRCC	1.Claim study;2.Cost Systematic Review	2019
36639851	24559	1L aMel	1.Cost Systematic Review;2.Modeling study	2022
34734033	5848	1L aNSCLC	Modeling study	2020
32918790	9550.4	1L aRCC	Modelling study	2019
23057750	854	2L aMel	Claim study	2010
Thromboembolic Events				
36639851	28219	1L aMel	1.Cost Systematic Review;2.Modeling study	2022
17592673	15424	early-stage BC	1.Claim study;2.Red book;3.Retrospective cohort database analysis	2006
Vomiting				
34762112	1869	1L aMel	1.Claim study;2.Modeling study	2020
34991104	7315	1L aRCC	1.Clinical Laboratory Fee Schedule;2.Claim study	2019
36006606	3867.87	1L aMel	Claim study	2020
26171248	274.58	1L aMel	1.Claim study;2.Red book;3.Retrospective cohort database analysis	2013
36639851	18285	1L aMel	1.Cost Systematic Review;2.Modeling study	2022

© 2025 Zhao M et al. *JAMA Network Open*.

PMID	Unit Cost/$	Disease	Source of Cost	Year of Cost
32826180	5300	1L MUC	Claim study	2018
36653947	895	1L aNSCLC	Claim study	2021
30268469	16899	1L aNSCLC	Claim study	2018
35957949	37	2L MBC	NA	2020
34778047	3866	3L MBC	Claim study	2020
33938936	160	1L aNSCLC	1.Cost Systematic Review;2.Modeling study	2020
26122345	6161	1L aNSCLC	Claim study	2013
36503033	10037	1-2L NSCLC	Claim study	2021
33014826	10301	1L aNSCLC	Modeling study	2019
31992108	5532.57	1L aRCC	Claim study	2018
23057750	1442	2L aMel	Claim study	2010
Weight decreased/Anorexia				
34991104	9503	1L aRCC	1.Clinical Laboratory Fee Schedule;2.Claim study	2019
36006606	10460.18	1L aMel	Claim study	2020
31992108	8670.72	1L aRCC	Claim study	2018
36653947	3700	1L aNSCLC	Claim study	2021
18295368	122	2L NSCLC	1.Claim study;2.Modeling study;3.Retrospective database analysis	2007
White-cell count decreased				
34991104	8.63	1L aRCC	1.Clinical Laboratory Fee Schedule;2.Claim study	2019
30268469	0	1L aNSCLC	Claim study	2018
31738117	13897	1L aNSCLC	Claim study	2019
31655368	32995	1L aNSCLC	Modeling study	2018
31992108	8642.24	1L aRCC	Claim study	2018
γ -Glutamyltransferase concentration increase				
36006606	5584.7	1L aMel	Claim study	2020

Abbreviation: 1L, first-line; 2L, second-line; AE, adverse event; aNSCLC, advanced non-small cell lung cancer; aMel, advanced melanoma; aRCC, advanced renal cell carcinoma; CEA, cost-effectiveness analysis; Chemo, chemotherapy; ICI, immune checkpoint inhibitor; MBC, metastatic breast cancer; MUC, metastatic urothelial cancer; TD, targeted drug; uHCC, unresectable hepatocellular carcinoma.

* Without CPI adjusted

© 2025 Zhao M et al. *JAMA Network Open*.

eTable 7. Included Real-world and CEA Studies for Comparison of AE Cost or Cost Proportion Between CEAs and Real-World Data

Type of Study	Pubmed ID	Treatment	Disease	Year of study
A. AE Cost Proportion by Different Therapy Mechanisms				
Comparison 1 (Real-world Study, 1; CEA, 1)				
Real-world Study	25882026	Targeted therapy	First-line aMel	2015
CEA	26171248	Targeted therapy	First-line aMel	2015
Comparison 2 (Real-world Study, 1; CEA, 5)				
Real-world Study	25882026	Immune checkpoint inhibitor	First-line aMel	2015
CEA	28221865	Immune checkpoint inhibitor	First-line aMel	2017
CEA	36006606	Immune checkpoint inhibitor	First-line aMel	2022
CEA	30477000	Immune checkpoint inhibitor	First-line aMel	2019
CEA	32697281	Immune checkpoint inhibitor	First-line aMel	2020
CEA	23057750	Immune checkpoint inhibitor	First-line aMel	2013
Comparison 3 (Real-world Study, 1; CEA, 1)				
Real-world Study	25882026	Chemotherapy	First-line aMel	2022
CEA	28221865	Chemotherapy	First-line aMel	2021
Comparison 4 (Real-world Study, 1; CEA, 1)				
Real-world Study	34494389	Targeted therapy	First-line uHCC	2022
CEA	33556230	Targeted therapy	First-line uHCC	2021
Comparison 5 (Real-world Study, 1; CEA, 2)				
Real-world Study	26308231	Targeted therapy	First-line aRCC	2015
CEA	18711190	Targeted therapy	First-line aRCC	2008
CEA	30710066	Targeted therapy	First-line aRCC	2019
Comparison 6 (Real-world Study, 1; CEA, 7)				
Real-world Study	29885945	Chemotherapy	First-line aNSCLC	2018
CEA	21914503	Chemotherapy	First-line aNSCLC	2011
CEA	30649973	Chemotherapy	First-line aNSCLC	2019
CEA	26122345	Chemotherapy	First-line aNSCLC	2015
CEA	35659172	Chemotherapy	First-line aNSCLC	2022
CEA	34736841	Chemotherapy	First-line aNSCLC	2021
CEA	35094793	Chemotherapy	First-line aNSCLC	2022
CEA	35658806	Chemotherapy	First-line aNSCLC	2022
Comparison 7 (Real-world Study, 1; CEA, 1)				
Real-world Study	29885945	Targeted therapy+ chemotherapy	First-line aNSCLC	2018
CEA	21914503	Targeted therapy+ chemotherapy	First-line aNSCLC	2011
Comparison 8 (Real-world Study, 1; CEA, 1)				
Real-world Study	29983370	Chemotherapy	Second-line aNSCLC	2018
Type of Study	Pubmed ID	Treatment	Disease	Year of study

© 2025 Zhao M et al. *JAMA Network Open*.

Type of Study	Pubmed ID	Treatment	Disease	Year of study
CEA	18295368	Chemotherapy	Second-line aNSCLC	2008
Comparison 9 (Real-world Study, 3; CEA, 1)				
Real-world Study	20051811	Chemotherapy	First-line MBC	2010
Real-world Study	27032967	Chemotherapy	First-line MBC	2016
Real-world Study	25085897	Chemotherapy	First-line MBC	2014
CEA	35796042	Chemotherapy	First-line MBC	2022
Comparison 10 (Real-world Study, 1; CEA, 2)				
Real-world Study	27032967	Chemotherapy	Second MBC	2016
CEA	37537015	Chemotherapy	Second MBC	2023
CEA	36532255	Chemotherapy	Second MBC	2022
B. AE Cost Proportion by Different Drugs				
Comparison 1 (Real-world Study, 1; CEA, 5)				
Real-world Study	25882026	Ipilimumab	First-line aMel	2015
CEA	28221865	Ipilimumab	First-line aMel	2017
CEA	36006606	Ipilimumab	First-line aMel	2022
CEA	30477000	Ipilimumab	First-line aMel	2019
CEA	32697281	Ipilimumab	First-line aMel	2020
CEA	23057750	Ipilimumab	First-line aMel	2013
Comparison 2 (Real-world Study, 1; CEA, 1)				
Real-world Study	25882026	Dacarbazine	First-line aMel	2015
CEA	28221865	Dacarbazine	First-line aMel	2017
Comparison 3 (Real-world Study, 1; CEA, 1)				
Real-world Study	25882026	Vemurafenib	First-line aMel	2015
CEA	26171248	Vemurafenib	First-line aMel	2015
Comparison 4 (Real-world Study, 1; CEA, 1)				
Real-world Study	34494389	Sorafenib	First-line uHCC	2022
CEA	33556230	Sorafenib	First-line uHCC	2021
Comparison 5 (Real-world Study, 1; CEA, 1)				
Real-world Study	26308231	Pazopanib	First-line aRCC	2015
CEA	30710066	Pazopanib	First-line aRCC	2019
Comparison 6 (Real-world Study, 1; CEA, 2)				
Real-world Study	26308231	Sunitinib	First-line aRCC	2015
CEA	30710066	Sunitinib	First-line aRCC	2019
CEA	18711190	Sunitinib	First-line aRCC	2008
Comparison 7 (Real-world Study, 1; CEA, 1)				
Real-world Study	29885945	Bevacizumab plus carboplatin and paclitaxel	First-line aNSCLC	2018
CEA	21914503	Bevacizumab plus carboplatin and paclitaxel	First-line aNSCLC	2011
Comparison 8 (Real-world Study, 1; CEA, 7)				
Real-world Study	29885945	Platinum-based Chemotherapy	First-line aNSCLC	2018

© 2025 Zhao M et al. *JAMA Network Open*.

Type of Study	Pubmed ID	Treatment	Disease	Year of study
CEA	21914503	Platinum-based Chemotherapy	First-line aNSCLC	2011
CEA	30649973	Platinum-based Chemotherapy	First-line aNSCLC	2019
CEA	26122345	Platinum-based Chemotherapy	First-line aNSCLC	2015
CEA	35659172	Platinum-based Chemotherapy	First-line aNSCLC	2022
CEA	34736841	Platinum-based Chemotherapy	First-line aNSCLC	2021
CEA	35094793	Platinum-based Chemotherapy	First-line aNSCLC	2022
CEA	35658806	Platinum-based Chemotherapy	First-line aNSCLC	2022
Comparison 9 (Real-world Study, 1; CEA, 1)				
Real-world Study	29983370	Docetaxel	Second-line aNSCLC	2018
CEA	18295368	Docetaxel	Second-line aNSCLC	2008
Comparison 10 (Real-world Study, 1; CEA, 1)				
Real-world Study	29983370	Pemetrexed	Second-line aNSCLC	2018
CEA	18295368	Pemetrexed	Second-line aNSCLC	2008
Comparison 11 (Real-world Study, 2; CEA, 1)				
Real-world Study	20051811	Taxane	First-line MBC	2010
Real-world Study	25085897	Taxane	First-line MBC	2014
CEA	35796042	Taxane	First-line MBC	2022
Comparison 12 (Real-world Study, 1; CEA, 2)				
Real-world Study	27032967	Taxane	Second-line MBC	2016
CEA	37537015	Taxane	Second-line MBC	2023
CEA	36532255	Taxane	Second-line MBC	2022
C. AE Cost ($) for Specific Treatments				
Comparison 1 (Real-world Study, 1; CEA, 26)				
Real-world Study	32463768	Chemotherapy	First-line aNSCLC	2020
CEA	34512315	Chemotherapy	First-line aNSCLC	2021
CEA	36653947	Chemotherapy	First-line aNSCLC	2023
CEA	30268469	Chemotherapy	First-line aNSCLC	2018
CEA	31610828	Chemotherapy	First-line aNSCLC	2020
CEA	34290602	Chemotherapy	First-line aNSCLC	2021
CEA	33938936	Chemotherapy	First-line aNSCLC	2021
CEA	31738117	Chemotherapy	First-line aNSCLC	2020
CEA	31831534	Chemotherapy	First-line aNSCLC	2020
CEA	34100243	Chemotherapy	First-line aNSCLC	2021
CEA	34734033	Chemotherapy	First-line aNSCLC	2021
CEA	33209600	Chemotherapy	First-line aNSCLC	2020
CEA	30649973	Chemotherapy	First-line aNSCLC	2019

© 2025 Zhao M et al. *JAMA Network Open*.

Type of Study	Pubmed ID	Treatment	Disease	Year of study
CEA	31287562	Chemotherapy	First-line aNSCLC	2020
CEA	31553470	Chemotherapy	First-line aNSCLC	2020
CEA	26122345	Chemotherapy	First-line aNSCLC	2015
CEA	32193809	Chemotherapy	First-line aNSCLC	2020
CEA	37854153	Chemotherapy	First-line aNSCLC	2023
CEA	35659172	Chemotherapy	First-line aNSCLC	2022
CEA	34736841	Chemotherapy	First-line aNSCLC	2021
CEA	31655368	Chemotherapy	First-line aNSCLC	2019
CEA	35094793	Chemotherapy	First-line aNSCLC	2022
CEA	35658806	Chemotherapy	First-line aNSCLC	2022
CEA	31995619	Chemotherapy	First-line aNSCLC	2020
CEA	32524512	Chemotherapy	First-line aNSCLC	2020
CEA	32811247	Chemotherapy	First-line aNSCLC	2020
CEA	33014826	Chemotherapy	First-line aNSCLC	2020
Comparison 2 (Real-world Study, 1; CEA, 14)				
Real-world Study	32463768	Immune checkpoint inhibitor	First-line aNSCLC	2020
CEA	34512315	Immune checkpoint inhibitor	First-line aNSCLC	2021
CEA	36653947	Immune checkpoint inhibitor	First-line aNSCLC	2023
CEA	30268469	Immune checkpoint inhibitor	First-line aNSCLC	2018
CEA	31610828	Immune checkpoint inhibitor	First-line aNSCLC	2020
CEA	34290602	Immune checkpoint inhibitor	First-line aNSCLC	2021
CEA	33938936	Immune checkpoint inhibitor	First-line aNSCLC	2021
CEA	31738117	Immune checkpoint inhibitor	First-line aNSCLC	2019
CEA	34734033	Immune checkpoint inhibitor	First-line aNSCLC	2021
CEA	35659172	Immune checkpoint inhibitor	First-line aNSCLC	2021
CEA	34736841	Immune checkpoint inhibitor	First-line aNSCLC	2022
CEA	35094793	Immune checkpoint inhibitor	First-line aNSCLC	2022
CEA	35658806	Immune checkpoint inhibitor	First-line aNSCLC	2022
CEA	31995619	Immune checkpoint inhibitor	First-line aNSCLC	2020
CEA	32811247	Immune checkpoint inhibitor	First-line aNSCLC	2020
Comparison 3 (Real-world Study, 1; CEA, 10)				
Real-world Study	32463768	Chemotherapy+ immune checkpoint inhibitor	First-line aNSCLC	2020
CEA	36653947	Chemotherapy+ immune checkpoint inhibitor	First-line aNSCLC	2023
CEA	31831534	Chemotherapy+ immune checkpoint inhibitor	First-line aNSCLC	2019
CEA	34100243	Chemotherapy+ immune checkpoint inhibitor	First-line aNSCLC	2021
CEA	33209600	Chemotherapy+ immune checkpoint inhibitor	First-line aNSCLC	2020
CEA	30649973	Chemotherapy+ immune checkpoint inhibitor	First-line aNSCLC	2019
CEA	31553470	Chemotherapy+ immune checkpoint inhibitor	First-line aNSCLC	2019
CEA	35658806	Chemotherapy+ immune checkpoint inhibitor	First-line aNSCLC	2022
CEA	31995619	Chemotherapy+ immune checkpoint inhibitor	First-line aNSCLC	2020

© 2025 Zhao M et al. *JAMA Network Open*.

Type of Study	Pubmed ID	Treatment	Disease	Year of study
CEA	32524512	Chemotherapy+ immune checkpoint inhibitor	First-line aNSCLC	2020
CEA	33014826	Chemotherapy+ immune checkpoint inhibitor	First-line aNSCLC	2020
Comparison 4 (Real-world Study, 1; CEA, 3)				
Real-world Study	25085897	Chemotherapy	First-line MBC	2014
CEA	32522057	Chemotherapy	First-line MBC	2020
CEA	32426048	Chemotherapy	First-line MBC	2020
CEA	35796042	Chemotherapy	First-line MBC	2022
Comparison 5 (Real-world Study, 1; CEA, 3)				
Real-world Study	34494389	Targeted therapy	First-line uHCC	2022
CEA	33625508	Targeted therapy	First-line uHCC	2021
CEA	33668100	Targeted therapy	First-line uHCC	2021
CEA	33556230	Targeted therapy	First-line uHCC	2021
CEA	33417490	Targeted therapy	First-line uHCC	2021
CEA	33825837	Targeted therapy	First-line uHCC	2021
Comparison 6 (Real-world Study, 1; CEA, 6)				
Real-world Study	25085897	Chemotherapy	Second-line MBC	2014
CEA	37085377	Chemotherapy	Second-line MBC	2023
CEA	36780838	Chemotherapy	Second-line MBC	2022
CEA	37537015	Chemotherapy	Second-line MBC	2023
CEA	37386633	Chemotherapy	Second-line MBC	2023
CEA	34778047	Chemotherapy	Second-line MBC	2021
CEA	36532255	Chemotherapy	Second-line MBC	2022
D. Relative AE Cost Differences Between Treatments				
Comparison 1 (Real-world Study, 1; CEA, 15)				
Real-world Study	32463768	Chemotherapy VS Immune checkpoint inhibitor	First-line aNSCLC	2020
CEA	34512315	Chemotherapy VS Immune checkpoint inhibitor	First-line aNSCLC	2021
CEA	36653947	Chemotherapy VS Immune checkpoint inhibitor	First-line aNSCLC	2023
CEA	30268469	Chemotherapy VS Immune checkpoint inhibitor	First-line aNSCLC	2018
CEA	31610828	Chemotherapy VS Immune checkpoint inhibitor	First-line aNSCLC	2020
CEA	34290602	Chemotherapy VS Immune checkpoint inhibitor	First-line aNSCLC	2021
CEA	33938936	Chemotherapy VS Immune checkpoint inhibitor	First-line aNSCLC	2020
CEA	31738117	Chemotherapy VS Immune checkpoint inhibitor	First-line aNSCLC	2020

© 2025 Zhao M et al. *JAMA Network Open*.

CEA	34734033	Chemotherapy VS Immune checkpoint inhibitor	First-line aNSCLC	2021
CEA	31287562	Chemotherapy VS Immune checkpoint inhibitor	First-line aNSCLC	2019
CEA	35659172	Chemotherapy VS Immune checkpoint inhibitor	First-line aNSCLC	2022
CEA	34736841	Chemotherapy VS Immune checkpoint inhibitor	First-line aNSCLC	2021
CEA	35094793	Chemotherapy VS Immune checkpoint inhibitor	First-line aNSCLC	2022
CEA	35658806	Chemotherapy VS Immune checkpoint inhibitor	First-line aNSCLC	2020
CEA	31995619	Chemotherapy VS Immune checkpoint inhibitor	First-line aNSCLC	2020
CEA	32811247	Chemotherapy VS Immune checkpoint inhibitor	First-line aNSCLC	2020
Type of Study	Pubmed ID	Treatment	Disease	Year of study
Comparison 2 (Real-world Study, 1; CEA, 10)				
Real-world Study	32463768	Chemotherapy VS Chemotherapy+Immune checkpoint inhibitor	First-line aNSCLC	2020
CEA	36653947	Chemotherapy VS Chemotherapy+Immune checkpoint inhibitor	First-line aNSCLC	2023
CEA	31831534	Chemotherapy VS Chemotherapy+Immune checkpoint inhibitor	First-line aNSCLC	2020
CEA	34100243	Chemotherapy VS Chemotherapy+Immune checkpoint inhibitor	First-line aNSCLC	2021
CEA	33209600	Chemotherapy VS Chemotherapy+Immune checkpoint inhibitor	First-line aNSCLC	2020
CEA	30649973	Chemotherapy VS Chemotherapy+Immune checkpoint inhibitor	First-line aNSCLC	2019
CEA	31553470	Chemotherapy VS Chemotherapy+Immune checkpoint inhibitor	First-line aNSCLC	2020
CEA	35658806	Chemotherapy VS Chemotherapy+Immune checkpoint inhibitor	First-line aNSCLC	2022
CEA	31995619	Chemotherapy VS Chemotherapy+Immune checkpoint inhibitor	First-line aNSCLC	2020
CEA	32524512	Chemotherapy VS Chemotherapy+Immune checkpoint inhibitor	First-line aNSCLC	2020
CEA	33014826	Chemotherapy VS Chemotherapy+Immune checkpoint inhibitor	First-line aNSCLC	2020

Abbreviation: AE, adverse event; aNSCLC, advanced non-small cell lung cancer; aMel, advanced melanoma; aRCC, advanced renal cell carcinoma; CEA, cost-effectiveness analysis; IQR, interquartile

© 2025 Zhao M et al. *JAMA Network Open*.

range; MBC, metastatic breast cancer; MUC, metastatic urothelial cancer; uHCC, unresectable hepatocellular carcinoma.

© 2025 Zhao M et al. *JAMA Network Open*.

eTable 8. Real-World Studies and CEAs Not Included in the Comparison Between CEAs and Real-World Studies

Type of Study	Pubmed ID	Disease	Reasons for not including AE cost comparison
Real-world Study	19738390	Resectable colorectal cancer	No matching cost-effectiveness analysis was identified, and CEA for Resectable colorectal cancer is focused on non-drug evaluation
Real-world Study	31835965	Non-metastatic prostate cancer	No matching cost-effectiveness analysis was identified. All included CEAs focused on metastatic prostate cancer
CEA	14693319	First-line MBC	Only total AE cost can be calculated based on CEA in CEA, No real-world study reported the total AE costs of first-line Endocrine therapy.
CEA	19117341	First-line MBC	AE cost was not reported, either in absolute terms or as a percentage of total cost
CEA	21039835	First-line uHCC	AE cost was not reported, only that the AE cost was $1.2/month for Sorafenib, and treatment duration was not reported.
CEA	21265994	First-line aRCC	Only total AE cost can be calculated based on CEA, but no real-world study reported the total AE costs of any First-line aRCC treatments
CEA	22200867	First-line MBC	AE cost was not reported, either in absolute terms or as a percentage of total cost
CEA	25198196	First-line aMel	CEA is not from a Payer perspective, but from a social perspective
CEA	26048087	Second-line MBC	Only total AE cost can be calculated based on CEA, No real-world study reported the total AE costs of second-line Endocrine therapy.
CEA	27654970	Second-line MBC	Only total AE cost can be calculated based on CEA, No real-world study reported the total AE costs of second-line Endocrine therapy.
CEA	28301684	Second-line aRCC	Only total AE cost can be calculated based on CEA, but no real-world study reported the total AE costs of any Second-line aRCC treatments
CEA	28472324	First-line MBC	Only total AE cost can be calculated based on CEA, No real-world study reported the total AE costs of first-line TKI drugs.

© 2025 Zhao M et al. *JAMA Network Open*.

Type of Study	Pubmed ID	Disease	Reasons for not including AE cost comparison
CEA	28530525	First-line aMel	CEA is not from a Payer perspective, but from a social perspective
CEA	29852038	First-line aNSCLC	Only total AE cost can be calculated based on CEA, No real-world study reported the total AE costs of EGFR-TKI drugs.
CEA	30458884	First-line aRCC	Only total AE cost can be calculated based on CEA, No real-world study reported the total AE costs of any First-line aRCC treatments
CEA	30563395	First-line aMel	AE cost was not reported in CEA, only that the AE cost was $26/month for ICI, and treatment duration was not reported.
CEA	30789633	First-line aRCC	Only total AE cost can be calculated based on CEA, but no real-world study reported the total AE costs of any First-line aRCC treatments
CEA	30847728	First-line MBC	Payment perspective not reported in CEA
CEA	31104762	Second-line MBC	Only total AE cost can be calculated based on CEA, No real-world study reported the total AE costs of second-line Endocrine therapy.
CEA	31853795	First-line MBC	Only total AE cost can be calculated based on CEA, No real-world study reported the total AE costs of first-line Endocrine therapy.
CEA	31945265	First-line aNSCLC	AE cost was not reported, only that the cyclely AE cost for Pembrolizumab + Chemotherapy were reported, and treatment duration was not reported.
CEA	31992108	First-line aRCC	Only total AE cost can be calculated based on CEA, but no real-world study reported the total AE costs of any First-line aRCC treatments
CEA	32457618	First-line aRCC	Only total AE cost can be calculated based on CEA, but no real-world study reported the total AE costs of any First-line aRCC treatments
CEA	32697113	First-line aRCC	Only total AE cost can be calculated based on CEA, but no real-world study reported the total AE costs of any First-line aRCC treatments
CEA	32826180	Metastatic urothelial cancer	No real-world analysis was identified. No real-world study included in this study had an indication of metastatic

© 2025 Zhao M et al. *JAMA Network Open*.

			urothelial cancer
Type of Study	Pubmed ID	Disease	Reasons for not including AE cost comparison
CEA	32918790	First-line aRCC	Only total AE cost can be calculated based on CEA, but no real-world study reported the total AE costs of any First-line aRCC treatments
CEA	33364838	First-line MBC	Only total AE cost can be calculated based on CEA, No real-world study reported the total AE costs of first-line Endocrine therapy.
CEA	33645243	First-line MBC	Only total AE cost can be calculated based on CEA, No real-world study reported the total AE costs of first-line Endocrine therapy.
CEA	33732316	First-line aNSCLC	Only total AE cost can be calculated based on CEA, No real-world study reported the total AE costs of EGFR-TKI drugs.
CEA	34019245	Metastatic urothelial cancer	No real-world analysis was identified. No real-world study included in this study had an indication of metastatic urothelial cancer
CEA	34026637	First-line MBC	Only total AE cost can be calculated based on CEA, No real-world study reported the total AE costs of first-line Endocrine therapy.
CEA	34093040	First-line aNSCLC	Only total AE cost can be calculated based on CEA, No real-world study reported the total AE costs of EGFR-TKI drugs.
CEA	34150798	Second-line MBC	Only total AE cost can be calculated based on CEA, No real-world study reported the total AE costs of second-line Endocrine therapy.
CEA	34268373	First-line aNSCLC	Only total AE cost can be calculated based on CEA, No real-world study reported the total AE costs of EGFR-TKI drugs.
CEA	34664194	First-line aRCC	Only total AE cost can be calculated based on CEA, No real-world study reported the total AE costs of any First-line aRCC treatments
CEA	34762112	First-line aMel	Only total AE cost can be calculated based on CEA, No real-world study reported the total AE costs of any First-line aMel treatments

© 2025 Zhao M et al. *JAMA Network Open*.

Type of Study	Pubmed ID	Disease	Reasons for not including AE cost comparison
CEA	34858820	First-line aMel	Neither the absolute value of AE costs nor the proportion of AE costs to total costs is reported or described in CEA.
CEA	34966275	First-line+Second-line aRCC	Only total AE cost can be calculated based on CEA, but no real-world study reported the total AE costs of any First-line or Second-line aRCC treatments
CEA	34991104	First-line aRCC	Only total AE cost can be calculated based on CEA, but no real-world study reported the total AE costs of any First-line aRCC treatments
CEA	35924662	Metastatic urothelial cancer	No real-world analysis was identified. No real-world study included in this study had an indication of metastatic urothelial cancer
CEA	35957949	Second-line MBC	Only total AE cost can be calculated based on CEA, No real-world study reported the total AE costs of second-line Endocrine therapy.
CEA	36071854	Metastatic urothelial cancer	No real-world analysis was identified. No real-world study included in this study had an indication of metastatic urothelial cancer
CEA	36160459	First-line MBC	No real-world analysis was identified. No real-world study reported the AE costs of ADC drugs.
CEA	36282933	First-line MBC	No real-world analysis was identified. No real-world study reported the AE costs of ADC drugs.
CEA	36327624	Second-line MBC	Only total AE cost can be calculated based on CEA, No real-world study reported the total AE costs of second-line ADC therapy.
CEA	36503033	First-line and Second-line NSCLC	Only total AE cost can be calculated based on CEA, No real-world studies reported AE costs for second-line NSCLC patients receiving targeted therapy
CEA	36529626	First-line aRCC	Only total AE cost can be calculated based on CEA, but no real-world study reported the total AE costs of any First-line aRCC treatments
CEA	36599117	First-line aRCC	Only total AE cost can be calculated based on CEA, but no real-world study reported the total AE costs of any First-line aRCC treatments
CEA	36639851	First-line aMel	No information on AE costs or the proportion of AE costs to total costs was reported in CEA

© 2025 Zhao M et al. *JAMA Network Open*.

CEA	36653848	First-line aMel	Only total AE cost can be calculated based on CEA, No real-world study reported the total AE costs of any First-line aMel treatments
CEA	36920662	First-line MBC	Only total AE cost can be calculated based on CEA, No real-world study reported the total AE costs of first-line Endocrine therapy.
CEA	37271697	First-line aRCC	Only total AE cost can be calculated based on CEA, but no real-world study reported the total AE costs of any First-line aRCC treatments

Abbreviation: AE, adverse event; aNSCLC, advanced non-small cell lung cancer; aMel, advanced melanoma; aRCC, advanced renal cell carcinoma; CEA, cost-effectiveness analysis; IQR, interquartile range; MBC, metastatic breast cancer; MUC, metastatic urothelial cancer; uHCC, unresectable hepatocellular carcinoma.

© 2025 Zhao M et al. *JAMA Network Open*.

eTable 9. Comparability Analysis of Real-World and CEA Evidence for AE Cost Proportions

Number	Pubmed ID	Disease	Treatment	Details	Study Year	Follow-up time of AE cost
Real-world	25882026	First-line aMel	Chemotherapy	Chemotherapy (dacarbazine)	2015	10 months (Median treatment duration: 1.7 months#[12])
Cost-effectiveness analysis	28221865	First-line aMel	Chemotherapy	Chemotherapy (dacarbazine)	2017	Full treatment duration
Real-world	25882026	First-line aMel	Chemotherapy	Chemotherapy (paclitaxel)	2015	10 months (Median treatment duration: 1.7 months#[12])
Real-world	25882026	First-line aMel	Chemotherapy	Chemotherapy (temozolomide)	2015	10 months (Median treatment duration: 1.7 months#[12])
Real-world	25882026	First-line aMel	ICI	ICI (ipilimumab)	2015	10 months (Median treatment duration: 2-3 months#[13])
Cost-effectiveness analysis	28221865	First-line aMel	ICI	ICI (Ipilimumab)	2017	Full treatment duration
Cost-effectiveness analysis	36006606	First-line aMel	ICI	ICI (ipilimumab)	2022	Full treatment duration
Cost-effectiveness analysis	30477000	First-line aMel	ICI	ICI (Ipilimumab)	2019	Full treatment duration

© 2025 Zhao M et al. *JAMA Network Open*.

Cost-effectiveness analysis	32697281	First-line aMel	ICI	ICI (Ipilimumab)	2020	Full treatment duration
Cost-effectiveness analysis	28221865	First-line aMel	ICI	ICI (Nivolumab)	2017	Full treatment duration
Cost-effectiveness analysis	36006606	First-line aMel	ICI	ICI (nivolumab)	2022	Full treatment duration
Cost-effectiveness analysis	28221865	First-line aMel	ICI	ICI (Nivolumab+Ipilimumab)	2017	Full treatment duration
Cost-effectiveness analysis	28221865	First-line aMel	ICI	ICI (pembrolizumab)	2017	Full treatment duration
Cost-effectiveness analysis	28221865	First-line aMel	ICI	ICI (pembrolizumab)	2017	Full treatment duration
Cost-effectiveness analysis	36006606	First-line aMel	ICI	ICI (pembrolizumab)	2022	Full treatment duration
Cost-effectiveness analysis	26171248	First-line aMel	Targeted treatment	Targeted treatment (Dabrafenib)	2015	Full treatment duration
Cost-effectiveness analysis	26171248	First-line aMel	Targeted treatment	Targeted treatment (Dacarbazine)	2015	Full treatment duration
Real-world	25882026	First-line aMel	Targeted treatment	Targeted treatment (vemurafenib)	2015	10 months (Median treatment duration: 5.3 months#[14])
Cost-effectiveness analysis	26171248	First-line aMel	Targeted treatment	Targeted treatment (Vemurafenib)	2015	Full treatment duration
Real-world	34494389	First-line uHCC	Targeted treatment	Targeted treatment (Sorafenib)	2021	12 months

© 2025 Zhao M et al. *JAMA Network Open*.

Cost-effectiveness analysis	33556230	First-line uHCC	Targeted treatment	Targeted treatment (Sorafenib)	2021	Full treatment duration
Real-world	26308231	First-line aRCC	Targeted treatment	Targeted treatment (sunitinib)	2015	10.6 months (Median treatment duration: 5.3 months#[14])
Real-world	26308231	First-line aRCC	Targeted treatment	Targeted treatment (pazopanib)	2015	10.6 months (Median treatment duration: 5.3 months#[14])
Cost-effectiveness analysis	32918790	First-line aRCC	Targeted treatment	Targeted treatment (sunitinib)	2021	Full treatment duration
Cost-effectiveness analysis	32918790	First-line aRCC	Targeted treatment	Targeted treatment (pazopanib)	2021	Full treatment duration
Number	Pubmed ID	Disease	Treatment	Details	Study Year	Follow-up time of AE cost
Cost-effectiveness analysis	18711190	First-line aRCC	Targeted treatment	Targeted treatment (sunitinib)	2008	Full treatment duration
Real-world	29885945	First-line aNSCLC	Chemotherapy	Chemotherapy (Carboplatin, paclitaxel)	2018	13.4 months (Median treatment duration: 3.5 months[16])
Real-world	29885945	First-line aNSCLC	Chemotherapy	Chemotherapy (Carboplatin, pemetrexed)	2018	13.4 months (Median treatment duration: 3.5

© 2025 Zhao M et al. *JAMA Network Open*.

						months[16])
Real-world	29885945	First-line aNSCLC	Chemotherapy	Chemotherapy (Carboplatin, gemcitabine)	2018	13.4 months (Median treatment duration: 3.5 months[16])
Cost-effectiveness analysis	21914503	First-line aNSCLC	Chemotherapy	Chemotherapy (carboplatin and paclitaxel)	2011	Full treatment duration
Cost-effectiveness analysis	21914503	First-line aNSCLC	Chemotherapy	Chemotherapy (platinum-based chemotherapy)	2011	Full treatment duration
Cost-effectiveness analysis	31287562	First-line aNSCLC	Chemotherapy	Chemotherapy (carboplatin and either paclitaxel or nab-paclitaxel)	2019	Full treatment duration
Cost-effectiveness analysis	32193809	First-line aNSCLC	Chemotherapy	Chemotherapy (Carboplatin, gemcitabine)	2020	Full treatment duration
Cost-effectiveness analysis	32193809	First-line aNSCLC	Chemotherapy	Chemotherapy (carboplatin and paclitaxel)	2020	Full treatment duration
Cost-effectiveness analysis	34736841	First-line aNSCLC	Chemotherapy	Chemotherapy (platinum-doublet chemotherapy)	2022	Full treatment duration
Cost-effectiveness analysis	34268373	First-line aNSCLC	Chemotherapy	Chemotherapy (platinum-doublet chemotherapy)	2021	Full treatment duration
Cost-effectiveness analysis	31945265	First-line aNSCLC	Chemotherapy	Chemotherapy (platinum-doublet chemotherapy)	2020	Full treatment duration
Cost-effectiveness analysis	31945265	First-line aNSCLC	Chemotherapy	Chemotherapy (platinum-doublet chemotherapy)	2020	Full treatment duration

© 2025 Zhao M et al. *JAMA Network Open*.

Cost-effectiveness analysis	31655368	First-line aNSCLC	Chemotherapy	Chemotherapy (platinum-doublet chemotherapy)	2019	Full treatment duration
Real-world	29983370	Second-line aNSCLC	Chemotherapy	Chemotherapy (Pemetrexed)	2018	18.6 to 20.5 months (Median treatment duration: 2-3 months[15])
Real-world	29983370	Second-line aNSCLC	Chemotherapy	Chemotherapy (Docetaxel)	2018	18.6 to 20.5 months (Median treatment duration: 2-3 months[15])
Real-world	29983370	Second-line aNSCLC	Chemotherapy	Chemotherapy (Carboplatin, Paclitaxel)	2018	18.6 to 20.5 months (Median treatment duration: 2-3 months[15])
Real-world	29983370	Second-line aNSCLC	Chemotherapy	Chemotherapy (Gemcitabine)	2018	18.6 to 20.5 months (Median treatment duration: 2-3 months[15])
Cost-effectiveness analysis	18295368	Second-line aNSCLC	Chemotherapy	Chemotherapy (Docetaxel)	2008	Full treatment duration
Cost-effectiveness analysis	18295368	Second-line aNSCLC	Chemotherapy	Chemotherapy (Pemetrexed)	2008	Full treatment duration
Real-world	29885945	First-line aNSCLC	TARGET+CHE	TARGET+CHE (Bevacizumab, carboplatin, paclitaxel)	2018	13.4 months (Median treatment duration: 2-3

© 2025 Zhao M et al. *JAMA Network Open*.

						months[17])
Cost-effectiveness analysis	21914503	First-line aNSCLC	TARGET+CHE	TARGET+CHE (bevacizumab plus carboplatin and paclitaxel)	2011	Full treatment duration
Real-world	20051811	First-line MBC	Chemotherapy	Chemotherapy (Capecitabine)	2010	Full treatment duration
Number	Pubmed ID	Disease	Treatment	Details	Study Year	Follow-up time of AE cost
Real-world	20051811	First-line MBC	Chemotherapy	Chemotherapy (Taxane+Anthracycline)	2010	Full treatment duration
Real-world	20051811	First-line MBC	Chemotherapy	Chemotherapy (Other Taxane Regimens)	2010	Full treatment duration
Real-world	27032967	First-line MBC	Chemotherapy	Chemotherapy (Chemotherapy)	2016	Full treatment duration
Real-world	25085897	First-line MBC	Chemotherapy	Chemotherapy (taxane)	2014	Full treatment duration
Real-world	25085897	First-line MBC	Chemotherapy	Chemotherapy (capecitabine)	2014	Full treatment duration
Cost-effectiveness analysis	35796042	First-line MBC	Chemotherapy	Chemotherapy (paclitaxel or nab-paclitaxel or gemcitabine plus carboplatin)	2022	Full treatment duration
Real-world	27032967	Second-line MBC	Chemotherapy	Chemotherapy (NA)	2016	Full treatment duration

© 2025 Zhao M et al. *JAMA Network Open*.

Cost-effectiveness analysis	37537015	Second-line MBC	Chemotherapy	Chemotherapy (Physician's choice for alternate chemotherapy,capecitabin/eribulin/gemcitabine/paclitaxel/nab-paclitaxel)	2023	Full treatment duration
Cost-effectiveness analysis	36532255	Second-line MBC	chemotherapy	chemotherapy (Physician's choice for alternate chemotherapy,capecitabin/eribulin/gemcitabine/paclitaxel/nab-paclitaxel)	2022	Full treatment duration
Real-world	27032967	First-line MBC	Targeted treatment	Targeted treatment (Everolimus)	2016	Full treatment duration
Cost-effectiveness analysis	36282933	First-line MBC	Targeted treatment	Targeted treatment (ribociclib+fulvestrant)	2022	Full treatment duration
Cost-effectiveness analysis	36282933	First-line MBC	Targeted treatment	Targeted treatment (Fulvestrant)	2022	Full treatment duration
Cost-effectiveness analysis	14693319	First-line MBC	Targeted treatment	Targeted treatment (tamoxifen)	2003	Full treatment duration
Cost-effectiveness analysis	14693319	First-line MBC	Targeted treatment	Targeted treatment (Anastrozole)	2003	Full treatment duration
Cost-effectiveness analysis	33364838	First-line MBC	Targeted treatment	Targeted treatment (fulvestrant)	2020	Full treatment duration
Cost-effectiveness analysis	33364838	First-line MBC	Targeted treatment	Targeted treatment (Ribociclib+fulvestrant)	2020	Full treatment duration
Real-world	2703296	Second-line MBC	Targeted treatment	Targeted treatment (Everolimus)	2016	Full treatment

© 2025 Zhao M et al. *JAMA Network Open*.

	7					duration
Cost-effectiveness analysis	3653225 5	Second-line MBC	Targeted treatment	Targeted treatment (trastuzumab deruxtecan)	2022	Full treatment duration

Abbreviation: AE, adverse event; CEA, cost-effectiveness analysis; aNSCLC, advanced non-small cell lung cancer; aMel, advanced melanoma; aRCC, advanced renal cell carcinoma; CEA, cost-effectiveness analysis; Chemo, chemotherapy; ICI, immune checkpoint inhibitor; MBC, metastatic breast cancer; uHCC, unresectable hepatocellular carcinoma.

The report focuses on median PFS (progression-free survival) and lacks information on median treatment duration, which is therefore expected to be shorter in terms of treatment duration

© 2025 Zhao M et al. *JAMA Network Open*.

eTable 10. Comparability Analysis of Real-World and CEA Evidence for Absolute AE Costs

Type	Pubmed ID	Disease	Treatment	Details	Study Year	Follow-up time of AE cost
Real-world	32463768	First-line aNSCLC	Chemotherapy	Chemotherapy (NA)	2020	6 months after end of first-line therapy
Cost-effectiveness analysis	34512315	First-line aNSCLC	Chemotherapy	Chemotherapy (NSQ:pemetrexed+cisplatin/SQ:gemcitabine+cisplatin)	2021	Full treatment duration
Cost-effectiveness analysis	36653947	First-line aNSCLC	Chemotherapy	Chemotherapy (Chemotherapy)	2023	Full treatment duration
Cost-effectiveness analysis	30268469	First-line aNSCLC	Chemotherapy	Chemotherapy (investigator's choice of platinum-based chemotherapy)	2018	Full treatment duration
Cost-effectiveness analysis	31610828	First-line aNSCLC	Chemotherapy	Chemotherapy (platinum-based chemotherapy)	2020	Full treatment duration
Cost-effectiveness analysis	34290602	First-line aNSCLC	Chemotherapy	Chemotherapy (platinum-doublet chemotherapy)	2021	Full treatment duration
Cost-effectiveness analysis	33938936	First-line aNSCLC	Chemotherapy	Chemotherapy (platinum-doublet chemotherapy)	2021	Full treatment duration
Cost-	317381	First-line	Chemotherapy	Chemotherapy (platinum-based chemotherapy)	2019	Full treatment duration

© 2025 Zhao M et al. *JAMA Network Open*.

effectiveness analysis	17	aNSCLC				
Cost-effectiveness analysis	31831534	First-line aNSCLC	Chemotherapy	Chemotherapy (pemetrexed combined platinum)	2019	Full treatment duration
Cost-effectiveness analysis	34100243	First-line aNSCLC	Chemotherapy	Chemotherapy (platinum doublet chemotherapy)	2021	Full treatment duration
Cost-effectiveness analysis	34734033	First-line aNSCLC	Chemotherapy	Chemotherapy (cisplatin/carboplatin plus gemcitabine)	2021	Full treatment duration
Type	Pubmed ID	Disease	Treatment	Details	Study Year	Follow-up time of AE cost
Cost-effectiveness analysis	33209600	First-line aNSCLC	Chemotherapy	Chemotherapy (carboplatin and either (nab)paclitaxel)	2020	Full treatment duration
Cost-effectiveness analysis	30649973	First-line aNSCLC	Chemotherapy	Chemotherapy (carboplatin and either paclitaxel or nab-paclitaxel)	2019	Full treatment duration
Cost-effectiveness analysis	31287562	First-line aNSCLC	Chemotherapy	Chemotherapy (carboplatin + paclitaxe)	2019	Full treatment duration
Cost-effectiveness analysis	31553470	First-line aNSCLC	Chemotherapy	Chemotherapy (carboplatin+pemetrexed)	2019	Full treatment duration

© 2025 Zhao M et al. *JAMA Network Open*.

Cost-effectiveness analysis	26122345	First-line aNSCLC	Chemotherapy	Chemotherapy (Carb+Pac)	2015	Full treatment duration
Cost-effectiveness analysis	26122345	First-line aNSCLC	Chemotherapy	Chemotherapy (Cis+Gem)	2015	Full treatment duration
Cost-effectiveness analysis	26122345	First-line aNSCLC	Chemotherapy	Chemotherapy (Cis+Pem)	2015	Full treatment duration
Cost-effectiveness analysis	32193809	First-line aNSCLC	Chemotherapy	Chemotherapy (carboplatin plus nab-paclitaxel)	2020	Full treatment duration
Cost-effectiveness analysis	37854153	First-line aNSCLC	Chemotherapy	Chemotherapy (docetaxel)	2023	Full treatment duration
Cost-effectiveness analysis	37854153	First-line aNSCLC	Chemotherapy	Chemotherapy (pemetrexed+cisplatin)	2023	Full treatment duration
Cost-effectiveness analysis	35659172	First-line aNSCLC	Chemotherapy	Chemotherapy (platinum-doublet chemotherapy)	2022	Full treatment duration
Type	Pubmed ID	Disease	Treatment	Details	Study Year	Follow-up time of AE cost
Cost-effectiveness	34736841	First-line aNSCLC	Chemotherapy	Chemotherapy (platinum-doublet chemotherapy)	2022	Full treatment duration

© 2025 Zhao M et al. *JAMA Network Open*.

analysis						
Cost-effectiveness analysis	31655368	First-line aNSCLC	Chemotherapy	Chemotherapy (platinum-based chemotherapy)	2019	Full treatment duration
Cost-effectiveness analysis	35094793	First-line aNSCLC	Chemotherapy	Chemotherapy (platinum-doublet chemotherapy)	2022	Full treatment duration
Cost-effectiveness analysis	35658806	First-line aNSCLC	Chemotherapy	Chemotherapy (platinum-doublet chemotherapy)	2022	Full treatment duration
Cost-effectiveness analysis	35658806	First-line aNSCLC	Chemotherapy	Chemotherapy (platinum-doublet chemotherapy)	2022	Full treatment duration
Cost-effectiveness analysis	31995619	First-line aNSCLC	Chemotherapy	Chemotherapy (carboplatin+paclitaxel)	2020	Full treatment duration
Cost-effectiveness analysis	31995619	First-line aNSCLC	Chemotherapy	Chemotherapy (carboplatin+pemetrexed)	2020	Full treatment duration
Cost-effectiveness analysis	31995619	First-line aNSCLC	Chemotherapy	Chemotherapy (docetaxel)	2020	Full treatment duration
Cost-effectiveness analysis	32524512	First-line aNSCLC	Chemotherapy	Chemotherapy (carboplatin plus nab-paclitaxel chemotherapy)	2020	Full treatment duration

© 2025 Zhao M et al. *JAMA Network Open*.

Cost-effectiveness analysis	32811247	First-line aNSCLC	Chemotherapy	Chemotherapy (platinum-doublet chemotherapy)	2020	Full treatment duration
Cost-effectiveness analysis	33014826	First-line aNSCLC	Chemotherapy	Chemotherapy (platinum-doublet chemotherapy)	2020	Full treatment duration
Real-world	32463768	First-line aNSCLC	ICI	ICI (NA)	2020	6 months after end of first-line therapy
Type	Pubmed ID	Disease	Treatment	Details	Study Year	Follow-up time of AE cost
Cost-effectiveness analysis	34512315	First-line aNSCLC	ICI	ICI (Nivolumab+ipilimumab)	2021	Full treatment duration
Cost-effectiveness analysis	36653947	First-line aNSCLC	ICI	ICI (Pembrolizumab)	2023	Full treatment duration
Cost-effectiveness analysis	30268469	First-line aNSCLC	ICI	ICI (Pembrolizumab)	2018	Full treatment duration
Cost-effectiveness analysis	31610828	First-line aNSCLC	ICI	ICI (Pembrolizumab)	2020	Full treatment duration
Cost-effectiveness analysis	34290602	First-line aNSCLC	ICI	ICI (nivolumab plus ipilimumab)	2021	Full treatment duration

© 2025 Zhao M et al. *JAMA Network Open*.

Cost-effectiveness analysis	33938936	First-line aNSCLC	ICI	ICI (Nivolumab-ipilimumab)	2021	Full treatment duration
Cost-effectiveness analysis	31738117	First-line aNSCLC	ICI	ICI (pembrolizumab)	2019	Full treatment duration
Cost-effectiveness analysis	34734033	First-line aNSCLC	ICI	ICI (Atezolizumab)	2021	Full treatment duration
Cost-effectiveness analysis	35659172	First-line aNSCLC	ICI	ICI (ipilimumab+nivolumab)	2022	Full treatment duration
Cost-effectiveness analysis	34736841	First-line aNSCLC	ICI	ICI (cemiplimab)	2022	Full treatment duration
Cost-effectiveness analysis	35094793	First-line aNSCLC	ICI	ICI (cemiplimab)	2022	Full treatment duration
Type	Pubmed ID	Disease	Treatment	Details	Study Year	Follow-up time of AE cost
Cost-effectiveness analysis	35094793	First-line aNSCLC	ICI	ICI (Pembrolizumab)	2022	Full treatment duration
Cost-effectiveness	35658806	First-line aNSCLC	ICI	ICI (nivolumab plus ipilimumab)	2022	Full treatment duration

© 2025 Zhao M et al. *JAMA Network Open*.

analysis						
Cost-effectiveness analysis	31995619	First-line aNSCLC	ICI	ICI (pembrolizumab)	2020	Full treatment duration
Cost-effectiveness analysis	32811247	First-line aNSCLC	ICI	ICI (Nivolumab plus ipilimumab)	2020	Full treatment duration
Real-world	32463768	First-line aNSCLC	ICI+CHE	ICI+CHE (NA)	2020	6 months after end of first-line therapy
Cost-effectiveness analysis	36653947	First-line aNSCLC	ICI+CHE	ICI+CHE (atezolizumab plus chemotherapy)	2023	Full treatment duration
Cost-effectiveness analysis	36653947	First-line aNSCLC	ICI+CHE	ICI+CHE (nivolumab plus ipilimumab combined with chemotherapy)	2023	Full treatment duration
Cost-effectiveness analysis	36653947	First-line aNSCLC	ICI+CHE	ICI+CHE (pembrolizumab pemetrexed combined platinum)	2023	Full treatment duration
Cost-effectiveness analysis	31831534	First-line aNSCLC	ICI+CHE	ICI+CHE (pembrolizumab pemetrexed combined platinum)	2019	Full treatment duration
Cost-effectiveness analysis	34100243	First-line aNSCLC	ICI+CHE	ICI+CHE (nivolumab plus ipilimumab combined with chemotherapy)	2021	Full treatment duration
Cost-	332096	First-line	ICI+CHE	ICI+CHE (pembrolizumab plus chemotherapy)	2020	Full treatment duration

© 2025 Zhao M et al. *JAMA Network Open*.

effectiveness analysis	00	aNSCLC				
Cost-effectiveness analysis	30649973	First-line aNSCLC	ICI+CHE	ICI+CHE (Pembrolizumab + Chemotherapy)	2019	Full treatment duration
Type	Pubmed ID	Disease	Treatment	Details	Study Year	Follow-up time of AE cost
Cost-effectiveness analysis	31553470	First-line aNSCLC	ICI+CHE	ICI+CHE (pembrolizumab+carboplatin+pemetrexed)	2019	Full treatment duration
Cost-effectiveness analysis	35658806	First-line aNSCLC	ICI+CHE	ICI+CHE (nivolumab plus ipilimumab combined with chemotherapy)	2022	Full treatment duration
Cost-effectiveness analysis	31995619	First-line aNSCLC	ICI+CHE	ICI+CHE (pembrolizumab+carboplatin+paclitaxel)	2020	Full treatment duration
Cost-effectiveness analysis	31995619	First-line aNSCLC	ICI+CHE	ICI+CHE (pembrolizumab+carboplatin+pemetrexed)	2020	Full treatment duration
Cost-effectiveness analysis	32524512	First-line aNSCLC	ICI+CHE	ICI+CHE (atezolizumab plus chemotherapy)	2020	Full treatment duration
Cost-effectiveness analysis	33014826	First-line aNSCLC	ICI+CHE	ICI+CHE (Nivolumab plus ipilimumab)	2020	Full treatment duration

© 2025 Zhao M et al. *JAMA Network Open*.

Real-world	34494389	First-line uHCC	Targeted treatment	Targeted treatment (Sorafenib)	2021	12 months (Median treatment duration: 5.3 months[18])
Cost-effectiveness analysis	33625508	First-line uHCC	Targeted treatment	Targeted treatment (Sorafenib)	2021	Full treatment duration
Cost-effectiveness analysis	33668100	First-line uHCC	Targeted treatment	Targeted treatment (Sorafenib)	2021	Full treatment duration
Cost-effectiveness analysis	33556230	First-line uHCC	Targeted treatment	Targeted treatment (Sorafenib)	2021	Full treatment duration
Cost-effectiveness analysis	33417490	First-line uHCC	Targeted treatment	Targeted treatment (Sorafenib)	2021	Full treatment duration
Type	Pubmed ID	Disease	Treatment	Details	Study Year	Follow-up time of AE cost
Cost-effectiveness analysis	33825837	First-line uHCC	Targeted treatment	Targeted treatment (Sorafenib)	2021	Full treatment duration
Real-world	25085897	First-line MBC	Chemotherapy	Chemotherapy (Capecitabine)	2014	Full treatment duration
Real-world	25085897	First-line MBC	Chemotherapy	Chemotherapy (taxane)	2014	Full treatment duration
Cost-effectiveness	32522057	First-line MBC	Chemotherapy	Chemotherapy (nab-paclitaxel)	2020	Full treatment duration

© 2025 Zhao M et al. *JAMA Network Open*.

analysis						
Cost-effectiveness analysis	32426048	First-line MBC	Chemotherapy	Chemotherapy (nab-paclitaxel)	2020	Full treatment duration
Cost-effectiveness analysis	35796042	First-line MBC	Chemotherapy	Chemotherapy (One of paclitaxel, nab-paclitaxel, and gemcitabine plus carboplatin)	2021	Full treatment duration
Real-world	25085897	Second-line MBC	Chemotherapy	Chemotherapy (taxane)	2014	Full treatment duration
Real-world	25085897	Second-line MBC	Chemotherapy	Chemotherapy (Capecitabine)	2014	Full treatment duration
Cost-effectiveness analysis	37085377	Second-line MBC	Chemotherapy	Chemotherapy (physician's choice of capecitabine, eribulin, gemcitabine, paclitaxel, or nab-paclitaxel)	2023	Full treatment duration
Cost-effectiveness analysis	36780838	Second-line MBC	Chemotherapy	Chemotherapy (eribulin, vinorelbine, capecitabine and gemcitabine, which is determined based on physician’s choice)	2023	Full treatment duration
Cost-effectiveness analysis	37537015	Second-line MBC	Chemotherapy	Chemotherapy (Physician’s choice for alternate chemotherapy,capecitabin/eribulin/gemcitabine/paclitaxel/nab-paclitaxel)	2023	Full treatment duration
Type	Pubmed ID	Disease	Treatment	Details	Study Year	Follow-up time of AE cost
Cost-	373866	Second-	Chemotherapy	Chemotherapy (54% eribulin,20% vinorelbine, 13%	2023	Full treatment duration

© 2025 Zhao M et al. *JAMA Network Open*.

effectiveness analysis	33	line MBC		capecitabine, or 12% gemcitabine)		
Cost-effectiveness analysis	34778047	Second-line MBC	Chemotherapy	Chemotherapy (54% eribulin,20% vinorelbine, 13% capecitabine, or 12% gemcitabine)	2021	Full treatment duration
Cost-effectiveness analysis	36532255	Second-line MBC	Chemotherapy	Chemotherapy (Physician's choice for alternate chemotherapy,capecitabin/eribulin/gemcitabine/paclitaxel/nab-paclitaxel)	2022	Full treatment duration

Abbreviation: AE, adverse event; CEA, cost-effectiveness analysis; aNSCLC, advanced non-small cell lung cancer; aMel, advanced melanoma; aRCC, advanced renal cell carcinoma; CEA, cost-effectiveness analysis; CHE, chemotherapy; ICI, immune checkpoint inhibitor; MBC, metastatic breast cancer; uHCC, unresectable hepatocellular carcinomaeTable 7 Detailed information for AE Cost Impact on ICER

© 2025 Zhao M et al. *JAMA Network Open*.

eTable 11. Detailed information for AE Cost Impact on ICER

Abbreviation: AE, adverse event; CEA, cost-effectiveness analysis; CHE, chemotherapy; ICI, immune checkpoint inhibitor; ICER, incremental cost-effectiveness ratio; ICER-

PMID	Intervention	CEA AE cost	AE-real Cost	Overall Cost	QALY	Control	CEA AE cost	AE-real cost	Overall Cost	QALY	ICER-Ori	ICER-Real
34290602	ICI	1499	16319	318624	2.832	Chemotherapy	1259	23009	223007.00	1.57	75887	88686
33938936	ICI	1185	16319	377400	1.68	Chemotherapy	6384	23009	175500.00	1.18	403800	200409
31831534	ICI+CHE	3538	18806	288532	1.61	Chemotherapy	3005	23009	137123.00	0.83	194114	146672
34100243	ICI+CHE	2519.16	18806	264,278	1.78	Chemotherapy	4565.52	23009	102284.00	0.98	202493	159837
34734033	ICI	421.209	16319	257618	1.75	Chemotherapy	8388.163	23009	150529.00	0.88	123091	108365
33209600	ICI+CHE	1057	18806	341637	2.566	Chemotherapy	1329	23009	198863.00	1.40	122238	138842
33209600	ICI+CHE	1098	18806	283797	2.386	Chemotherapy	1378	23009	159481.00	1.36	121402	120392
30649973	ICI+CHE	1499	18806	232962	2.72	Chemotherapy	1259	23009	105038.00	1.44	99941	123480
32193809	ICI+CHE	14287.28	18806	368812	1.68	Chemotherapy	11984.73	23009	259003	1.52	686306	103303
35659172	ICI	155	16319	262689	2.89	Chemotherapy	4046	23009	120601.00	1.56	106833	139288
34736841	ICI	88.39	16319	224663	2.05	Chemotherapy	2911	23009	126451.94	0.98	91786	94343
35094793	ICI	237	16319	234233	2.91	Chemotherapy	5265	23009	75106.00	1.13	89397	157463
35658806	ICI+CHE	1881	18806	317,581	2.86	Chemotherapy	2762	23009	119909.00	1.37	132666	194349
35658806	ICI	153	16319	256,414	2.86	Chemotherapy	3748	23009	117217.00	1.37	93421	136101
32524512	ICI+CHE	8138.92	18806	207,962	0.99	Chemotherapy	6594.586	23009	102345.00	0.67	330053	99869
32811247	ICI+CHE	885.36	18806	367,733	2.379	Chemotherapy	4774.836	23009	238749.00	1.66	180397	128670
33014826	ICI+CHE	662.87	18806	390218.01	2.81	Chemotherapy	6412.183	23009	266037.25	1.66	107983	125726

Ori, originally reported ICER; ICER-Real, ICER adjusted for AE costs using Real-cost

© 2025 Zhao M et al. *JAMA Network Open*.

eFigure 3. Variability in the Cost of Unit Adverse Events Among Cost-effectiveness Analyses

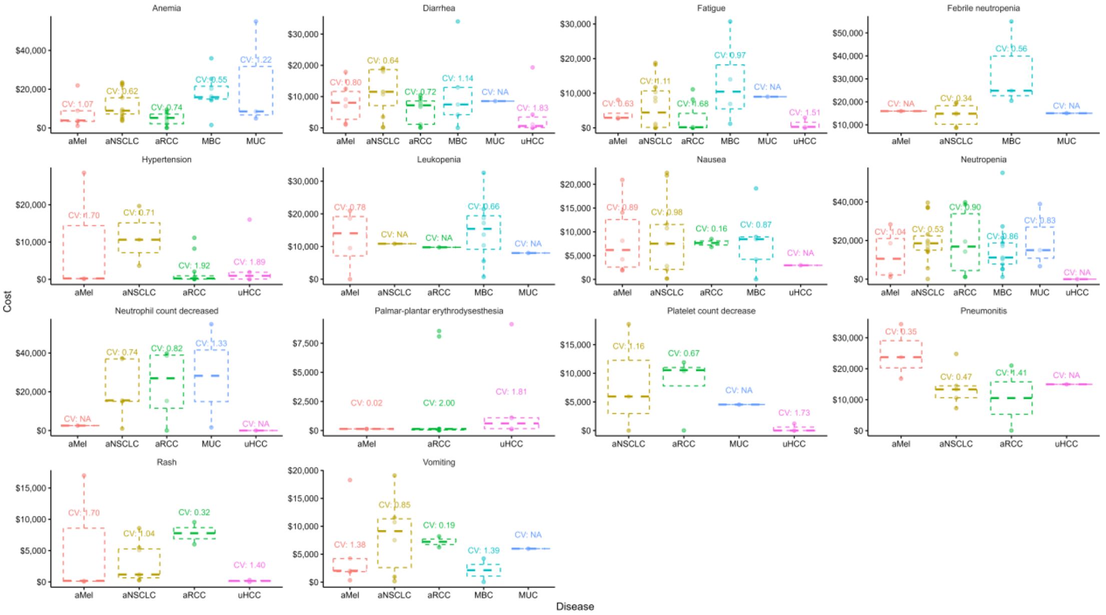

Abbreviation: aNSCLC, advanced non-small cell lung cancer; aMel, advanced melanoma; aRCC, advanced renal cell carcinoma; CV, Coefficient of Variation; DI, Dispersion Index; MBC, metastatic breast cancer; MUC, metastatic urothelial cancer; TD, targeted drug; uHCC, unresectable hepatocellular carcinoma

[a] High dispersion defined as CV > 0.3 or DI > 1

[b] Horizontal line represents median. Box shows interquartile range (IQR), and whiskers extend to 1.5 times IQR from quartiles

[c] Points represent unit costs of specific adverse events

© 2025 Zhao M et al. *JAMA Network Open*.

eFigure 4. Proportion of Adverse Event Costs for Specific Drugs: Cost-Effectiveness Analysis vs Real-World Values

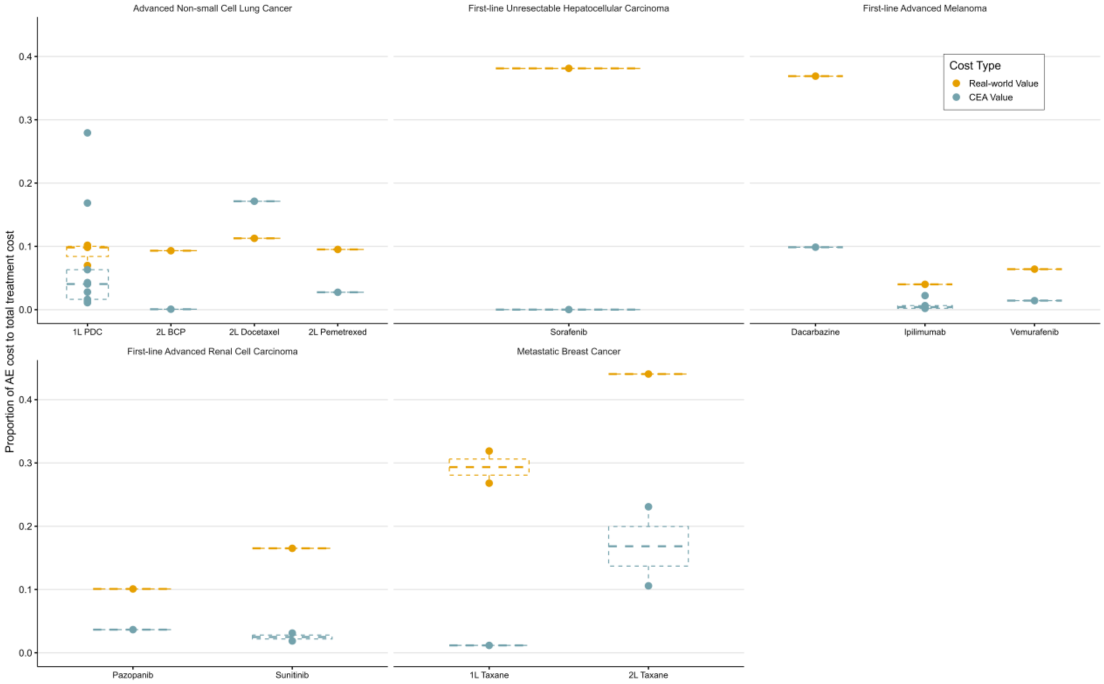

Abbreviation: 1L, first-line; 2L, second-line; AE, adverse event; BCP, Bevacizumab with Chemotherapy; CEA, cost-effectiveness analysis; PDC, Platinum-based Doublet Chemotherapy

[a] Each point represents a comparison between the real-world value and the CEA value, conducted under the same treatment and indication

[b] Horizontal line represents median. Box shows interquartile range (IQR), and whiskers extend to 1.5 times IQR from quartiles

© 2025 Zhao M et al. *JAMA Network Open*.

eTable 12. Good practice recommendations

Issue	Good Practice Recommendations for Improving AE Cost Quantification
Adverse Event Inclusion	1.The CHEERS checklist needs stricter items to ensure researchers' AE reporting aligns with inclusion criteria, covering AE types and incidence rates<br>2. AEs included should be treatment-related, not all-cause<br>3. It's best to include all TRAEs, not just grade 3+/severe AEs<br>4. If an AE must be excluded, explain why and its limited impact on results; conduct necessary sensitivity analyses.<br>5. Include AE management costs for post-progression treatments
AE-related Dose Modifications	1. Consider dose modifications as necessary, since AE-related treatment interruptions and reductions are common and persist<br>2. Without individual data, modeling dose reduction and interruption rates per cycle is challenging. Relative dose intensity may be an ideal alternative<br>3.If sufficient data is unavailable, ignoring dose adjustments' impact on efficacy is acceptable. But with sufficient data (e.g., individual patient data), model differential risks related to the treatment's risk profile and dose adjustments to increase simulation flexibility
AE Cost Calculation Method and Sources of Unit AE Costs	1. Management costs for different grades of AE should be calculated separately, especially for grade 1-2 and grade 3+<br>2. Differentiate between short-term and long-term AEs, if sufficient data is available.<br>3. To better quantify multiple AE costs, when individual data or AE incidence curves for different cycles are available, AE management costs within the study period can be directly calculated from existing data. Use multi-parameter models to fit and extrapolate existing AE time-incidence curves for long-term cost estimation.<br>4.If the above data is lacking, assuming all AEs occur in the first cycle and only once is acceptable, with necessary uncertainty analyses<br>5. Justify the sources of unit AE costs and conduct necessary uncertainty analyses when multiple sources are available.
Others	1. CEAs should use high-quality real-world data to estimate AE costs, prioritizing standardized long-term records for drugs with available evidence. For innovative drugs lacking such data, HTA agencies should reevaluate when evidence becomes available to accurately assess AE impacts.<br>2. If real-world costs for the drug are available and align with the CEA perspective, they should be preferred<br>2. Establish guidelines to standardize AE inclusion, dose modifications, cost calculation, and sources of unit costs to reduce bias in AE cost quantification in current CEA practices<br>3. Researchers should increase transparency by reporting AE inclusion criteria, types and incidence of AEs, calculation methods, and how dose adjustments are modeled. They should also provide absolute cost estimates of AEs to aid decision-makers in resource allocation and commit to improving the evidence base

© 2025 Zhao M et al. *JAMA Network Open*.

Abbreviation: AE, adverse event; CEA, cost-effectiveness analysis; TRAE, treatment-related adverse event.

© 2025 Zhao M et al. *JAMA Network Open*.

eReferences

[1] McGregor B, Geynisman D M, Burotto M, et al. Grade 3/4 Adverse Event Costs of Immuno-oncology Combination Therapies for Previously Untreated Advanced Renal Cell Carcinoma[J]. Oncologist, 2023,28(1):72-79.

[2] Common Terminology Criteria for Adverse Events (CTCAE)[EB/OL]. [23 July]. https://evs.nci.nih.gov/ftp1/CTCAE/CTCAE_4.03/Archive/CTCAE_4.0_2009-05-29_QuickReference_8.5x11.pdf.

[3] Hryniuk W, Bush H. The importance of dose intensity in chemotherapy of metastatic breast cancer[J]. J Clin Oncol, 1984,2(11):1281-1288.

[4] Jabbour E, Kantarjian H M, Quintas-Cardama A, et al. Impact of Dose Reductions and Interruptions Due to Adverse Events (AEs) on Efficacy in Newly Diagnosed Chronic Myeloid Leukemia in ChronicPhase (CML-CP) Patients (pts) Receiving Either Dasatinib (D) or Imatinib(IM): Analysis of the DASISION Trial[J]. Blood, 2011,118(21):2768.

[5] Borghaei H, Yim Y M, Guerin A, et al. Severe adverse events impact overall survival and costs in elderly patients with advanced non-small cell lung cancer on second-line therapy[J]. Lung Cancer, 2018,119:112-119.

[6] Rugo H S, Andre F, Yamashita T, et al. Time course and management of key adverse events during the randomized phase III SOLAR-1 study of PI3K inhibitor alpelisib plus fulvestrant in patients with HR-positive advanced breast cancer[J]. Ann Oncol, 2020,31(8):1001-1010.

[7] Estimating the cost of adverse events in economic models[EB/OL]. [23 July]. https://www.ispor.org/docs/default-source/presentations/1454.pdf?sfvrsn=4855316c_1.

[8] Estimating the Costs of Adverse Events in Economic Models: Is There a “Right” Approach?[EB/OL]. [23 July]. https://www.ispor.org/publications/journals/value-outcomes-spotlight/abstract/may-june-2019/estimating-the-costs-of-adverse-events-in-economic-models-is-there-a-right-approach.

[9] Davis S, Stevenson M, Tappenden P, et al. NICE DSU Technical Support Document 15: Cost-Effectiveness Modelling Using Patient-Level Simulation[M]. London: National Institute for Health and Care Excellence (NICE), 2014.

[10] Sullivan S D, Mauskopf J A, Augustovski F, et al. Budget impact analysis-principles of good practice: report of the ISPOR 2012 Budget Impact Analysis Good Practice II Task Force[J]. Value Health, 2014,17(1):5-14.

[11] Pearce A, Haas M, Viney R. Are the true impacts of adverse events considered in economic models of antineoplastic drugs? A systematic review[J]. Appl Health Econ Health Policy, 2013,11(6):619-637.

[12] Korn EL, Liu PY, Lee SJ, et al.. Meta-analysis of phase II cooperative group trials in metastatic stage IV melanoma to determine progression-free and overall survival benchmarks for future phase II trials. J Clin Oncol. 2008 Feb 1;26(4):527-34. doi: 10.1200/JCO.2007.12.7837. PMID: 18235113.

[13] Hodi FS, O'Day SJ, McDermott DF, et al. Improved survival with ipilimumab in patients with metastatic melanoma. N Engl J Med. 2010 Aug 19;363(8):711-23. doi: 10.1056/NEJMoa1003466. Epub 2010 Jun 5. Erratum in: N Engl J Med. 2010 Sep 23;363(13):1290.

[14] Chapman PB, Hauschild A, Robert C, et al; BRIM-3 Study Group. Improved survival with

© 2025 Zhao M et al. *JAMA Network Open*.

vemurafenib in melanoma with BRAF V600E mutation. N Engl J Med. 2011 Jun 30;364(26):2507-16. doi: 10.1056/NEJMoa1103782. Epub 2011 Jun 5. PMID: 21639808; PMCID: PMC3549296.

[15] Hanna N, Shepherd FA, Fossella FV, et al. Randomized phase III trial of pemetrexed versus docetaxel in patients with non-small-cell lung cancer previously treated with chemotherapy. J Clin Oncol. 2004 May 1;22(9):1589-97. doi: 10.1200/JCO.2004.08.163. PMID: 15117980.

[16] Scagliotti GV, Parikh P, von Pawel J, et al. Phase III study comparing cisplatin plus gemcitabine with cisplatin plus pemetrexed in chemotherapy-naive patients with advanced-stage non-small-cell lung cancer. J Clin Oncol. 2008 Jul 20;26(21):3543-51. doi: 10.1200/JCO.2007.15.0375. Epub 2008 May 27. PMID: 18506025.

[17] Sandler A, Gray R, Perry MC, et al. Paclitaxel-carboplatin alone or with bevacizumab for non-small-cell lung cancer. N Engl J Med. 2006 Dec 14;355(24):2542-50. doi: 10.1056/NEJMoa061884. Erratum in: N Engl J Med. 2007 Jan 18;356(3):318. PMID: 17167137..

[18] Llovet JM, Ricci S, Mazzaferro V, et al; SHARP Investigators Study Group. Sorafenib in advanced hepatocellular carcinoma. N Engl J Med. 2008 Jul 24;359(4):378-90..

© 2025 Zhao M et al. *JAMA Network Open*.
