# Supplementary material for: Adverse Event Costs and Cost-Effectiveness Analyses of Anticancer Drugs: A Systematic Review
Source: JAMA Netw Open. 2025 May 27;8(5):e2512455. doi: 10.1001/jamanetworkopen.2025.12455 (PMC12117467; doi:10.1001/jamanetworkopen.2025.12455)
Supplement: Supplement 2. — Data Sharing Statement [file jamanetwopen-e2512455-s002.pdf]

Data Sharing Statement

Zhao. Adverse Event Costs and Impact in Cost-Effectiveness Analyses of Anticancer Drugs. *JAMA Netw Open*. Published May 27, 2025. doi:10.1001/jamanetworkopen.2025.12455

Data
Data available: Yes
Data types: Data (not involving human participants)
How to access data: tokammy@cpu.edu.cn
When available: With publication

Supporting Documents
Document types: None

Additional Information
Who can access the data: Data will be available to researchers whose proposed use of the data has been approved.
Types of analyses: For specified purpose as discussed with the corresponding author
Mechanisms of data availability: With a signed data access agreement
Any additional restrictions: Data will be available up to 1 year after publication
